# Different dosages of vonoprazan for gastroesophageal reflux disease: study protocol for a pragmatic, crossover-cluster, randomized controlled trial with patient preference arms

**DOI:** 10.1186/s13063-023-07760-9

**Published:** 2023-12-01

**Authors:** Dongke Wang, Dan Zhou, Xinghuang Liu, Zhiyue Xu, Tao Bai, Xiaohua Hou

**Affiliations:** Division of Gastroenterology, Union Hospital, Tongji Medical College, Huazhong University of Science and Technology, Wuhan, China

**Keywords:** Gastroesophageal reflux disease, Potassium-competitive acid blocker, Pragmatic trial

## Abstract

**Background:**

Vonoprazan results in more potent acid suppression for gastroesophageal reflux disease (GERD) than proton pump inhibitors. It has only been approved for treating erosive esophagitis in China, but 30–40% of GERD patients cannot achieve the goal of treatment with vonoprazan 20 mg daily. This study aims to investigate whether vonoprazan could relieve the symptoms of Chinese patients with non-erosive reflux disease (NERD) and whether increased dosage or different times of dosing could increase the response rate of GERD.

**Methods:**

This study is a pragmatic, open-label, crossover-cluster, randomized controlled trial with patient preference arms. Two thousand eight hundred eighty patients with GERD from 48 hospitals in China will be enrolled. These hospitals will be divided into a compulsory randomization cluster (24 hospitals) and a patient preference cluster (24 hospitals). Patients in the compulsory randomization cluster will be randomized to three regimens according to the crossover-cluster randomization. Patients in the patient preference cluster may choose to receive any regimen if they have a preference; otherwise, patients will be randomly assigned. The three treatment regimens will last 4 weeks, including (1) vonoprazan 20 mg p.o. after breakfast, (2) vonoprazan 20 mg p.o. after dinner, and (3) vonoprazan 20 mg p.o. after breakfast and after dinner. Patients will attend a baseline visit, a 4-week e-diary, a fourth-week visit, and a sixth-month visit online. The primary outcome is the symptom relief rate of all patients after 4-week therapy. Secondary outcomes include the healing rate of EE patients, the severity of symptoms, compliance with the therapy at the fourth-week follow-up visit, recurrent symptoms, and the frequency of self-conscious doctor visits at the sixth-month follow-up visit.

**Discussion:**

This trial will explore the effectiveness of different regimens of vonoprazan that will be implemented with GERD patients in China. The randomization with patient preferences considered and the crossover-cluster component may improve the robustness and extrapolation of study conclusions.

**Trial registration:**

https://www.chictr.org.cn ChiCTR2300069857. Registered on 28 March 2023. Protocol version: February 18, 2023, Version 2.

**Supplementary Information:**

The online version contains supplementary material available at 10.1186/s13063-023-07760-9.

## Administrative information

Note: This protocol’s numbers in curly brackets refer to SPIRIT checklist item numbers. The order of the items has been modified to group similar items (see http://www.equator-network.org/reporting-guidelines/spirit-2013-statement-defining-standard-protocol-items-for-clinical-trials/).

**Table:** 

Title {1}	Different dosages of vonoprazan for gastroesophageal reflux disease: Study protocol for a pragmatic, crossover-cluster, randomized controlled trial with patient preference arms
Trial registration {2a and 2b}.	Trial identifier: ChiCTR2300069857 Registry name: Chinese Clinical Trial Registry Vonoprazan for gastroesophageal reflux disease: Study protocol for a pragmatic, crossover-cluster, randomized controlled trial
Protocol version {3}	Date: February 18, 2023 Version identifier: Version 2
Funding {4}	None
Author details {5a}	Division of Gastroenterology, Union Hospital, Tongji Medical College, Huazhong University of Science and Technology.
Name and contact information for the trial sponsor {5b}	Name: Union Hospital, Tongji Medical College, Huazhong University of Science and Technology. Contact information: 027-85726114
Role of sponsor {5c}	Role of study sponsor: study design; management and interpretation of data; checking of the study report; the decision to submit the report for publication; and the ultimate authority over any of these activities.

## Introduction

### Background and rationale {6a}

Gastroesophageal reflux disease (GERD) is a common disorder characterized by heartburn or acid regurgitation as a result of reflux of the gastric contents, classified as erosive esophagitis (EE) or non-erosive reflux disease (NERD) [1]. The pooled prevalence of GERD reported in a meta-analysis was 10.0% in Asia and 2.5% in China [2]. PPIs are recommended as first-line therapy to treat GERD. However, some unmet needs exist, such as nocturnal acid breakthrough and CYP2C19 phenotype-related variability in efficacy.

Vonoprazan is currently indicated for the treatment of acid-related diseases, such as gastric and duodenal ulcers, *Helicobacter pylori* infection, and GERD [3–5]. Vonoprazan is a novel potassium-competitive acid blocker (PCAB) that blocks H^+^/K^+^ ATPase competitively and reversibly [6]. It is rapidly absorbed and reaches the maximum plasma level within 2 h. However, the plasma half-life (*t*_1/2_) was much longer (*t*_1/2_: about 7 h with 20 mg of vonoprazan) than conventional proton-pump inhibitors (PPIs) (*t*_1/2_: about 1–2 h), and the pH > 4 holding time of vonoprazan (20–40 mg) was longer than conventional PPIs [7]. Indeed, vonoprazan results in a more rapid onset of action, longer duration of action, and more sustained acid suppression, irrespective of CYP2C19 [7, 8].

### Vonoprazan could be used on NERD patients

Several researches showed that vonoprazan, compared with PPIs, could better relieve symptoms and heal mucosal for GERD patients [5, 9–11]. In the multicenter study of Asian participants, EE healing rates appeared higher with vonoprazan versus lansoprazole [5]. Complete sustained heartburn relief was achieved sooner with vonoprazan than lansoprazole in Japanese participants [12]. The benefit may be due to the more potent and longer-lasting inhibition of acid secretion provided by vonoprazan [13, 14].

More than 50% of GERD exhibit normal esophageal mucosa on upper endoscopy, diagnosed as NERD, which is also acid-related [15, 16]. However, reflux symptoms of patients with NERD were not entirely resolved in approximately half of them treated with PPIs [17, 18]. A previous study in Japan showed that the mean severity of heartburn is lower with vonoprazan compared with placebo in patients with NERD [19]. Although vonoprazan has only been approved for the treatment of EE in China [20], it is still unknown for the resolution of symptoms of Chinese patients with NERD when treated with vonoprazan.

### The clinical benefit of increased dosage is worth discovering

Complete sustained heartburn relief was achieved in 62.5% of GERD patients with vonoprazan 20 mg daily during the first week of therapy, and the proportion of patients without heartburn at week 8 was 70.5% [5, 12]. The rate of endoscopic healing of EE with vonoprazan 20 mg daily during the second week, fourth-week, and eighth-week treatment period was 75%, 85.3%, and 92.4%, respectively [5]. Herein, 30–40% of GERD patients cannot achieve the goal of treatment with vonoprazan 20 mg daily.

A single dose of vonoprazan 1–120 mg in healthy volunteers was well-tolerated, producing a rapid, profound, and dose-dependent suppression of 24-h gastric acid secretion [21]. In patients with GERD using a range of acid-suppressing drugs with different mechanisms of action, acid-suppressed time of intra-gastric and intra-esophageal pH > 4 is a good predictor of healing and symptom relief [22–25]. In healthy Japanese volunteers, the pH > 4 holding time of vonoprazan 20 mg twice daily was potently higher than vonoprazan 20 mg once daily [14]. As vonoprazan 20 mg twice daily provides more potent acid suppression, the potential of higher symptom relief rates and healing rates of GERD patients are worth exploring.

### Different dosing times could be a predictive factor of clinical effectiveness

Approximately 80% of GERD patients with frequent heartburn develop it at night [26, 27]. Treatment failures of night symptoms on PPIs result from NAB, which might be provoked by class-specific factors of PPIs, including short t_1/2_ and irreversible binding to H^+^/K^+^ ATPase, which result in reduced exposure to proton pumps synthesized at night [28, 29]. In addition, gastric acid secretion has a circadian profile that peaks between 10 PM and 2 AM, which may represent a fundamental cause of this phenomenon [30]. In Japan, when EE patients with nocturnal symptoms take vonoprazan 20 mg daily before breakfast, only 31.3% achieved complete relief of nocturnal heartburn on day 1 [12]. When healthy Korean volunteers took vonoprazan 20 mg daily at around 10 PM for at least 3 h of fasting, the gastric acid suppression at night (% time at pH ≥ 4) was 60.5% during the post-dose pH monitoring on day 1 [31]. A single dose of vonoprazan (20 mg) can increase the intragastric pH to almost seven within 4 h, and the mean elimination *t*_1/2_ was up to 9 h [22]. The failures of night symptom relief may be due to the interval from taking vonoprazan to night symptoms being longer than elimination *t*_1/2_ of vonoprazan. Therefore, the night symptom relief may benefit from the night dosing time.

The study protocol has been registered at the Chinese Clinical Trial Registry (ChiCTR2300069857). We adhered to Consolidated Standards of Reporting Trials (CONSORT) and Standard Protocol Items: Recommendations for Interventional Trials (SPIRIT) guidelines in the design of the protocol [32, 33].

## Objectives {7}

In summary, vonoprazan for treating GERD still requires evidence regarding the following critical questions: Could vonoprazan be used on NERD patients in China? Could increase dosage and different timing of vonoprazan treatment benefit GERD patients in China? We hypothesize that (1) vonoprazan could relieve the symptoms of Chinese patients with NERD, (2) increased dosage could increase the symptom relief rate and healing rate of GERD, and (3) taking vonoprazan once daily around dinner will result in better nocturnal symptom resolution of GERD. We initiated a pragmatic, open-label, crossover-cluster, randomized-controlled trial (RCT) with patient-preference arms to validate the hypotheses.

## Trial design {8}

**Fig. 1:**
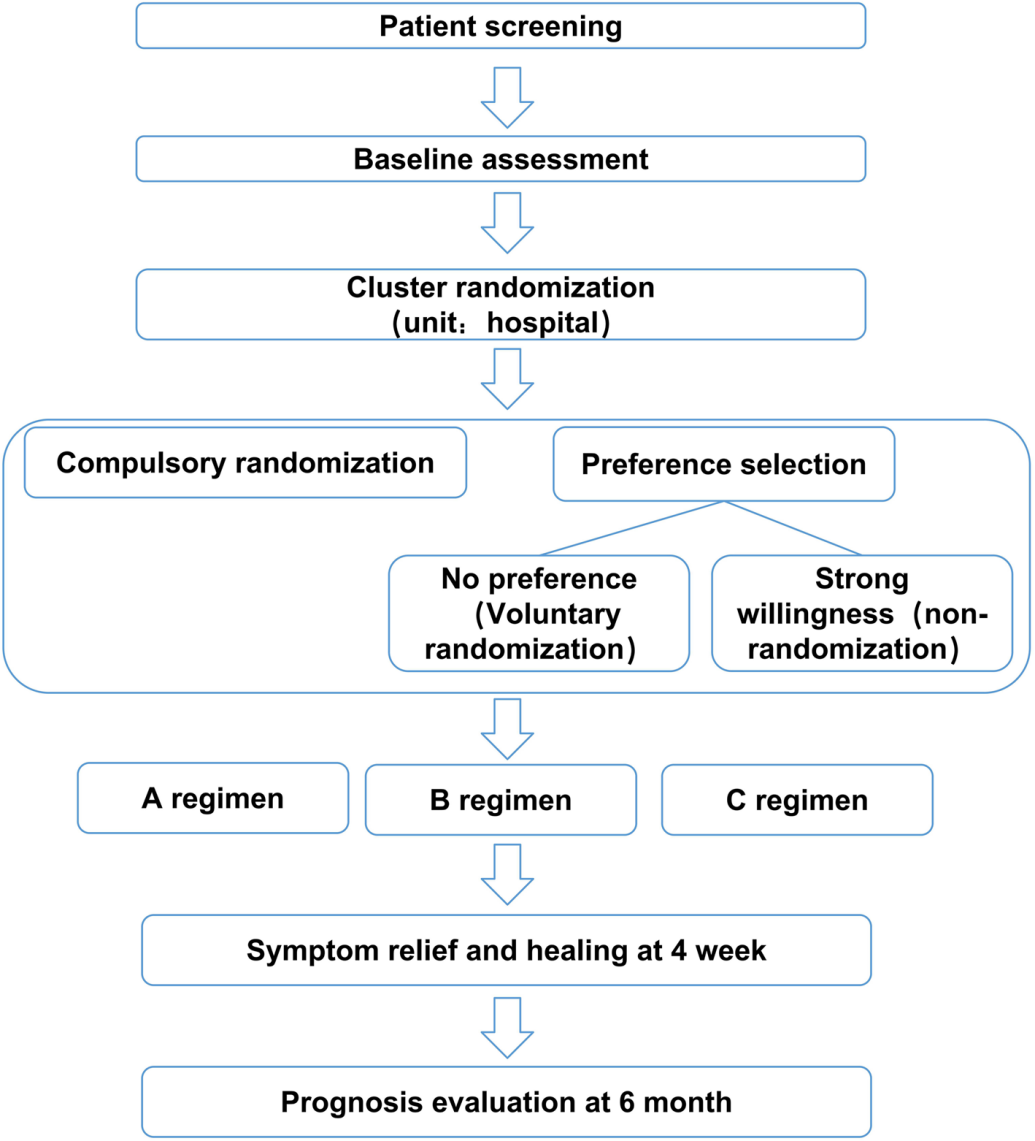
Flow chart of the study. (1) A regimen: vonoprazan 20 mg p.o. after breakfast; (2) B regimen: vonoprazan 20 mg p.o. after dinner; (3) C regimen: vonoprazan 20 mg p.o. after breakfast and after dinner

This study will be a multicenter, pragmatic, crossover-cluster RCT with patient-preference arms. After screening, 48 eligible hospitals in China are the sites of randomization. These hospitals will first be divided into two sets: a compulsory randomization cluster (24 hospitals) and a patient preference cluster (24 hospitals) by cluster randomization (Fig. 1). Three interventions will be implemented among participants, and both non-inferiority and superiority tests will be done in the statistical analysis.

## Methods: participants, interventions, and outcomes

### Study setting {9}

After conducting unified and standardized training, 48 eligible academic hospitals passed the training and assessment and are ultimately included in this study (Table 1).

**Table 1:** Study sites of this study

Number	Hospital	Country	Number	Hospital	Country
1	Wuhan Union Hospital of Huazhong University of Science and Technology	China	25	Wuhan Hospital of Traditional Chinese Medicine	China
2	Wuhan Tongji Hospital of Huazhong University of Science and Technology	China	26	The First People’s Hospital of Jiangxia District.Wuhan City	China
3	Wuhan Liyuan Hospital of Huazhong University of Science and Technology	China	27	People’s Hospital of Dongxihu Distric	China
4	General Hospital of the Central Theater Command	China	28	Wuhan Asia Heart Hospital	China
5	Zhongnan Hospital of Wuhan University	China	29	Center People’s Hospital of Yichang	China
6	Renmin Hospital of Wuhan University	China	30	The First People’s Hospital of Yichang	China
7	Tianyou Hospital, Wuhan University of Science and Technology	China	31	Affiliated Renhe Hospital of China Three Gorges University	China
8	Puren Hospital, Wuhan University of Science and Technology	China	32	Shiyan Renmin Hospital	China
9	The Third People’s Hospitals of Hubei province	China	33	Taihe Hospital	China
10	Hubei Province Hospital of Traditional Integrated Chinese and Western Medcine	China	34	Xiangyang No.1 People’s Hospital	China
11	The Affiliated Hospital of Hubei Provincial Government	China	35	Xiangyang Central Hospital	China
12	Hubei Cancer Hospital	China	36	Xiangyang Hospital of Traditional Chinese Medicine	China
13	Hubei Provincial Hospital of TCM Affiliated to Hubei University of Chinese Medicine	China	37	The Central Hospital of Xiaogan	China
14	Wuhan Hospital of Traditional Chinese and Western Medicine	China	38	Xianning Central Hospital	China
15	The Central Hospital of Wuhan	China	39	Jingmen NO.1 People’s Hospital	China
16	Wuhan Third Hospital	China	40	Jingmen NO.2 People’s Hospital	China
17	Puai Hospital, Tongji Medical College, Huazhong University of Science and Technology	China	41	Xiantao First People’s Hospital	China
18	Fifth Hospital in Wuhan	China	42	Jingzhou First People’s Hospital	China
19	The Sixth Hospital of Wuhan	China	43	Jingzhou Central Hospital	China
20	Wuhan No.7 Hospital	China	44	The Central Hospital of Enshi Tujia and Miao Autonomous Prefecture	China
21	The Eighth Hospital of Wuhan	China	45	Enshi Center hospital	China
22	Wuhan No.9 Hospital	China	46	Huangshi Central Hospital	China
23	Wuhan Red Cross Hospital	China	47	The Fifth Hospital of Huangshi	China
24	Wuhan Hanyang Hospital	China	48	Huanggang Central hospital	China

### Eligibility criteria {10}

Patients recruited from the gastroenterology department in hospitals will be prospectively recruited prior to treatment of GERD. Eligibility to participate will be assessed as follows. GERD patients include EE and NERD. EE is defined by the Los Angeles (LA) classification (grade A/B/C/D). Patients with typical symptoms (heartburn or regurgitation) but without EE are defined as NERD. All patients are eligible for the allocated intervention if they meet each of the following inclusion criteria:

1.  ≥ 18 years old
2. Patients having completed upper gastrointestinal endoscopy in the past year
3. Patients with typical (heartburn or regurgitation) or untypical symptoms (retrosternal burning pain or discomfort) for at least 1 month
4. NERD patients or EE patients (LA grade A) with GERD Questionnaire (GERD-Q) ≥ 8, or EE patients (LA grade B/C/D)
5. Patients are willing to adhere to the allocated regimens and the follow-up procedure.

Patients are to be excluded from the allocated intervention if, at the time of presentation, they meet any of the following criteria:

1. Patients taking gastric acid-inhibited drugs (PPIs, H_2_RA, and other PCABs) and prokinetic drugs in the last week
2. Patients receiving atazanavir, rilpivirine and nelfinavir
3. Patients with acute peptic ulcer, previous gastric or esophageal surgery history
4. Patients with diseases of the hepatobiliary system such as jaundice, cholelithiasis, and hypohepatia
5. Patients with serious diseases such as hepatic failure, renal insufficiency, and cancers
6. Pregnant and lactating women
7. Patients with communication and mental disorders

### Who will take informed consent? {26a}

Informed consent will be signed by two sides (blinded physicians and patients) at the outpatient clinic before screening. Patients will read the printed consent and have sufficient time for patients to fully consider whether to participate. The blinded physicians will explain the research process and answer questions from the patients during the consent duration.

### Additional consent provisions for collection and use of participant data and biological specimens {26b}

Not applicable. No samples will be collected.

### Interventions

#### Explanation for the choice of comparators {6b}

In many countries, vonoprazan 20 mg is the approved dose to treat GERD [5, 20]. In China, the recommended dose is vonoprazan 20 mg once daily for 4 weeks. Vonoprazan was well tolerated, and no drug-related severe adverse effects were reported in Asian patients with vonoprazan [5]. To sum up, the choice of comparator, vonoprazan 20 mg once daily, is justified.

#### Intervention description {11a}

The treatment regimens include (1) A regimen: vonoprazan 20 mg p.o. after breakfast; (2) B regimen: vonoprazan 20 mg p.o. after dinner; and (3) C regimen: vonoprazan 20 mg p.o. after breakfast and after dinner. Patients should take the drug before 10 AM or 10 PM, respectively.

#### Criteria for discontinuing or modifying allocated interventions {11b}

Patients will be grouped into three vonoprazan regimens for the compulsory randomization cluster. For the preference cluster, patients can choose to enter each group based on their preference for managing GERD, whereas the patients will be randomized if they have no clear preference. Suppose patients discontinued or modified the allocated interventions. In that case, their data will not be assessed, and their symptom e-diary and follow-up visits post-discontinuation and modifying interventions will not be collected. However, the adverse effects will continue to be tracked by researchers.

The researchers may terminate the interventions (1) if patients are with poor adherence; (2) if clinical adverse effects occur, patients could no longer obtain the best interest in this trial; (3) if disease progression worsens; and (4) if patients meet exclusion criteria. If a study-related harm occurs, the researchers will handle related harm according to standard medical procedures.

#### Strategies to improve adherence to interventions {11c}

Firstly, preferred interventions have previously been shown to promote treatment adherence. Besides, we will regularly check the symptom e-diary online remotely. If missing data is noted, we will remind the patients by phone to continue the e-diary.

#### Relevant concomitant care permitted or prohibited during the trial {11d}

Patients can maintain regular treatment for comorbidities during the trial, such as antihypertensive and glucose-lowering drugs. However, all patients will be prohibited from receiving other gastric acid-inhibited drugs (PPIs, H_2_RA, and other PCABs) and prokinetic drugs.

#### Provisions for post-trial care {30}

If participants suffer any harm from the trial, they will be treated according to the standard medical procedures.

### Outcomes {12}

The primary outcome is the symptom relief rate of all patients after 4-week therapy. The symptom relief is defined as the frequency of symptoms in the fourth week, which was 75% improved from the baseline.

Secondary outcomes are as follows: (1) the severity of symptoms in the fourth week. The severity of symptoms will be assessed by the Visual Analogue Scale (VAS) score from 0 to 10, and the severity of the fourth week will be the average score of 7 days of the fourth week; (2) the healing rate of EE patients after 4 weeks of therapy. The healing is defined as no erosion of the esophageal mucosa in the endoscopic reexamination; (3) the compliance of the 4-week therapy. The compliance will be measured by the medication days completed ratios during 4 weeks; (4) the recurrent symptoms and the frequency of self-conscious doctor visits of GERD at the sixth-month visit.

### Participant timeline {13}

Patients will attend a baseline visit, a 4-week treatment visit, and a 6-month follow-up visit online. At the baseline visit, we will collect health information about demographics, comorbidities, medication history, endoscopic diagnosis, symptoms of GERD (frequency and severity), quality of life (by Health-Related Quality-of-Life, GERD-HRQL), sleep quality (by Pittsburgh sleep quality index, PSQI), and mental health (by 7-tiem Generalized Anxiety Disorder Scale, GAD-7; Patient Health Questionnaire-9, PHQ-9). The symptoms of GERD were according to GERD-Q, Reflux Disease Questionnaires (RDQ), and Reflux Symptom Index (RSI).

**Fig. 2:**
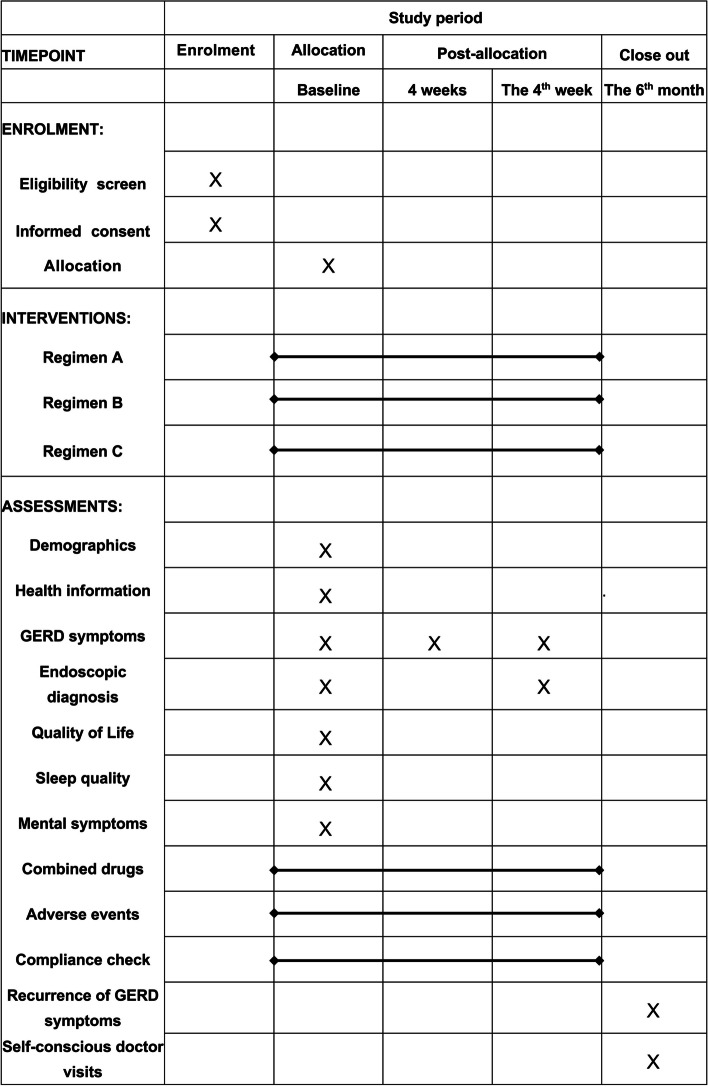
Schedule of enrolment, interventions, and assessments

During the treatment of 4 weeks, patients should record the severity and frequency of GERD-related symptoms daily in the online diary (e-diary). At the 4-week online visit, we will re-assess endoscopic diagnosis (only EE patients), symptoms of GERD, and adverse effect (AE) data. We will also record the details of patient compliance in the 4-week e-diary and visit. At the 6-month visit, we will record the recurrent symptoms of GERD and the details of self-conscious doctor visits of GERD (Fig. 2).

### Sample size {14}

The sample size was calculated by PASS 15. We will do a non-inferiority test between control group A (A regimen) and test group B (B regimen). We estimate the response rate of vonoprazan in test group B and control group A was 70% [5, 12]. The sample size of each cluster is 50(M). The calculation is based on an intra-cluster correlation of 0.02. The non-inferiority response rate is 60%. For 80% power at an alpha level of 0.025 with a one-side test, the required number of control and test groups are both 14.

The superiority test will be done between control group A (A regimen) and test group C (C regimen). We estimate the response rate of vonoprazan in test group B and control group C was 70% and 78%, respectively [5, 12], and the superiority response rate is 80%. The intra-cluster correlation is 0.02. We estimate the sample size of each cluster is 50(M), and control group A has 15 clusters. For 80% power at an alpha level of 0.025 with a one-side test, the required number of test group C was 16.

We will implement the three interventions with the patient allocation ratio of 1:1:1. Therefore, the number of clusters in each group will be 16(K), and the study’s sample size is 16 × 3 × 50 = 2400. With conservative estimates of a 16.7% dropout rate among patients at each site, at least 2880 participants (60 participants per site) should be included in this study.

### Recruitment {15}

Recruitment posters at the gastroenterology clinic and website recruitment advertisements will be used.

## Assignment of interventions: allocation

### Sequence generation {16a}

The number of hospitals was computer-generated random numbers, and cluster randomization will be used to ensure that each site will have a balanced distribution among interventions. The crossover-cluster design was according to the calendar month in each site.

### Concealment mechanism {16b}

The qualified researchers, independent of the trial implementation, are responsible for generating and maintaining the database’s randomization list. Central randomization will be used to minimize the selection bias.

### Implementation {16c}

The independent researchers will generate the allocation sequence. Blinded physicians at the clinic will screen and enroll the eligible patients. The assignment schedule is unpredictable and locked away from the enrollment physicians. So the enrolled patients will obtain the assigned interventions from another unblinded prescription physician.

## Assignment of interventions: blinding

### Who will be blinded {17a}

Patients will not be blinded. Besides, physicians who participated in our study included two categories. Enrollment physicians (blinded) will be responsible for enrollment, data collection, follow-up visits, and outcome evaluation. Prescribing physicians (unblinded) will be specialized in prescribing the drugs according to the randomization.

### Procedure for unblinding if needed {17b}

Patients and prescribing physicians cannot be blinded to the intervention. However, physicians related to data management and statisticians will be blinded to avoid information bias. For the independent statisticians, the names and clusters will be replaced by the unique randomization numbers, and the interventions will also be maintained by specific group names (regimen A/B/C). Plain language summaries and consent forms will use neutral wording on the effectiveness of different regimens to maintain equipoise and to avoid expectancy effects among unblinded patients.

## Data collection and management

### Plans for assessment and collection of outcomes {18a}

The consent will be obtained before any assessment, including the patient demographic. All patients will be approached for enrolment for data collection within the trial by site study physicians. The study physician will be responsible for patient recruitment and open-label treatment at each site. A clinical coordinator will regularly remind patients of online visits in time. Brief demographic details of all patients approached but not enrolled and the reason for nonenrolment will be recorded on a screening and enrolment log to determine selection bias. Before starting the trial, all researchers will be trained on the standard operating procedure, and ePRO database system use and complete simulated baseline and follow-up visits.

We will collect data by electronic Patient-Reported Outcomes (ePRO) data, health information from the hospital’s electronic medical record system, and external data. ePRO is self-reported data applicable to the primary study endpoint of this study—subjective symptom improvement of patients. ePRO data will be uploaded under the guidance of the clinical coordinator at any visits and daily records by patients. Each hospital’s structured and standardized electronic medical record system will provide comprehensive health information, including demographic and clinical characteristics, laboratory examinations, and endoscopy reports. In addition, medical reports provided by other suppliers are defined as external data.

Except in the rare case of patient preference or equipment failure, when participants complete a paper version, all patients will complete the questionnaires online. The Wenjuanxing website building in the WeChat Official Account is planned as the data collection platform for this study.

### Plans to promote participant retention and complete follow-up {18b}

We will remind participants to complete the follow-up by phone call or message. The last two visits will be scheduled for the fourth week ± 3 days and the sixth month ± 14 days, respectively. If a patient does not complete the visit within the scheduled period for any reason, we will not collect follow-up data for that period, and the data for that visit will be considered missing. Patients will drop out of the study if they fail to complete the 2 weeks of e-diary of symptoms.

## Data management {19}

The data management plan will be formulated under the guidance of authoritative experts following the technical guidelines for Chinese clinical trial data management [34]. Data collected from different hospitals should be stored in the database. The database will be set up and maintained using Microsoft Excel 2019 to ensure data quality. Microsoft Excel is a system for safely inputting, storing, and retrieving research data. The database is accessible only via password-protected computers by qualified researchers. These computers are located in dedicated, locked key-entry research offices accessible only by researchers who have signed a study-specific confidentiality agreement. We will store data on another independent computer that is backed up daily. The final data set will be backed up on Compact Disc Read—Only Memory (CD-ROM). If the database is irreparably damaged, the latest backup data set will be used to restore the database and input the following supplementary data.

The data manager shall check all collected data promptly and initiate the data queries of the problems found, including manual checks and computer program checks. For example, specific functions in Excel can test for null and abnormal values. Before the clinical trial database is locked, the physician, the data manager, and the statistical analyst shall solve the problems with blinded access. The data editing permissions for the database should be withdrawn once the database locking has been approved in writing, and the withdrawal date should be documented.

### Confidentiality {27}

This trial will follow the Personal Information Protection Law of China. The database will be kept confidential and anonymous. It records the specific codes of patients rather than their full names. In presentations or publications arising from this study, information will be provided in such a way that patients cannot be identified. No study details can be disclosed to unauthorized third parties without prior approval.

### Plans for collection, laboratory evaluation, and storage of biological specimens for genetic or molecular analysis in this trial/future use {33}

Not applicable. No specimens will be collected.

## Statistical methods

### Statistical methods for primary and secondary outcomes {20a}

We will analyze patients in the treatment group to which they are allocated according to the intention-to-treat (ITT) principle. All analyses will be undertaken at the patient level. The hypothesis will be examined by two contrasts (between A and B regimens and between A and C regimens) evaluating the effectiveness of vonoprazan treatment. We will present numerical variables as means or medians (interquartile range) and categorical variables as counts and percentages (with 95% confidence interval). We will use a significance level of 0.05.

### Interim analyses {21b}

Electronic data will be performed at any interim analysis only by blinded data analysts, and the trial steering committee will have access to these interim results and make the final decision to terminate the trial.

### Methods for additional analyses (e.g., subgroup analyses) {20b}

Data analysis will be conducted separately for the randomized and nonrandomized sets. For the randomized set, continuous variables will be compared by *t*-tests, and categorical variables will be compared by chi-square tests. These analyses will not be adjusted for covariates unless factors are significantly unbalanced at baseline. We will utilize linear-mixed models or logistic regression models, depending on the data distribution for the nonrandomized set, to adjust potential confounders. The between-group difference will be measured by the mean differences for continuous outcomes (i.e., symptom scores) and the risk ratios for binary outcomes (i.e., symptom relief rate).

Subgroup analyses will also be conducted to investigate the effectiveness of the interventions in different subtypes based on our clinical experience (i.e., Time of symptom occurrence, Grade of EE, comorbidities). In addition, subgroup analyses comparing patients who underwent compulsory and voluntary randomization will be carried out to validate the impact of patient preference on the effects.

Descriptive statistics will be provided for safety data. The number of patients reporting any AEs or suppose serious adverse events (SAEs) and the occurrence of specific SAEs will be tabulated.

### Methods in analysis to handle protocol non-adherence and any statistical methods to handle missing data {20c}

We will examine the missing data mechanisms for handling missing data, including missing entirely at random, missing at random, and missing not at random. The proportion of valid reasons for missing data will determine the need to impute missing values. Appropriate methods, such as the multiple imputation and pattern mixture models, are used if imputation is necessary. A range of sensitivity analyses to examine the robustness of the primary model results will be performed to explore the impact of the results of different missing data techniques under the same and different missingness assumptions.

### Plans to give access to the full protocol, participant-level data, and statistical code {31c}

The plans of this study are available on the clinical trial registration website.

## Oversight and monitoring

### Composition of the coordinating center and trial steering committee {5d}

A trial steering committee (TSC) will be established for quality assurance, protocol amendments, study conduct, and outcome assessment. The TSC will be supplemented by members, including gastroenterologists, methodologists, and statisticians with strong expertise who are not otherwise affiliated with the project or its involved institutions.

The TSC will have regular meetings to supervise trial updates of all sites closely. It will have unblinded access to study data and could decide whether to continue the trial based on the safety, effectiveness, and compliance assessment results. The details of monitoring are as follows:

1. Study conduct: The principal clinical coordinator of each hospital will be responsible for reporting the trial’s progress to TSC. Recruitment and retention rates will be monitored closely to mitigate the risk of slow recruitment over the trial period.
2. Endpoint adjudication: Independent gastroenterologists were responsible for reviewing the assessment of the primary and secondary outcomes. They will make the final decision to terminate the trial.

### Composition of the data monitoring committee, its role and reporting structure {21a}

The data monitoring committee (DMC) will also be supplemented by gastroenterologists, methodologists, and statisticians who are not otherwise affiliated with the project or its involved institutions. A trial database will be set up and maintained using Microsoft Excel 2019, and the DMC will be responsible for data quality management and safety monitoring. Data monitoring will require on-site monitoring centrally. The DMC meeting will be held four times: 25%, 50%, 75%, and 100% of the patients completed the trial procedure. The monitoring will be performed according to the Technical Guidelines for Clinical Trial Data Management by the Chinese National Medical Products Administration [34].

### Adverse event reporting and harms {22}

AEs are adverse medical events that occur after patients have received the intervention drugs, which can be manifested as symptoms, diseases, or laboratory abnormalities. AEs will be reported as recommended by laws and regulations by the China Food and Drug Administration. Responsible enrolled physicians will evaluate AE occurrence periodically for causality, expectedness, and severity. To avoid bias in obtaining AE information, researchers should use the non-leading question, “How have you been feeling since your last visit?” AE reports should use medical terminology and be reported as a single diagnosis or as separate signs or symptoms. The details of AE include the occurrence time, symptoms, clinical signs, severity, duration, laboratory reports, treatment process and prognosis, and follow-up time. The combination of drug use should be recorded in detail to analyze the correlation between AEs and the study’s intervention. Records should be signed and dated by researchers.

SAEs refer to adverse medical events such as death, life-threatening, permanent, or severe disability, loss of function, or the need for hospitalization after patients received the trial drug. When SAEs occur during the study, researchers must report SAEs to the provincial administration department, the responsible clinical research site, and the Medical Ethics Committee within 24 h or no later than the second working day. The researchers should sign and date the report.

AEs and SAEs will be recorded on the data collection form and database. The participating hospitals should ensure that reporting procedures meet the legal and regulatory requirements. In addition, all SAEs will be appointed to the independent Data and Safety Monitoring Board (DSMB) for regular review.

### Frequency and plans for auditing trial conduct {23}

The DMC will assign a dedicated, qualified individual not affiliated with the study to conduct regular inspections. This progress will prove the data source and ensure data reliability.

### Plans for communicating necessary protocol amendments to relevant parties (e.g., trial participants, ethical committees) {25}

If the protocol changes during the implementation of the study, researchers will communicate the vital protocol modifications (e.g., changes to eligibility criteria, outcomes, and analyses). The TSC will have regular meetings to supervise the study’s amendments to relevant parties closely.

## Dissemination plans {31a}

We will disseminate the study’s results widely through conference presentations or publications. The publication’s authors should be the directly related researchers of this study.

## Discussion

Without effective treatment, severe complications of GERD, such as esophageal stricture, ulceration, or Barrett’s esophagus, may develop [35]. Vonoprazan offers rapid, potent, and long-term maintenance acid-inhibitory effects [36, 37]. Studies have reported that vonoprazan 20 mg is an appropriate dosage in Asia for the healing of EE, but there are still several unsolved problems for the resolution of GERD.

Firstly, vonoprazan was only approved to treat EE in China, but it is still unknown for the resolution of symptoms of Chinese patients with NERD. Secondly, there is no direct clinical evidence on different dosages of vonoprazan for GERD patients. Thirdly, different dosing times could be a predictive factor of the clinical effectiveness of vonoprazan. In summary, our proposed pragmatic RCT will assess different regimens of vonoprazan implemented with GERD patients.

We have several strengths in study design, as follows. Pragmatic effectiveness trial design could increase trial efficiency, facilitation of enrollment of broad cohorts, and ability to generate evidence relevant to the actual practice environment [38]. Different healthcare settings will support the generalizability of results, and a cluster randomized design with the interventions applied as part of usual clinical procedures was chosen to avoid contamination and maximize adherence, reliability, and generalizability of the results [39, 40]. A crossover component is added to provide all hospital sites with a standardized change of regimens. This allows us to control for confounding factors associated with differences in the organization and levels of background clinical procedures across hospitals [41, 42]. The current meta-analysis showed that matching patients to preferred interventions has previously been shown to promote outcomes such as treatment adherence and pain relief [43], so patient preference may influence the effectiveness of vonoprazan for GERD in real-world situations. This study’s evidence from nonrandom samples may also have better external validity. Besides, this design will help to recruit more participants and enhance the adherence of participants.

There are still some limitations. Patients without ineffective treatment may not easily adhere to the fourth-week and sixth-month follow-up visits. The physician’s explanation of the effectiveness of treatment during the informing process may help to overcome this. In addition, the baseline evaluation of symptoms may be affected by recall bias, which is inevitable when recalling symptoms of GERD. However, the bias would be the same for each group.

In summary, this will be the first pragmatic RCT to evaluate the effectiveness of different regimens of vonoprazan for GERD and will produce real-world evidence. We hope our results will inform policy decisions by providing reliable evidence about the clinical effectiveness of GERD treatment.

## Trial status

The protocol version number and date: Version 2; February 18, 2023,

The date recruitment began: March 31, 2023

The approximate date when recruitment will be completed: February 28, 2025

### Supplementary Information

**Additional file 1.**

**Additional file 2.**

**Additional file 3.**

**Additional file 4.**

## Data Availability

Only researchers in this study have access to the final trial dataset, which was unavailable to the public.

## Abbreviations

AEs: Adverse Events
CD-ROM: Compact Disc Read - Only Memory
CONSORT: Consolidated Standards of Reporting Trials
DMC: Data Monitoring committee
DSMB: Data and Safety Monitoring Board
ePRO: Electronic Patient-Reported Outcomes
e-diary: Electronic diary
EE: Erosive Esophagitis
GAD-7: 7-Tiem Generalized Anxiety Disorder Scale
GERD: Gastroesophageal Reflux Disease
GERD -Q: GERD-Questionnaires
GERD-HRQL: GERD-Health-Related Quality of Life
H_2_RA: H_2_ Receptor Antagonist
ITT: Intention-to-treat
LA: Los Angeles
NERD: Non-erosive Reflux Disease
p.o.: Take orally
PCAB: Potassium-competitive Acid Blocker
PHQ-9: Patient Health Questionnaire-9
PPIs: Proton-pump Inhibitors
PSQI: Pittsburgh Sleep Quality Index
RCT,: Randomized Controlled Trial
RDQ: Reflux Disease Questionnaires
RSI: Reflux Symptom Index
SAEs: Serious Adverse Events
SPIRIT: Standard Protocol Items: Recommendations for Interventional Trials
t1/2,: Plasma half-life
TSC: Trial steering committee
VAS: Visual Analogue Scale

## References

[1] Fass R, Boeckxstaens GE, El-Serag H, Rosen R, Sifrim D, Vaezi MF (2021). Gastro-oesophageal reflux disease. Nat Rev Dis Primers.

[2] Eusebi LH, Ratnakumaran R, Yuan Y, Solaymani-Dodaran M, Bazzoli F, Ford AC (2018). Global prevalence of, and risk factors for, gastro-oesophageal reflux symptoms: a meta-analysis. Gut.

[3] Miwa H, Uedo N, Watari J, Mori Y, Sakurai Y, Takanami Y, Nishimura A, Tatsumi T, Sakaki N (2017). Randomised clinical trial: efficacy and safety of vonoprazan vs. lansoprazole in patients with gastric or duodenal ulcers - results from two phase 3, non-inferiority randomized controlled trials. Aliment Pharmacol Ther.

[4] Murakami K, Sakurai Y, Shiino M, Funao N, Nishimura A, Asaka M (2016). Vonoprazan, a novel potassium-competitive acid blocker, as a component of first-line and second-line triple therapy for Helicobacter pylori eradication: a phase III, randomized, double-blind study. Gut.

[5] Xiao Y, Zhang S, Dai N, Fei G, Goh KL, Chun HJ, Sheu BS, Chong CF, Funao N, Zhou W, Chen M (2020). Phase III, randomised, double-blind, multicentre study to evaluate the efficacy and safety of vonoprazan compared with lansoprazole in Asian patients with erosive oesophagitis. Gut.

[6] Inatomi N, Matsukawa J, Sakurai Y, Otake K (2016). Potassium-competitive acid blockers: advanced therapeutic option for acid-related diseases. Pharmacol Ther.

[7] Sugano K (2018). Vonoprazan fumarate, a novel potassium-competitive acid blocker, in the management of gastroesophageal reflux disease: safety and clinical evidence to date. Therap Adv Gastroenterol.

[8] Hori Y, Matsukawa J, Takeuchi T, Nishida H, Kajino M, Inatomi N (2011). A study comparing the antisecretory effect of TAK-438, a novel potassium-competitive acid blocker, with lansoprazole in animals. J Pharmacol Exp Ther.

[9] Ashida K, Iwakiri K, Hiramatsu N, Sakurai Y, Hori T, Kudou K, Nishimura A, Umegaki E (2018). Maintenance for healed erosive esophagitis: phase III comparison of vonoprazan with lansoprazole. World J Gastroenterol.

[10] Ashida K, Sakurai Y, Hori T, Kudou K, Nishimura A, Hiramatsu N, Umegaki E, Iwakiri K (2016). Randomised clinical trial: vonoprazan, a novel potassium-competitive acid blocker, vs. lansoprazole for the healing of erosive oesophagitis. Aliment Pharmacol Ther.

[11] Ashida K, Sakurai Y, Nishimura A, Kudou K, Hiramatsu N, Umegaki E, Iwakiri K, Chiba T (2015). Randomised clinical trial: a dose-ranging study of vonoprazan, a novel potassium-competitive acid blocker, vs. lansoprazole for the treatment of erosive oesophagitis. Aliment Pharmacol Ther.

[12] Oshima T, Arai E, Taki M, Kondo T, Tomita T, Fukui H, Watari J, Miwa H (2019). Randomised clinical trial: vonoprazan versus lansoprazole for the initial relief of heartburn in patients with erosive oesophagitis. Aliment Pharmacol Ther.

[13] Hori Y, Imanishi A, Matsukawa J, Tsukimi Y, Nishida H, Arikawa Y, Hirase K, Kajino M, Inatomi N (2010). 1-[5-(2-Fluorophenyl)-1-(pyridin-3-ylsulfonyl)-1H-pyrrol-3-yl]-N-methylmethanamine monofumarate (TAK-438), a novel and potent potassium-competitive acid blocker for the treatment of acid-related diseases. J Pharmacol Exp Ther.

[14] Kagami T, Sahara S, Ichikawa H, Uotani T, Yamade M, Sugimoto M, Hamaya Y, Iwaizumi M, Osawa S, Sugimoto K, Miyajima H, Furuta T (2016). Potent acid inhibition by vonoprazan in comparison with esomeprazole, with reference to CYP2C19 genotype. Aliment Pharmacol Ther.

[15] Fass R, Fennerty MB, Vakil N (2001). Nonerosive reflux disease–current concepts and dilemmas. Am J Gastroenterol.

[16] Lee ES, Kim N, Lee SH, Park YS, Kim JW, Jeong SH, Lee DH, Jung HC, Song IS (2009). Comparison of risk factors and clinical responses to proton pump inhibitors in patients with erosive oesophagitis and non-erosive reflux disease. Aliment Pharmacol Ther.

[17] Miwa H, Sasaki M, Furuta T, Koike T, Habu Y, Ito M, Fujiwara Y, Wada T, Nagahara A, Hongo M, Chiba T, Kinoshita Y, ACID-RELATED SYMPTOM (ARS) RESEARCH GROUP (2007). Efficacy of rabeprazole on heartburn symptom resolution in patients with non-erosive and erosive gastro-oesophageal reflux disease: a multicenter study from Japan. Aliment Pharmacol Ther.

[18] Fass R (2007). Erosive esophagitis and nonerosive reflux disease (NERD): comparison of epidemiologic, physiologic, and therapeutic characteristics. J Clin Gastroenterol.

[19] Kinoshita Y, Sakurai Y, Shiino M, Kudou K, Nishimura A, Miyagi T, Iwakiri K, Umegaki E, Ashida K (2016). Evaluation of the efficacy and safety of vonoprazan in patients with nonerosive gastroesophageal reflux disease: a phase III, randomized, double-blind, placebo-controlled, multicenter study. Curr Ther Res Clin Exp.

[20] https://www.takeda.com/newsroom/newsreleases/2015/takecab-now-available-for-the-treatment-of-acid-related-diseases-in-japan/

[21] Sakurai Y, Nishimura A, Kennedy G, Hibberd M, Jenkins R, Okamoto H, Yoneyama T, Jenkins H, Ashida K, Irie S, Täubel J (2015). Safety, tolerability, pharmacokinetics, and pharmacodynamics of single rising TAK-438 (vonoprazan) doses in healthy male Japanese/non-Japanese subjects. Clin Transl Gastroenterol.

[22] Jenkins H, Sakurai Y, Nishimura A, Okamoto H, Hibberd M, Jenkins R, Yoneyama T, Ashida K, Ogama Y, Warrington S (2015). Randomised clinical trial: safety, tolerability, pharmacokinetics and pharmacodynamics of repeated doses of TAK-438 (vonoprazan), a novel potassium-competitive acid blocker, in healthy male subjects. Aliment Pharmacol Ther.

[23] Hunt RH (1999). Importance of pH control in the management of GERD. Arch Intern Med.

[24] Oshima T, Miwa H (2016). Gastrointestinal mucosal barrier function and diseases. J Gastroenterol.

[25] Hunt RH (1995). The relationship between the control of pH and healing and symptom relief in gastro-oesophageal reflux disease. Aliment Pharmacol Ther.

[26] Shaker R, Castell DO, Schoenfeld PS, Spechler SJ (2003). Nighttime heartburn is an under-appreciated clinical problem that impacts sleep and daytime function: the results of a Gallup survey conducted on behalf of the American Gastroenterological Association. Am J Gastroenterol.

[27] Farup C, Kleinman L, Sloan S, Ganoczy D, Chee E, Lee C, Revicki D (2001). The impact of nocturnal symptoms associated with gastroesophageal reflux disease on health-related quality of life. Arch Intern Med.

[28] Yacyshyn BR, Thomson AB (2002). The clinical importance of proton pump inhibitor pharmacokinetics. Digestion.

[29] Nehra AK, Alexander JA, Loftus CG, Nehra V (2018). Proton pump inhibitors: review of emerging concerns. Mayo Clin Proc.

[30] Vaughn B, Rotolo S, Roth H. Circadian rhythm and sleep influences on digestive physiology and disorders. ChronoPhysiol Ther. 2014:67.

[31] Yang E, Kim S, Kim B, Kim B, Kim Y, Park SS, Song GS, Yu KS, Jang IJ, Lee S (2022). Night-time gastric acid suppression by tegoprazan compared to vonoprazan or esomeprazole. Br J Clin Pharmacol.

[32] Schulz KF, Altman DG, Moher D, CONSORT Group (2010). CONSORT 2010 statement: updated guidelines for reporting parallel group randomized trials. Ann Intern Med.

[33] Chan AW, Tetzlaff JM, Altman DG, Laupacis A, Gøtzsche PC, Krleža-Jerić K, Hróbjartsson A, Mann H, Dickersin K, Berlin JA, Doré CJ, Parulekar WR, Summerskill WS, Groves T, Schulz KF, Sox HC, Rockhold FW, Rennie D, Moher D (2013). SPIRIT 2013 statement: defining standard protocol items for clinical trials. Ann Intern Med.

[34] Available at: https://www.nmpa.gov.cn/directory/web/nmpa/xxgk/ggtg/qtggtg/20160729183801891.html.

[35] Triantos C, Koukias N, Karamanolis G, Thomopoulos K (2015). Changes in the esophageal mucosa of patients with non erosive reflux disease: How far have we gone?. World J Gastroenterol.

[36] Miwa H, Igarashi A, Teng L, Uda A, Deguchi H, Tango T (2019). Systematic review with network meta-analysis: indirect comparison of the efficacy of vonoprazan and proton-pump inhibitors for maintenance treatment of gastroesophageal reflux disease. J Gastroenterol.

[37] Cheng Y, Liu J, Tan X, Dai Y, Xie C, Li X, Lu Q, Kou F, Jiang H, Li J (2021). Direct comparison of the efficacy and safety of vonoprazan versus proton-pump inhibitors for gastroesophageal reflux disease: a systematic review and meta-analysis. Dig Dis Sci.

[38] Rice TW, Kripalani S, Lindsell CJ (2020). Proton pump inhibitors vs histamine-2 receptor blockers for stress ulcer prophylaxis in critically ill patients: issues of interpretability in pragmatic trials. JAMA.

[39] Benzies KM, Shah V, Aziz K, Isaranuwatchai W, Palacio-Derflingher L, Scotland J, Larocque J, Mrklas K, Suter E, Naugler C, Stelfox HT, Chari R, Lodha A, Alberta FICare Level II NICU Study Team (2017). Family Integrated Care (FICare) in Level II Neonatal Intensive Care Units: study protocol for a cluster randomized controlled trial. Trials.

[40] Sessler DI, Myles PS (2020). Novel clinical trial designs to improve the efficiency of research. Anesthesiology.

[41] Muñoz-Venturelli P, Arima H, Lavados P, Brunser A, Peng B, Cui L, Song L, Billot L, Boaden E, Hackett ML, Heritier S, Jan S, Middleton S, Olavarría VV, Lim JY, Lindley RI, Heeley E, Robinson T, Pontes-Neto O, Natsagdorj L, Lin RT, Watkins C, Anderson CS, HeadPoST Collaborative Investigators (2015). Head Position in Stroke Trial (HeadPoST)–sitting-up vs lying-flat positioning of patients with acute stroke: study protocol for a cluster randomised controlled trial. Trials.

[42] Self WH, Evans CS, Jenkins CA, Brown RM, Casey JD, Collins SP, Coston TD, Felbinger M, Flemmons LN, Hellervik SM, Lindsell CJ, Liu D, McCoin NS, Niswender KD, Slovis CM, Stollings JL, Wang L, Rice TW, Semler MW, Pragmatic Critical Care Research Group (2020). Clinical effects of balanced crystalloids vs saline in adults with diabetic ketoacidosis: a subgroup analysis of cluster randomized clinical trials. JAMA Netw Open.

[43] Delevry D, Le QA (2019). Effect of treatment preference in randomized controlled trials: systematic review of the literature and meta-analysis. Patient.

