# Supplementary material for: Different dosages of vonoprazan for gastroesophageal reflux disease: study protocol for a pragmatic, crossover-cluster, randomized controlled trial with patient preference arms
Source: Trials. 2023 Dec 1;24:778. doi: 10.1186/s13063-023-07760-9 (PMC10691065; doi:10.1186/s13063-023-07760-9)
Supplement: Supplementary file 1 — Additional file 1. [file 13063_2023_7760_MOESM1_ESM.pdf]

核准日期：2020 年 4 月 7 日

修改日期：2020 年 7 月 10 日

修改日期：2020 年 9 月 29 日

富马酸伏诺拉生片说明书

请仔细阅读说明书并在医师指导下使用。

【药品名称】

通用名称：富马酸伏诺拉生片

商品名称：沃克（Vocinti）

英文名称：Vonoprazan Fumarate Tablets

汉语拼音：Fumasuan Funuolasheng Pian

【成份】

本品主要成分为富马酸伏诺拉生。

化学名称：1-[5-(2-氟苯基)-1-(吡啶-3-磺酰基)-1*H*-吡咯-3-基]-*N*-甲基甲胺单富马酸盐

化学结构式：

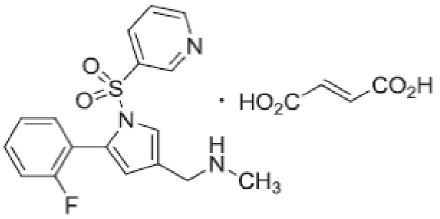

分子式：$C_{17}H_{16}FN_3O_2S \cdot C_4H_4O_4$

分子量：461.46

【性状】

10mg（按 $C_{17}H_{16}FN_3O_2S$ 计）：本品为浅黄色薄膜衣片，除去包衣后显白色。

20mg（按 $C_{17}H_{16}FN_3O_2S$ 计）：本品为浅红色薄膜衣片，除去包衣后显白色。

【适应症】

反流性食管炎。

【规格】按 $C_{17}H_{16}FN_3O_2S$ 计（1）10mg；（2）20mg

【用法用量】

口服。成人每日 1 次，每次 20mg。大部分患者通常 4 周可获益，如果疗效不佳，疗程最多可延长至 8 周。

【不良反应】

临床试验

下表列出了本品国内外临床试验期间报告的不良反应。发生频率分类使用以下惯例并依据国际医学科学组织委员会（CIOMS）指南：十分常见（≥1/10）；常见（≥1/100 至＜1/10）；偶见（≥1/1,000 至＜1/100）；罕见（≥1/10,000 至﹤ 1/1,000）；十分罕见（＜1/10,000）；不详（无法根据可用数据进行估计）。

表 1 伏诺拉生临床试验期间的不良反应

发生频率/系统器官分类	十分常见	常见	偶见	罕见
胃肠道疾病	--	腹泻<br>便秘	恶心<br>腹胀	--
检查值	--	--	γ-谷氨酰转移酶升高<br>天门冬氨基转移酶（AST）升高<br>丙氨酸氨基转移酶（ALT）升高<br>碱性磷酸酶（ALP）升高<br>乳酸脱氢酶（LDH）升高	--
神经系统疾病	--	--	头痛	--
皮肤和皮下组织疾病			皮疹	
其他	--	--	水肿<br>嗜酸性粒细胞增多	--

上市后经验

以下是在上市后观察到的但上文未包括的不良反应，频率未知。

免疫系统疾病：药物超敏反应（包括过敏性休克）、药物性皮炎、荨麻疹。

肝胆系统疾病：肝毒性、黄疸。

皮肤和皮下组织疾病：多形性红斑、史蒂文斯-约翰逊综合征、中毒性表皮坏死松解症。

【禁忌】

1. 对本品中任何成份过敏的患者禁用。
2. 正在接受阿扎那韦或利匹韦林治疗的患者禁用（参见【药物相互作用】）。

【注意事项】

1. 肝毒性：临床试验中已经报告了肝功能异常（包括肝损伤）。上市后也已收到了此类报告，其中许多发生在治疗开始后不久。应进行密切观察，如有肝功能异常证据或出现提示肝功能不

全的体征或症状，应采取包括停药在内的适当措施。

2. 伏诺拉生会导致胃内 pH 升高，因此不建议本品与吸收依赖于胃内 pH 的药物同服（参见【禁忌】、【药物相互作用】）。

3. 服用本品有可能掩盖胃恶性肿瘤的症状，开始使用本品前应先排除恶性肿瘤的可能。

4. 多项国外开展的观察性研究（主要涉及住院患者）报告在接受质子泵抑制剂治疗的患者中，艰难梭菌所引起的胃肠道感染风险增加。伪膜性结肠炎可能是根除幽门螺旋杆菌时合并使用了抗生素。如果出现异常疼痛或频繁腹泻，应采取包括停药在内的适当措施。

5. 国外开展的几项观察性研究报道，质子泵抑制剂治疗期间骨质疏松相关性髋关节、腕关节或脊柱骨折的风险增加。接受高剂量或长期（≥1 年）治疗的患者骨折风险增加更为明显。

6. 治疗时应密切观察疾病进程，并根据疾病情况使用最低必要治疗剂量。

7. 肾脏疾病患者和肝脏疾病患者慎用伏诺拉生，因为伏诺拉生的代谢和排泄可能会延迟，从而导致血液中伏诺拉生浓度升高。

8. 已有研究报道，长期给予本品期间曾观察到良性胃息肉。

【孕妇及哺乳期妇女用药】

妊娠期

迄今为止尚未在妊娠期受试者中进行评价伏诺拉生的临床研究。在一项大鼠毒理学研究中，以暴露量超过伏诺拉生最大临床剂量（40 mg/天）暴露量（AUC）约 28 倍时观察到胚胎-胎儿毒性。

注意，除非认为预期的治疗获益超过任何可能的风险，否则妊娠或可能妊娠的患者不应服用伏诺拉生。

哺乳期

迄今为止尚未在哺乳期受试者中进行评价伏诺拉生的临床研究。尚不清楚伏诺拉生是否排泄到人乳汁中。在动物研究中已经证明伏诺拉生可排泄到乳汁中。

建议在哺乳期避免服用伏诺拉生，必须给药时，应首先停止哺乳。

【儿童用药】

未进行该项试验且无可靠参考文献。

【老年用药】

由于老年患者的整体生理机能（如肝肾功能）下降，因此，老年患者应慎用本品。

【药物相互作用】

伏诺拉生会导致胃内 pH 升高，提示对于胃内 pH 是口服生物利用度重要决定因素的药物，

伏诺拉生可能影响其吸收。伏诺拉生不应与阿扎那韦、利匹韦林同服，应谨慎与奈非那韦、伊曲康唑、酪氨酸激酶抑制剂（吉非替尼、尼洛替尼、厄洛替尼）同服，因为这些药物的作用可能减弱。伏诺拉生应谨慎与地高辛、甲基地高辛同服，因为这些药物的作用可能会增强。

伏诺拉生主要通过肝脏药物代谢酶 CYP3A4 进行代谢，部分通过 CYP2B6、CYP2C19 和 CYP2D6 代谢。伏诺拉生应谨慎与 CYP3A4 抑制剂克拉霉素同服，因为伏诺拉生的血药浓度可能会升高。

【药物过量】

尚未进行本项研究，目前尚无关于过量使用本品的文献报道。

伏诺拉生不能通过血液透析除去。如果发生用药过量，应给予对症的和支持性的治疗。

【临床试验】

1. 抑制胃酸分泌作用

在健康成年男性受试者中伏诺拉生以 10 mg 或 20 mg 剂量给药 7 天后，24 小时内 pH 维持在≥4 水平的时间百分比分别为 63±9%和 83±17%。

2. 临床疗效

反流性食管炎的治疗

一项在中国大陆、马来西亚、韩国和中国台湾地区进行的 3 期多中心、随机、双盲双模拟临床试验中，反流性食管炎患者接受伏诺拉生 20mg 或兰索拉唑 30mg，每日一次，口服给药至 8 周，伏诺拉生组给药后 8 周内愈合率相对于兰索拉唑组的非劣效性得到了验证。

<table>
<tr><th>用药持续时间</th><th>伏诺拉生 20 mg</th><th>兰索拉唑 30 mg</th></tr>
<tr><td rowspan="2">4周</td><td>85.3%（203/238例）</td><td>83.5%（192/230例）</td></tr>
<tr><td colspan="2">1.8%[-4.8%, 8.4%][a)]</td></tr>
<tr><td rowspan="2">8周</td><td>92.4%（220/238例）</td><td>91.3%（210/230例）</td></tr>
<tr><td colspan="2">1.1% [-3.822%, 6.087%][a) b)]</td></tr>
</table>

括号中的数字表示愈合患者数量/评价患者数量；

a）治疗组间的差异，括号中的数字表示双侧 95%置信区间；

b）非劣效性界值 10%。

一项在日本进行的双盲对照试验中，反流性食管炎患者接受伏诺拉生 20mg 或兰索拉唑 30mg，每日一次，口服给药至 8 周，伏诺拉生组给药后 8 周内愈合率相对于兰索拉唑组的非劣效性得到了验证。此外，伏诺拉生组给药后 4 周内愈合率与兰索拉唑组给药后 8 周内愈合率差异的点估计值（双侧 95%置信区间）为 1.1% （-2.702 至 4.918%）。

<table>
<tr><td>用药持续时间</td><td>伏诺拉生 20 mg</td><td>兰索拉唑 30 mg</td></tr>
<tr><td rowspan="2">4周</td><td>96.6%（198/205例）</td><td>92.5%（184/199例）</td></tr>
<tr><td colspan="2">4.1%[-0.308%, 8.554%][a)]</td></tr>
<tr><td rowspan="2">8周</td><td>99.0%
（203/205例）</td><td>95.5%
（190/199例）</td></tr>
<tr><td colspan="2">3.5% [0.362%, 6.732%][a)]
p<0.0001[b)]</td></tr>
</table>

括号中的数字表示愈合患者数量/评价患者数量；

a）治疗组间的差异，括号中的数字表示双侧95%置信区间；

b）非劣效性界值10%。

3. 对血清胃泌素和胃粘膜内分泌细胞密度的影响

（1）伏诺拉生 10mg 或 20mg，每日一次，口服给药，血清胃泌素水平持续高于兰索拉唑15mg 组。在日本进行的临床试验中，胃溃疡或十二指肠溃疡患者给药结束后 2～8 周血清胃泌素水平恢复正常。

（2）在日本进行的反流性食管炎维持愈合临床试验中，伏诺拉生 10mg 或 20mg，每日一次，口服给药，连续 52 周，未观察到胃粘膜中的内分泌细胞密度呈明显增加趋势。

【药理毒理】

药理作用

伏诺拉生以钾离子竞争性方式可逆性抑制 H^+、K^+-ATP 酶活性，可长时间停留于胃壁细胞部位而抑制胃酸的生成，可有效抑制胃肠道上部粘膜损伤的形成。

毒理研究

遗传毒性

伏诺拉生 Ames 试验、中国仓鼠肺成纤维细胞染色体畸变试验、大鼠微核试验结果均为阴性。

生殖毒性

伏诺拉生剂量在 30、100、300mg/kg/日时，对雄性或雌性大鼠生育力及早期胚胎发育未见影响。≥100mg/kg/日剂量下亲代动物可见母体毒性，包括 300mg/kg/日剂量下 4/20 只雄性动物死亡，瞳孔散大、颤抖、俯卧位、外阴被毛脏污、活动减少、红色流涕及血泪、体重减轻及摄食量减少；100mg/kg/日剂量下瞳孔散大。对雄性和雌性动物母体毒性的 NOAEL 为

30mg/kg/天，对生殖功能和早期胚胎发育的 NOAEL 为≥300mg/kg/天。

大鼠胚胎-胎仔发育毒性试验中，伏诺拉生剂量达 100mg/kg/日未见发育毒性，300mg/kg/日剂量下可见发育毒性，包括胎仔体重降低及尾椎数减少、尾畸形、肛门狭窄、膜部室间隔缺损及锁骨下动脉起源异常等外表及内脏异常发生率增加。≥100mg/kg/日剂量下可见母体毒性，包括 300mg/kg/日剂量下 1/20 只动物死亡，瞳孔散大、颤抖、俯卧位、粪量减少、被毛污秽和流涎；≥100mg/kg 剂量母体体重增长减慢、摄食量偏低。兔胚胎-胎仔发育毒性试验中，伏诺拉生剂量在 3、10、30mg/kg/日时，对胚胎-胎仔未见影响。≥10 mg/kg/日剂量下可见母体毒性，包括粪便减少、体重或体重增加减少、摄食量减少；30mg/kg/日剂量下可见 2 只雌兔流产，1 只母兔整窝胎仔死亡。

大鼠围产期毒性试验中，剂量达 10mg/kg/日未见对母体和 F1 子代发育的明显影响。100mg/kg/日剂量下可见母体和胎仔毒性，对胎仔的影响包括体重降低、出生后第 4 天肝脏有尾状叶变色（白色和黑色）。≤100mg/kg/日剂量对 F2 代幼仔中的生存期、发育或生长均未见异常影响。伏诺拉生及其代谢产物可通过乳汁分泌，并可通过胎盘屏障。

致癌性

小鼠 2 年致癌性试验中，灌胃给予伏诺拉生 0.6、20、60、200mg/kg/日。在伏诺拉生 200mg/kg/日剂量下存活率下降，雄性存活动物数不足 20 只时（第 88 周）开始停药，不足 15 只时（第 90 周）对所有存活动物实施提前剖检。在≥6mg/kg/日剂量下雄性动物及≥60mg/kg/日剂量下雌性动物中，可见胃神经内分泌细胞肿瘤，200mg/kg/日剂量下可见胃腺瘤；≥20mg/kg/日剂量下雄性动物及≥60mg/kg/日剂量下雌性动物可见肝细胞腺瘤；≥60mg/kg/日剂量下雄性动物及 200mg/kg/日剂量下雌性动物可见肝细胞癌。

大鼠 2 年致癌性试验中，灌胃给予伏诺拉生 5、15、50、150 mg/kg/日，≥5mg/kg/日剂量下可见胃神经内分泌细胞肿瘤，≥50mg/kg/日剂量下可见肝细胞肿瘤，50 和 150mg/kg/日剂量下分别可见 1 只雄性动物肝胆管细胞腺瘤和 3 只雄性动物肝胆管细胞癌。

胃相关肿瘤的发生认为与抑制胃酸分泌引起的高胃泌素血症有关。肝细胞肿瘤可能是啮齿类动物特异的结果，与肝药酶的诱导有关。

【药代动力学】

1.单次给药条件下的药代动力学

下表列出了在空腹和餐后接受单次给予伏诺拉生 20mg 的健康成年男性受试者中伏诺拉生的药代动力学参数（均值±S.D.）。

剂量条件	中国人（平行试验设计）		日本人（交叉试验设计）	
	空腹（n=11）	餐后（n=10）	空腹（n=12）	餐后（n=12）
T_{max}(h)	2.0（0.75, 4.0）	2.5（0.75, 4.0）	1.5（1.0, 3.0）	3.0（1.0, 4.0）
C_{max}(ng/mL)	18.2±4.5	25.2±10.0	24.3±6.6	26.8±9.6
$t_{1/2z}$(h)	7.0±1.0	7.1±0.7	7.7±1.0	7.7±1.2
AUC_{∞}(h・ng/mL)	188.1±43.1	226.8±67.1	225.3±71.9	241.8±73.5

T_{max}表示为中位数（最小值和最大值）

2. 连续给药条件下的药代动力学

健康成年男性受试者每日一次接受伏诺拉生 10mg 或 20mg，连续给药 7 天，$AUC_{\tau,ss}$和 $C_{max,ss}$随着剂量的增加而成比例地增加，并在给药第 5 天达到稳态。日本人群在给药后第 3 天达到稳态。此外，伏诺拉生的药代动力学不具有时间依赖性。下表给出了给药第 7 天伏诺拉生的药代动力学参数（均值±S.D.）。

	中国人			日本人	
剂量	10mg QD（n=12）	20mg QD（n=11）	20mg BID（n=10）	10mg QD（n=9）	20mg QD（n=9）
$T_{max,ss}$ (h)	1.50（0.75, 3.02）	2.00（0.75, 3.00）	3.00（0.75, 4.00）	1.5（0.75, 3.0）	1.5（0.75, 3.0）
$C_{max,ss}$ (ng/mL)	11.0±3.49	23.4±6.14	37.9±10.9	12.0±1.8	23.3±6.6
$t_{1/2z}$ (h)	7.6±1.3	7.6±0.7	5.3±1.0	7.0±1.6	6.1±1.2
$AUC_{\tau,ss}$(h・ng/mL)	93.9±28.1	213±57.2	273±71.8	79.5±16.1	151.6±40.3

T_{max}表示为中位数（最小值和最大值）

3. 蛋白结合率

将 0.1-10μg/mL 范围内的[^{14}C]伏诺拉生加入人血浆中（体外），测得的蛋白结合率为 85.2% - 88.0%。

4. 代谢

（1）伏诺拉生主要通过肝脏药物代谢酶 CYP3A4 进行代谢，部分通过 CYP2B6、CYP2C19 和 CYP2D6 进行代谢。伏诺拉生还通过磺基转移酶 SULT2A1 进行代谢（体外）。

（2）伏诺拉生在体外表现出针对 CYP2B6、CYP2C19 和 CYP3A4/5 的时间依赖性抑制作用。此外，伏诺拉生针对 CYP1A2 显示出了轻微的浓度依赖性诱导效应，而对 CYP2B6 和 CYP3A4/5

则几乎无任何诱导作用（体外）。

5. 排泄

非日本人健康成年男性受试者接受放射性标记药物（伏诺拉生 15mg）口服给药后 168 小时，98.5%的放射性药物排泄至尿液和粪便中：67.4%药物排泄至尿液，31.1%药物排泄至粪便。

6. 肝功能障碍患者的药代动力学

比较肝功能正常受试者与轻度、中度和重度肝功能障碍患者的伏诺拉生药代动力学作用的国外临床试验显示，受试者接受伏诺拉生 20mg 后，轻度、中度和重度肝功能障碍患者的 AUC_{∞}和 C_{max}分别为肝功能正常受试者的 1.2～2.6 倍以及 1.2～1.8 倍。

7. 肾病患者的药代动力学

比较肾功能正常受试者与轻度、中度和重度肾功能障碍患者以及终末期肾病（ESRD）患者的伏诺拉生药代动力学作用的国外临床试验显示，受试者接受伏诺拉生 20mg 给药后，轻度、中度和重度肾功能障碍患者的 AUC_{∞}和 C_{max}分别为肾功能正常受试者的 1.3～2.4 倍以及 1.2～1.8 倍，显示伏诺拉生的暴露量随着肾功能的降低而升高。ESRD 患者的 AUC_{∞}和 C_{max}分别为肾功能正常患者的 1.3 倍和 1.2 倍。

8. 药物相互作用

（1）伏诺拉生和克拉霉素合并用药的药代动力学

在非日本人健康成年男性受试者中进行的药物相互作用研究中，受试者于第 1 天和第 8 天早餐后 30 分钟接受单剂量伏诺拉生 40mg 给药，并于第 3～9 天早餐前 30 分钟和晚餐前 30 分钟接受克拉霉素 500mg（效价）每日两次重复给药，研究结果显示，与克拉霉素合并用药的伏诺拉生的 AUC_{∞}和 C_{max}分别为单独给药时的 1.6 倍和 1.4 倍。

（2）伏诺拉生、阿莫西林水合物和克拉霉素合并用药的药代动力学

此项药物相互作用研究中，健康成年男性受试者接受伏诺拉生 20mg 与阿莫西林水合物 750mg（效价）和克拉霉素 400mg（效价）合并用药，每日两次，持续 7 天，研究显示原型阿莫西林的药代动力学并未受到影响，但伏诺拉生的 $AUC_{(0-12)}$和 C_{max}分别增高了 1.8 倍和 1.9 倍，原型克拉霉素的 $AUC_{(0-12)}$和 C_{max}分别增高了 1.5 倍和 1.6 倍。

（3）伏诺拉生、低剂量阿司匹林或伏诺拉生、NSAID 合并用药的药代动力学

此项药物相互作用研究中，健康成年男性受试者接受伏诺拉生 40mg 与阿司匹林 100mg 或 NSAID（洛索洛芬钠 60mg，双氯芬酸钠 25mg 或美洛昔康 10mg）合并给药，研究结果显示，低剂量阿司匹林或 NSAID 对伏诺拉生的药代动力学并无明确影响，伏诺拉生对低剂量阿

司匹林或NSAID的药代动力学亦无明确影响。

【贮藏】30℃以下密闭保存。

【包装】铝塑泡罩包装，7片/盒；10片/盒；14片/盒。

【有效期】36个月

【执行标准】进口药品注册标准：JX20190049

【批准文号】

进口药品注册证号：

10mg（按 $C_{17}H_{16}FN_3O_2S$ 计）：H20190064；H20201011

20mg（按 $C_{17}H_{16}FN_3O_2S$ 计）：H20190065；H20201005

批准文号：

10mg（按 $C_{17}H_{16}FN_3O_2S$ 计）：国药准字J20200015

20mg（按 $C_{17}H_{16}FN_3O_2S$ 计）：国药准字J20200011

【上市许可持有人】

企业名称：Takeda Pharmaceutical Company Limited

注册地址：1-1, Doshomachi 4-chome, Chuo-ku, Osaka 540-8645, Japan

【生产企业】

企业名称：Takeda Pharmaceutical Company Limited, Hikari Plant

注册地址：4720, Takeda, Mitsui, Hikari, Yamaguchi 743-8502, Japan

分装厂名称：天津武田药品有限公司

分装厂地址：天津市西青区兴华道11号

国内联系单位：

名称：武田药品（中国）有限公司

地址：江苏省泰州市药城大道836号

邮编：225300

电话：400-069-0980

传真：0523-86201595
