# Supplementary material for: Different dosages of vonoprazan for gastroesophageal reflux disease: study protocol for a pragmatic, crossover-cluster, randomized controlled trial with patient preference arms
Source: Trials. 2023 Dec 1;24:778. doi: 10.1186/s13063-023-07760-9 (PMC10691065; doi:10.1186/s13063-023-07760-9)
Supplement: Supplementary file 2 — Additional file 2. [file 13063_2023_7760_MOESM2_ESM.pdf]

2020年３月改訂（第１版）

貯法：室温保存
有効期間：３年

日本標準商品分類番号
872329

カリウムイオン競合型アシッドブロッカー
－プロトンポンプインヒビター－
ボノプラザンフマル酸塩錠

タケキャブ®錠10mg
タケキャブ®錠20mg
Takecab® Tablets 10mg & 20mg

	錠10mg	錠20mg
承認番号	22600AMX01389	22600AMX01390
販売開始	2015年２月	2015年２月

規制区分：処方箋医薬品[注)]
注）注意－医師等の処方箋により使用すること

2. 禁忌（次の患者には投与しないこと）
2.1 本剤の成分に対し過敏症の既往歴のある患者
2.2 アタザナビル硫酸塩、リルピビリン塩酸塩を投与中の患者［10.1参照］

3. 組成・性状
3.1 組成

販売名	タケキャブ錠10mg	タケキャブ錠20mg
有効成分	1錠中<br>ボノプラザンフマル酸塩13.36mg<br>（ボノプラザンとして10mg）	1錠中<br>ボノプラザンフマル酸塩26.72mg<br>（ボノプラザンとして20mg）
添加剤	D-マンニトール、結晶セルロース、クロスカルメロースナトリウム、ヒドロキシプロピルセルロース、フマル酸、ステアリン酸マグネシウム、ヒプロメロース、マクロゴール6000、酸化チタン、黄色三二酸化鉄	D-マンニトール、結晶セルロース、クロスカルメロースナトリウム、ヒドロキシプロピルセルロース、フマル酸、ステアリン酸マグネシウム、ヒプロメロース、マクロゴール6000、酸化チタン、三二酸化鉄

3.2 製剤の性状

販売名		タケキャブ錠10mg	タケキャブ錠20mg
色調・剤形		微黄色のフィルムコーティング錠	微赤色の両面割線入りのフィルムコーティング錠
製剤表示		タケキャブ10	タケキャブ20
形状	上面	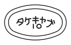	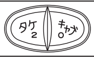
	下面	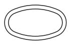	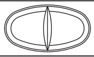
	側面	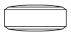	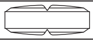
長径（mm）		8.2	11.2
短径（mm）		4.7	6.2
厚さ（mm）		約3.4	約3.9
質量（mg）		約115	約229

4. 効能又は効果
○胃潰瘍、十二指腸潰瘍、逆流性食道炎、低用量アスピリン投与時における胃潰瘍又は十二指腸潰瘍の再発抑制、非ステロイド性抗炎症薬投与時における胃潰瘍又は十二指腸潰瘍の再発抑制
○下記におけるヘリコバクター・ピロリの除菌の補助
胃潰瘍、十二指腸潰瘍、胃MALTリンパ腫、特発性血小板減少性紫斑病、早期胃癌に対する内視鏡的治療後胃、ヘリコバクター・ピロリ感染胃炎

5. 効能又は効果に関連する注意
〈低用量アスピリン投与時における胃潰瘍又は十二指腸潰瘍の再発抑制〉
5.1 血栓・塞栓の形成抑制のために低用量のアスピリンを継続投与している患者を投与対象とし、投与開始に際しては、胃潰瘍又は十二指腸潰瘍の既往を確認すること。
〈非ステロイド性抗炎症薬投与時における胃潰瘍又は十二指腸潰瘍の再発抑制〉
5.2 関節リウマチ、変形性関節症等における疼痛管理等のために非ステロイド性抗炎症薬を長期継続投与している患者を投与対象とし、投与開始に際しては、胃潰瘍又は十二指腸潰瘍の既往を確認すること。
〈ヘリコバクター・ピロリの除菌の補助〉
5.3 進行期胃MALTリンパ腫に対するヘリコバクター・ピロリ除菌治療の有効性は確立していない。
5.4 特発性血小板減少性紫斑病に対しては、ガイドライン等を参照し、ヘリコバクター・ピロリ除菌治療が適切と判断される症例にのみ除菌治療を行うこと。
5.5 早期胃癌に対する内視鏡的治療後胃以外には、ヘリコバクター・ピロリ除菌治療による胃癌の発症抑制に対する有効性は確立していない。
5.6 ヘリコバクター・ピロリ感染胃炎に用いる際には、ヘリコバクター・ピロリが陽性であること及び内視鏡検査によりヘリコバクター・ピロリ感染胃炎であることを確認すること。

6. 用法及び用量
〈胃潰瘍、十二指腸潰瘍〉
通常、成人にはボノプラザンとして1回20mgを1日1回経口投与する。なお、通常、胃潰瘍では8週間まで、十二指腸潰瘍では6週間までの投与とする。
〈逆流性食道炎〉
通常、成人にはボノプラザンとして1回20mgを1日1回経口投与する。なお、通常4週間までの投与とし、効果不十分の場合は8週間まで投与することができる。
さらに、再発・再燃を繰り返す逆流性食道炎の維持療法においては、1回10mgを1日1回経口投与するが、効果不十分の場合は、1回20mgを1日1回経口投与することができる。
〈低用量アスピリン投与時における胃潰瘍又は十二指腸潰瘍の再発抑制〉
通常、成人にはボノプラザンとして1回10mgを1日1回経口投与する。
〈非ステロイド性抗炎症薬投与時における胃潰瘍又は十二指腸潰瘍の再発抑制〉
通常、成人にはボノプラザンとして1回10mgを1日1回経口投与する。
〈ヘリコバクター・ピロリの除菌の補助〉
通常、成人にはボノプラザンとして1回20mg、アモキシシリン水和物として1回750mg（力価）及びクラリスロマイシンとして1回200mg（力価）の3剤を同時に1日2回、7日間経口投与する。なお、クラリスロマイシンは、必要に応じて適宜増量することができる。ただし、1回400mg（力価）1日2回を上限とする。
プロトンポンプインヒビター、アモキシシリン水和物及びクラリスロマイシンの3剤投与によるヘリコバクター・ピロリの除菌治療が不成功の場合は、これに代わる治療として、通常、成人にはボノプラザンとして1回20mg、アモキシシリン水和物として1回750mg（力価）及びメトロニダゾールとして1回250mgの3剤を同時に1日2回、7日間経口投与する。

8. 重要な基本的注意
〈効能共通〉
8.1 本剤の長期投与にあたっては、定期的に内視鏡検査を実施するなど観察を十分行うこと。
〈逆流性食道炎〉
8.2 維持療法においては、再発・再燃を繰り返す患者に対し投与することとし、本来維持療法の必要のない患者に投与することのないよう留意すること。寛解状態が長期にわたり継続する症例で、再発するおそれがないと判断される場合は1回20mgから1回10mgへの減量又は休薬を考慮すること。

9. 特定の背景を有する患者に関する注意
9.2 腎機能障害患者
本剤の排泄が遅延することにより血中濃度が上昇することがある。［16.6.1参照］
9.3 肝機能障害患者
本剤の代謝、排泄が遅延することにより血中濃度が上昇することがある。［16.6.2参照］
9.5 妊婦
妊婦又は妊娠している可能性のある女性には治療上の有益性が危険性を上回ると判断される場合にのみ投与すること。動物試験（ラット）において、最大臨床用量（40mg/日）におけるボノプラザンの曝露量（AUC）の約28倍を超える曝露量で、胎児体重及び胎盤重量の低値、外表異常（肛門狭窄及び尾の異常）、並びに内臓異常（膜性部心室中隔欠損及び鎖骨下動脈起始異常）が認められている。
9.6 授乳婦
治療上の有益性及び母乳栄養の有益性を考慮し、授乳の継続又は中止を検討すること。動物試験（ラット）で母乳中へ移行することが報告されている。
9.7 小児等
小児等を対象とした臨床試験は実施していない。
9.8 高齢者
一般に高齢者では肝機能、腎機能等の生理機能が低下している。

10. 相互作用

本剤は主として肝薬物代謝酵素CYP3A4で代謝され、一部CYP2B6、CYP2C19及びCYP2D6で代謝される。
また、本剤の胃酸分泌抑制作用により、併用薬剤の吸収を促進又は抑制する可能性がある。

10.1 併用禁忌（併用しないこと）

薬剤名等	臨床症状・措置方法	機序・危険因子
アタザナビル硫酸塩<br>（レイアタッツ）<br>[2.2参照]	アタザナビル硫酸塩の作用を減弱するおそれがある。	本剤の胃酸分泌抑制作用によりアタザナビル硫酸塩の溶解性が低下し、アタザナビルの血中濃度が低下する可能性がある。
リルピビリン塩酸塩<br>（エジュラント）<br>[2.2参照]	リルピビリン塩酸塩の作用を減弱するおそれがある。	本剤の胃酸分泌抑制作用によりリルピビリン塩酸塩の吸収が低下し、リルピビリンの血中濃度が低下する可能性がある。

10.2 併用注意（併用に注意すること）

薬剤名等	臨床症状・措置方法	機序・危険因子
CYP3A4阻害剤<br>クラリスロマイシン 等<br>[16.7.1、16.7.2参照]	本剤の血中濃度が上昇する可能性がある。	クラリスロマイシンとの併用により本剤の血中濃度が上昇したとの報告がある。
ジゴキシン<br>メチルジゴキシン	左記薬剤の作用を増強する可能性がある。	本剤の胃酸分泌抑制作用によりジゴキシンの加水分解が抑制され、ジゴキシンの血中濃度が上昇する可能性がある。
イトラコナゾール<br>チロシンキナーゼ阻害剤<br>ゲフィチニブ<br>ニロチニブ<br>エルロチニブ<br>ネルフィナビルメシル酸塩	左記薬剤の作用を減弱する可能性がある。	本剤の胃酸分泌抑制作用により左記薬剤の血中濃度が低下する可能性がある。

11. 副作用

次の副作用があらわれることがあるので、観察を十分に行い、異常が認められた場合には投与を中止するなど適切な処置を行うこと。

11.1 重大な副作用

〈効能共通〉

11.1.1 汎血球減少、無顆粒球症、白血球減少、血小板減少（いずれも頻度不明）

11.1.2 中毒性表皮壊死融解症（Toxic Epidermal Necrolysis：TEN）、皮膚粘膜眼症候群（Stevens-Johnson症候群）、多形紅斑（いずれも頻度不明）

〈ヘリコバクター・ピロリの除菌の補助〉

11.1.3 偽膜性大腸炎等の血便を伴う重篤な大腸炎（頻度不明）
ヘリコバクター・ピロリの除菌に用いるアモキシシリン水和物、クラリスロマイシンでは、偽膜性大腸炎等の血便を伴う重篤な大腸炎があらわれることがあるので、腹痛、頻回の下痢があらわれた場合には直ちに投与を中止するなど適切な処置を行うこと。

11.2 その他の副作用

〈胃潰瘍、十二指腸潰瘍、逆流性食道炎、低用量アスピリン投与時における胃潰瘍又は十二指腸潰瘍の再発抑制、非ステロイド性抗炎症薬投与時における胃潰瘍又は十二指腸潰瘍の再発抑制〉

	0.1～5%未満
消化器	便秘、下痢、腹部膨満感、悪心
過敏症	発疹
肝　臓	AST、ALT、AL-P、LDH、γ-GTPの上昇
その他	浮腫、好酸球増多

〈ヘリコバクター・ピロリの除菌の補助〉

	5%以上	0.1～5%未満
消化器	下痢（10.6%）	味覚異常、口内炎、腹部不快感、腹部膨満感
過敏症		発疹
肝　臓		AST、ALTの上昇

表中の頻度表示は胃潰瘍又は十二指腸潰瘍におけるボノプラザンフマル酸塩、アモキシシリン水和物及びクラリスロマイシンの3剤投与の試験成績に基づく。

14. 適用上の注意

14.1 薬剤交付時の注意

PTP包装の薬剤はPTPシートから取り出して服用するよう指導すること。PTPシートの誤飲により、硬い鋭角部が食道粘膜へ刺入し、更には穿孔をおこして縦隔洞炎等の重篤な合併症を併発することがある。

15. その他の注意

15.1 臨床使用に基づく情報

15.1.1 本剤の長期投与中に良性の胃ポリープを認めたとの報告がある。

15.1.2 本剤の投与が胃癌による症状を隠蔽することがあるので、悪性でないことを確認のうえ投与すること。

15.1.3 海外における複数の観察研究で、プロトンポンプインヒビターによる治療において骨粗鬆症に伴う股関節骨折、手関節骨折、脊椎骨折のリスク増加が報告されている。特に、高用量及び長期間（1年以上）の治療を受けた患者で、骨折のリスクが増加した。

15.1.4 海外における主に入院患者を対象とした複数の観察研究で、プロトンポンプインヒビターを投与した患者においてクロストリジウム・ディフィシルによる胃腸感染のリスク増加が報告されている。

15.2 非臨床試験に基づく情報

マウス及びラット2年間経口投与がん原性試験において、臨床用量（20mg/日）におけるボノプラザンの曝露量（AUC）と等倍程度の曝露量で胃の神経内分泌腫瘍が、約300倍で胃の腺腫（マウス）が、また、約13倍以上（マウス）及び約58倍以上（ラット）で肝臓腫瘍が認められている。

16. 薬物動態

16.1 血中濃度

16.1.1 反復投与

健康成人男性を対象に10mg又は20mgを1日1回7日間反復経口投与した時、投与7日目のボノプラザンのAUC $_{(0\text{-tau})}$ 及びCmaxは投与量の増加に伴い増加し、これらの増加の程度は投与量比をわずかに上回る。また、ボノプラザンの血中濃度のトラフ値は、投与3日目から7日目まで一定であり、投与3日目までに定常状態に達していると考えられる。さらに、ボノプラザンのAUC $_{(0\text{-tau})}$ 及びT1/2に関する蓄積性評価の結果から、反復投与時のボノプラザンの薬物動態に時間依存性はないと考えられる。投与7日目のボノプラザンの薬物動態学的パラメータは下表のとおりである[1]。

10mg又は20mg反復投与時の薬物動態学的パラメータ（健康成人男性）

投与量	10mg	20mg
T_{max}（h）	1.5（0.75, 3.0）	1.5（0.75, 3.0）
C_{max}（ng/mL）	12.0±1.8	23.3±6.6
$T_{1/2}$（h）	7.0±1.6	6.1±1.2
AUC $_{(0\text{-tau})}$（ng・h/mL）	79.5±16.1	151.6±40.3

9例の平均値±標準偏差（ただし、Tmaxは中央値（最小値, 最大値））

16.2 吸収

16.2.1 食事の影響

健康成人男性を対象に20mgを絶食下及び食後に単回経口投与した時のボノプラザンの薬物動態学的パラメータ及び血中濃度推移は以下のとおりであり、薬物動態に及ぼす食事の影響はほとんどみられなかった[2]。

20mg絶食下及び食後単回投与時の薬物動態学的パラメータ（健康成人男性）

投与条件	絶食下	食後
T_{max}（h）	1.5（1.0, 3.0）	3.0（1.0, 4.0）
C_{max}（ng/mL）	24.3±6.6	26.8±9.6
$T_{1/2}$（h）	7.7±1.0	7.7±1.2
$AUC_{0\text{-}48}$（ng・h/mL）	222.1±69.7	238.3±71.1

12例の平均値±標準偏差（ただし、Tmaxは中央値（最小値, 最大値））

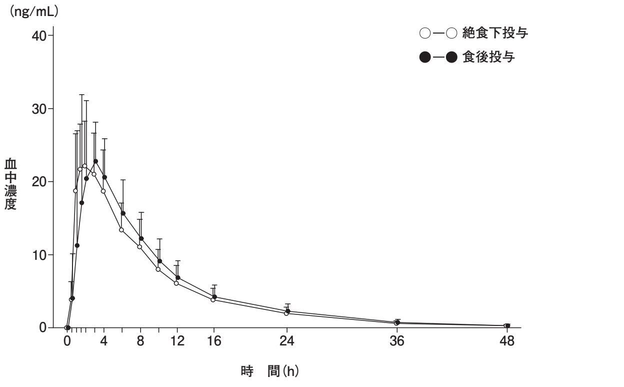

20mg絶食下及び食後単回投与時のボノプラザンの血中濃度推移

16.3 分布

16.3.1 蛋白結合率

[^{14}C] ボノプラザンを0.1～10μg/mLの範囲でヒト血漿に添加した時の蛋白結合率は、85.2～88.0%である（*in vitro*）[3]。

16.4 代謝

16.4.1 ボノプラザンは主としてCYP3A4で代謝され、一部CYP2B6、CYP2C19及びCYP2D6で代謝される。また、硫酸転移酵素SULT2A1でも代謝される（*in vitro*）[4,5]。

16.4.2 ボノプラザンは、CYP2B6、CYP2C19及びCYP3A4/5に対して時間依存的な阻害作用を示す（*in vitro*）。また、ボノプラザンは、濃度依存的なCYP1A2誘導作用をわずかに示すが、CYP2B6及びCYP3A4/5誘導作用はほとんど示さない（*in vitro*）[6,7]。

16.5 排泄

外国人健康成人男性を対象に放射性標識体（ボノプラザンとして15mg）を経口投与したとき、投与168時間後までに、投与された放射能の98.5%が尿及び糞便中に排泄される。このうち、67.4%が尿中へ、31.1%が糞便中へ排泄される[8]。

16.6 特定の背景を有する患者

16.6.1 腎機能障害患者

腎機能正常者（eGFR：90mL/min/1.73m^{2}以上）、軽度（eGFR：60～89mL/min/1.73m^2）、中等度（eGFR：30～59mL/min/1.73m^2）及び高度腎機能障害（eGFR：15～29mL/min/1.73m^2）のある患者、並びに末期腎

不全（ESRD）（eGFR：15mL/min/1.73m^{2}未満）患者を対象にボノプラザンとして20mgを投与した時の薬物動態に及ぼす腎機能障害の影響を検討した外国で実施した臨床試験において、ボノプラザンのAUC$_{0\text{-}inf}$及びC$_{max}$は、軽度、中等度及び高度腎機能障害のある患者では腎機能正常者と比較してそれぞれ1.3～2.4倍及び1.2～1.8倍高く、腎機能の低下に伴い増加し、また、ESRD患者におけるAUC$_{0\text{-}inf}$及びC$_{max}$は、腎機能正常者と比較してそれぞれ1.3倍及び1.2倍高い[9]。［9.2参照］

16.6.2 肝機能障害患者

肝機能正常者、並びに軽度（Child-Pugh分類スコアA）、中等度（Child-Pugh分類スコアB）及び高度肝機能障害（Child-Pugh分類スコアC）のある患者を対象にボノプラザンとして20mgを投与した時の薬物動態に及ぼす肝機能障害の影響を検討した外国で実施した臨床試験において、ボノプラザンのAUC$_{0\text{-}inf}$及びC$_{max}$は、軽度、中等度及び高度肝機能障害のある患者では肝機能正常者と比較してそれぞれ1.2～2.6倍及び1.2～1.8倍高い[10]。［9.3参照］

16.7 薬物相互作用

16.7.1 ボノプラザン、クラリスロマイシン併用時の薬物動態

外国健康成人男性を対象に1日目及び8日目にボノプラザンとして40mgを朝食30分後に単回投与し、3～9日目にクラリスロマイシンとして500mg（力価）を1日2回、朝夕食30分前に反復投与した試験の結果、ボノプラザンのAUC$_{0\text{-}inf}$及びC$_{max}$は、単独投与時と比較してクラリスロマイシンとの併用投与時に1.6倍及び1.4倍増加する[11]。［10.2参照］

16.7.2 ボノプラザン、アモキシシリン水和物及びクラリスロマイシン併用時の薬物動態

健康成人男性を対象にボノプラザンとして20mg、アモキシシリン水和物として750mg（力価）及びクラリスロマイシンとして400mg（力価）の3剤を同時に1日2回、7日間併用投与した試験の結果、アモキシシリン未変化体の薬物動態に及ぼす影響は見られないものの、3剤併用投与によりボノプラザンのAUC$_{0\text{-}12}$及びC$_{max}$はそれぞれ1.8倍及び1.9倍増加し、クラリスロマイシン未変化体のAUC$_{0\text{-}12}$及びC$_{max}$はそれぞれ1.5倍及び1.6倍増加する[12]。［10.2参照］

16.7.3 ボノプラザン、低用量アスピリン又はボノプラザン、非ステロイド性抗炎症薬併用時の薬物動態

健康成人男性を対象にボノプラザン40mg、アスピリン100mg又は非ステロイド性抗炎症薬（ロキソプロフェンナトリウム60mg、ジクロフェナクナトリウム25mg又はメロキシカム10mg）を併用投与した試験の結果、ボノプラザンの薬物動態に及ぼす低用量アスピリン又は非ステロイド性抗炎症薬の影響、及び低用量アスピリン又は非ステロイド性抗炎症薬の薬物動態に及ぼすボノプラザンの影響について、いずれも明らかな影響は見られなかった[13]。

17. 臨床成績

17.1 有効性及び安全性に関する試験

〈胃潰瘍、十二指腸潰瘍〉

17.1.1 国内第Ⅲ相試験（二重盲検比較試験）

胃潰瘍、十二指腸潰瘍患者を対象に、ボノプラザン20mg又はランソプラゾール30mgを1日1回最大8週間（胃潰瘍）及び最大6週間（十二指腸潰瘍）経口投与した時の疾患別治癒率は下表のとおりであり、胃潰瘍患者を対象とした試験では、ランソプラゾール群に対するボノプラザン群の非劣性が認められたが、十二指腸潰瘍患者を対象とした試験では、ランソプラゾール群に対するボノプラザン群の非劣性は認められなかった[14,15]。

胃潰瘍及び十二指腸潰瘍の治癒率

疾患名	ボノプラザン20mg	ランソプラゾール30mg
胃潰瘍	93.5%（216/231例）	93.8%（211/225例）
	-0.3%［-4.750%, 4.208%］注1）<br>p＝0.0011注2）	
十二指腸潰瘍	95.5%（170/178例）	98.3%（177/180例）
	-2.8%［-6.400%, 0.745%］注1）<br>p＝0.0654注3）	

（　）は治癒例数/評価例数
注1）投与群間差、［　］は両側95%信頼区間
注2）許容限界値を8%とした、Farrington and Manningによる非劣性検定
注3）許容限界値を6%とした、Farrington and Manningによる非劣性検定

胃潰瘍患者を対象とした試験の副作用発現頻度はボノプラザン群では6.6%（16/244例）であった。主な副作用は、便秘（5例）、下痢（1例）及び食道カンジダ症（1例）であった。

また、十二指腸潰瘍患者を対象とした試験の副作用発現頻度はボノプラザン群では9.3%（17/183例）であった。主な副作用は、下痢（3例）及び便秘（1例）であった。

〈逆流性食道炎〉

17.1.2 国内第Ⅲ相試験（二重盲検比較試験）

逆流性食道炎患者を対象に、ボノプラザン20mg又はランソプラゾール30mgを1日1回最大8週間経口投与した時の投与4週後及び8週後までの治癒率は下表のとおりであり、投与8週後までの治癒率についてランソプラゾール群に対するボノプラザン群の非劣性が認められた。また、ボノプラザン群の投与4週後までの治癒率とランソプラゾール群の投与8週後までの治癒率の差の点推定値（両側95%信頼区間）は1.1%（-2.702～4.918%）であった[16]。

逆流性食道炎の治癒率

投与期間	ボノプラザン20mg	ランソプラゾール30mg
4週後	96.6%（198/205例）	92.5%（184/199例）
	4.1%［-0.308%, 8.554%］注1）	
8週後	99.0%（203/205例）	95.5%（190/199例）
	3.5%［0.362%, 6.732%］注1）<br>p＜0.0001注2）	

（　）は治癒例数/評価例数
注1）投与群間差、［　］は両側95%信頼区間
注2）許容限界値を10%とした、Farrington and Manningによる非劣性検定

副作用発現頻度はボノプラザン群では6.8%（14/207例）であった。主な副作用は、腹部膨満（3例）、便秘（2例）、食道カンジダ症（1例）及び好酸球数増加（1例）であった。

〈逆流性食道炎の維持療法〉

17.1.3 国内第Ⅲ相試験（単盲検長期投与試験）

上記17.1.2試験で治癒が確認され、上記試験を完了した患者を対象に、さらにボノプラザン10mg又は20mgを1日1回52週間投与した時の再発率は、10mg群で9.4%（14/149例）、20mg群で9.0%（13/145例）であった[17]。

副作用発現頻度はボノプラザン10mg群では9.7%（15/154例）、ボノプラザン20mg群では16.6%（25/151例）であった。主な副作用は、胃ポリープ（ボノプラザン10mg群1例、ボノプラザン20mg群3例）及び肝機能検査異常（ボノプラザン20mg群3例）であった。

17.1.4 国内第Ⅲ相試験（二重盲検比較試験）

ボノプラザン20mgを1日1回最大8週間経口投与することにより治癒と判定された逆流性食道炎の患者を対象に、さらに維持療法としてボノプラザン10mg、20mg又はランソプラゾール15mgを1日1回24週間経口投与した時の再発率は下表のとおりであり、ランソプラゾール群に対するボノプラザン10mg群及び20mg群の非劣性が認められた[18]。

逆流性食道炎の再発率

ボノプラザン10mg	ボノプラザン20mg	ランソプラゾール15mg
5.1%（10/197例）	2.0%（4/201例）	16.8%（33/196例）
＜ボノプラザン10mg群vsランソプラゾール15mg群＞<br>-11.8%［-17.830%, -5.691%］注1）、p＜0.0001注2）<br>＜ボノプラザン20mg群vsランソプラゾール15mg群＞<br>-14.8%［-20.430%, -9.264%］注1）、p＜0.0001注2）		

（　）は再発例数/評価例数
注1）投与群間差、［　］は両側95%信頼区間
注2）許容限界値を10%とした、Farrington and Manningによる非劣性検定

副作用発現頻度は、ボノプラザン10mg群では10.4%（21/202例）、ボノプラザン20mg群では10.3%（21/204例）であった。主な副作用は、下痢（ボノプラザン10mg群0例、ボノプラザン20mg群1例、以下同順）、胃ポリープ（3例、1例）、便秘（0例、2例）、血中クレアチンホスホキナーゼ増加（1例、2例）、アラニンアミノトランスフェラーゼ増加（1例、1例）及びγ-グルタミルトランスフェラーゼ増加（0例、1例）であった。

〈低用量アスピリン投与時における胃潰瘍又は十二指腸潰瘍の再発抑制〉

17.1.5 国内第Ⅲ相試験（二重盲検比較試験）

低用量アスピリン（1日81～324mg）の長期投与を必要とし、かつ胃潰瘍又は十二指腸潰瘍の既往歴を有する患者を対象に、ボノプラザン10mg又はランソプラゾール15mgを1日1回24週間経口投与した時の投与24週後の潰瘍再発率は下表のとおりであり、ランソプラゾール群に対するボノプラザン群の非劣性が認められた[19]。

低用量アスピリン投与時における胃潰瘍又は十二指腸潰瘍の再発率（二重盲検）

ボノプラザン10mg	ランソプラゾール15mg
0.5%（1/197例）	2.8%（6/213例）
-2.3%［-4.743%, 0.124%］注1）<br>p＜0.0001注2）	

（　）は再発例数/評価例数
注1）投与群間差、［　］は両側95%信頼区間
注2）許容限界値を8.7%とした、Farrington and Manningによる非劣性検定

副作用発現頻度は、ボノプラザン群では10.4%（21/202例）であった。主な副作用は、便秘（2例）、下痢（1例）及び血中鉄減少（4例）であった。

17.1.6 国内第Ⅲ相試験（単盲検長期投与試験）

上記17.1.5試験を終了した患者を対象に、さらに最短28週、最長80週間ボノプラザン10mg又はランソプラゾール15mgを1日1回継続投与した時の潰瘍再発率は下表のとおりであった[20]。

低用量アスピリン投与時における胃潰瘍又は十二指腸潰瘍の再発率（単盲検）

ボノプラザン10mg	ランソプラゾール15mg
0.5%（1/197例）	3.3%（7/213例）
-2.8%［-5.371%, -0.187%］注）	

（　）は再発例数/評価例数
注）投与群間差、［　］は両側95%信頼区間

副作用発現頻度は、ボノプラザン群では16.3%（33/202例）であった。主な副作用は、便秘（2例）、下痢（2例）、血中鉄減少（4例）及び高血圧（1例）であった。

〈非ステロイド性抗炎症薬投与時における胃潰瘍又は十二指腸潰瘍の再発抑制〉

17.1.7 国内第Ⅲ相試験（二重盲検比較試験）

関節リウマチ、変形性関節症等の疼痛管理のために、非ステロイド性抗炎症薬の長期投与を必要とし、かつ胃潰瘍又は十二指腸潰瘍の既往歴を有する患者を対象に、ボノプラザン10mg又はランソプラゾール15mgを1日1回24週間経口投与した時の投与24週後の潰瘍再発率は下表のとおりであり、ランソプラゾール群に対するボノプラザン群の非劣性が認められた[21]。

非ステロイド性抗炎症薬投与時における胃潰瘍又は十二指腸潰瘍の再発率（二重盲検）

ボノプラザン10mg	ランソプラゾール15mg
3.3%（7/209例）	5.5%（11/199例）
-2.2%［-6.182%, 1.826%］注1）<br>p＜0.0001注2）	

（　）は再発例数/評価例数
注1）投与群間差、［　］は両側95%信頼区間
注2）許容限界値を8.3%とした、Farrington and Manningによる非劣性検定

副作用発現頻度は、ボノプラザン群では15.6%（34/218例）であった。主な副作用は、下痢（2例）及び便秘（5例）であった。

17.1.8 国内第Ⅲ相試験（単盲検長期投与試験）

上記17.1.7試験を終了した患者を対象に、さらに最短28週、最長80週間ボノプラザン10mg又はランソプラゾール15mgを1日1回継続投与した時の潰瘍再発率は下表のとおりであった[22]。

非ステロイド性抗炎症薬投与時における胃潰瘍又は十二指腸潰瘍の再発率（単盲検）

ボノプラザン10mg	ランソプラゾール15mg
3.8%（8/209例）	7.5%（15/199例）
-3.7%［-8.207%, 0.787%］[注)]	

（ ）は再発例数/評価例数
注）投与群間差、［ ］は両側95%信頼区間

副作用発現頻度は、ボノプラザン群では17.4%（38/218例）であった。主な副作用は、下痢（3例）、便秘（5例）及び血中アルカリホスファターゼ増加（2例）であった。

〈胃潰瘍又は十二指腸潰瘍におけるヘリコバクター・ピロリ感染〉

17.1.9 国内第Ⅲ相試験（二重盲検比較試験）

（1）一次除菌

ヘリコバクター・ピロリ陽性の胃潰瘍又は十二指腸潰瘍瘢痕患者を対象に、ボノプラザン20mg又はランソプラゾール30mg、アモキシシリン水和物及びクラリスロマイシンの3剤を1日2回7日間経口投与した時の除菌率は下表のとおりであり、ランソプラゾールを用いた3剤併用療法群に対するボノプラザンを用いた3剤併用療法群の非劣性が認められた[23]。

ヘリコバクター・ピロリ一次除菌[注1)]率

各薬剤の1回投与量	除菌率	群間差
ボノプラザン20mg<br>アモキシシリン水和物750mg（力価）<br>クラリスロマイシン200mg（力価）<br>又は400mg（力価）	92.6%<br>（300/324例）	16.7%<br>［11.172%, 22.138%］[注2)]<br>p＜0.0001[注3)]
ランソプラゾール30mg<br>アモキシシリン水和物750mg（力価）<br>クラリスロマイシン200mg（力価）<br>又は400mg（力価）	75.9%<br>（243/320例）	

（ ）は除菌成功例数/評価例数
注1）^{13}C-尿素呼気試験の結果が陰性
注2）投与群間差、［ ］は両側95%信頼区間
注3）許容限界値を10%としたFarrington and Manningによる非劣性検定

副作用発現頻度は、ボノプラザン群では20.4%（67/329例）であった。主な副作用は、下痢（35例）及び味覚異常（13例）であった。

（2）二次除菌

ボノプラザン又はランソプラゾールと、アモキシシリン水和物及びクラリスロマイシンの3剤投与によるヘリコバクター・ピロリの除菌が不成功であった50例を対象に、ボノプラザン20mg、アモキシシリン水和物及びメトロニダゾールの3剤を1日2回7日間経口投与した時の除菌率は下表のとおりであった[23]。

ヘリコバクター・ピロリ二次除菌[注)]率

各薬剤の1回投与量	除菌率
ボノプラザン20mg<br>アモキシシリン水和物750mg（力価）<br>メトロニダゾール250mg	98.0%（49/50例）

（ ）は除菌成功例数/評価例数
注）^{13}C-尿素呼気試験の結果が陰性

副作用発現頻度は、16.0%（8/50例）であった。主な副作用は、下痢、鼓腸、アラニンアミノトランスフェラーゼ増加及びアスパラギン酸アミノトランスフェラーゼ増加（各2例）であった。

17.3 その他

17.3.1 血清ガストリンに及ぼす影響

ボノプラザンを1日1回10mg又は20mgを経口投与した場合、血清ガストリン値はランソプラゾール群に比べてボノプラザン群で持続的に高値を示した。低用量アスピリン投与時における胃潰瘍又は十二指腸潰瘍の再発抑制の長期投与試験における血清ガストリン値の推移図は以下のとおりであった。なお、投与終了後に血清ガストリン値の回復を確認した胃潰瘍、十二指腸潰瘍患者を対象とした臨床試験では、速やかな回復が認められた（投与終了後2〜8週間）[14, 15, 20, 22]。

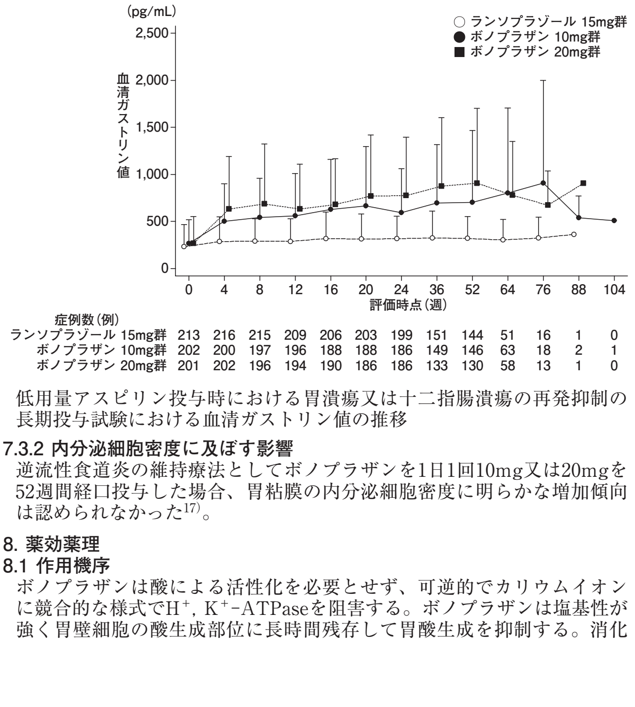

症例数（例）													
ランソプラゾール 15mg群	213	216	215	209	206	203	199	151	144	51	16	1	0
ボノプラザン 10mg群	202	200	197	196	188	188	186	149	146	63	18	2	1
ボノプラザン 20mg群	201	202	196	194	190	186	186	133	130	58	13	1	0

低用量アスピリン投与時における胃潰瘍又は十二指腸潰瘍の再発抑制の長期投与試験における血清ガストリン値の推移

17.3.2 内分泌細胞密度に及ぼす影響

逆流性食道炎の維持療法としてボノプラザンを1日1回10mg又は20mgを52週間経口投与した場合、胃粘膜の内分泌細胞密度に明らかな増加傾向は認められなかった[17]。

18. 薬効薬理

18.1 作用機序

ボノプラザンは酸による活性化を必要とせず、可逆的でカリウムイオンに競合的な様式でH^+, K^+-ATPaseを阻害する。ボノプラザンは塩基性が強く胃壁細胞の酸生成部位に長時間残存して胃酸生成を抑制する。消化管上部の粘膜損傷形成に対して、ボノプラザンは強い抑制作用を示す。ボノプラザンは抗ヘリコバクター・ピロリ活性及びヘリコバクター・ピロリウレアーゼ阻害活性は示さない[24]。

18.2 胃酸分泌抑制作用

健康成人男性において、ボノプラザン10mg又は20mgの7日間反復投与により24時間中に胃内pHが4以上を示す時間の割合は、それぞれ63±9%又は83±17%であった[2]。

18.3 ヘリコバクター・ピロリ除菌の補助作用

ヘリコバクター・ピロリ除菌治療におけるボノプラザンの役割は胃内pHを上昇させることにより、併用されるアモキシシリン水和物、クラリスロマイシン、メトロニダゾールの抗菌活性を高めることにあると考えられる。

19. 有効成分に関する理化学的知見

一般名：ボノプラザンフマル酸塩（Vonoprazan Fumarate）〔JAN〕
化学名：1-[5-(2-Fluorophenyl)-1-(pyridin-3-ylsulfonyl)-1*H*-pyrrol-3-yl]-*N*-methylmethanamine monofumarate
分子式：$C_{17}H_{16}FN_3O_2S \cdot C_4H_4O_4$
分子量：461.46
化学構造式：

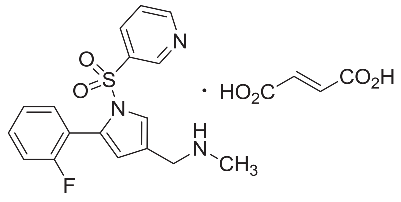

性状：ボノプラザンフマル酸塩は白色〜ほとんど白色の結晶又は結晶性の粉末である。ジメチルスルホキシドにやや溶けやすく、*N,N*-ジメチルアセトアミドにやや溶けにくく、メタノール及び水に溶けにくく、2-プロパノール及びアセトニトリルにほとんど溶けない。
融点：194.8℃

21. 承認条件

医薬品リスク管理計画を策定の上、適切に実施すること。

22. 包装

〈タケキャブ錠10mg〉
PTP　100錠（10錠×10）、500錠（10錠×50）、瓶（500錠バラ）

〈タケキャブ錠20mg〉
PTP　100錠（10錠×10）、500錠（10錠×50）、瓶（500錠バラ）

23. 主要文献

1）ボノプラザンの薬物動態試験成績①（2014年12月26日承認：CTD 2.7.6.4）
2）ボノプラザンの薬物動態試験成績②（2014年12月26日承認：CTD 2.7.6.1）
3）ボノプラザンの薬物動態試験成績③（2014年12月26日承認：CTD 2.6.4.4）
4）ボノプラザンの薬物動態試験成績④（2014年12月26日承認：CTD 2.6.4.5）
5）ボノプラザンの薬物動態試験成績⑤（2014年12月26日承認：CTD 2.6.4.5）
6）ボノプラザンの薬物動態試験成績⑥（2014年12月26日承認：CTD 2.6.4.5）
7）ボノプラザンの薬物動態試験成績⑦（2014年12月26日承認：CTD 2.6.4.5）
8）ボノプラザンの薬物動態試験成績⑧（2014年12月26日承認：CTD 2.7.6.6）
9）ボノプラザンの薬物動態試験成績⑨（2014年12月26日承認：CTD 2.7.6.10）
10）ボノプラザンの薬物動態試験成績⑩（2014年12月26日承認：CTD 2.7.6.9）
11）ボノプラザンの薬物動態試験成績⑪（2014年12月26日承認：CTD 2.7.6.13）
12）ボノプラザンの薬物動態試験成績⑫（2014年12月26日承認：CTD 2.7.6.12）
13）ボノプラザンの薬物動態試験成績⑬（2014年12月26日承認：CTD 2.7.6.11）
14）ボノプラザンの臨床試験成績①（2014年12月26日承認：CTD 2.7.6.17）
15）ボノプラザンの臨床試験成績②（2014年12月26日承認：CTD 2.7.6.18）
16）ボノプラザンの臨床試験成績③（2014年12月26日承認：CTD 2.7.6.15）
17）ボノプラザンの臨床試験成績④（2014年12月26日承認：CTD 2.7.6.22）
18）ボノプラザンの臨床試験成績⑤（2014年12月26日承認：CTD 2.7.6.16）
19）ボノプラザンの臨床試験成績⑥（2014年12月26日承認：CTD 2.7.6.20）
20）ボノプラザンの臨床試験成績⑦（2014年12月26日承認：CTD 2.7.6.23）
21）ボノプラザンの臨床試験成績⑧（2014年12月26日承認：CTD 2.7.6.21）
22）ボノプラザンの臨床試験成績⑨（2014年12月26日承認：CTD 2.7.6.24）
23）ボノプラザンの臨床試験成績⑩（2014年12月26日承認：CTD 2.7.6.19）
24）ボノプラザンの薬理試験成績（2014年12月26日承認：CTD 2.6.2.6）

24. 文献請求先及び問い合わせ先

武田薬品工業株式会社 くすり相談室
〒103-8668 東京都中央区日本橋本町二丁目1番1号
フリーダイヤル 0120-566-587
受付時間 9：00 〜 17：30（土日祝日・弊社休業日を除く）

大塚製薬株式会社 医薬情報センター
〒108-8242 東京都港区港南2-16-4 品川グランドセントラルタワー
電話 0120-189-840

26. 製造販売業者等

26.1 製造販売元

武田薬品工業株式会社
〒540-8645 大阪市中央区道修町四丁目1番1号

26.2 提携

大塚製薬株式会社
〒101-8535 東京都千代田区神田司町 2-9
