# Supplementary material for: Different dosages of vonoprazan for gastroesophageal reflux disease: study protocol for a pragmatic, crossover-cluster, randomized controlled trial with patient preference arms
Source: Trials. 2023 Dec 1;24:778. doi: 10.1186/s13063-023-07760-9 (PMC10691065; doi:10.1186/s13063-023-07760-9)
Supplement: Supplementary file 4 — Additional file 4. [file 13063_2023_7760_MOESM4_ESM.docx]

Dear :

You will be invited to participate in the clinical study of Wuhan Union Hospital on " Different dosages of vonoprazan for gastroesophageal reflux disease: a pragmatic, crossover-cluster, randomized controlled trail" The following items describe the background, objectives, methods, benefits and the possible risks, as well as your rights and interests of this study. Please be sure to read the following carefully before you participate in this study. The information in this informed consent form can help you decide whether to participate in this study. If you have any questions, please ask the investigator responsible for this study to ensure you fully understand the relevant content. If you agree to participate in this clinical study voluntarily, please sign the statement of your informed consent form.

The medical ethics committee of Union Hospital, Tongji Medical College of Huazhong University of Science and Technology, has approved this study.

**Background**

Gastroesophageal Reflux Disease (GERD) is that the reflux of gastric contents causes uncomfortable symptoms and complications. Proton Pump Inhibitors (PPIs), including omeprazole, rabeprazole, and esomeprazole, are first-line drugs for treating GERD. However, studies have shown that PPI response rates in patients with GERD at 4 and 8 weeks are only 44% and 64%, respectively. Vonoprazan is a new potassium competitive acid blocker (P-CAB) that can achieve the highest acid inhibition effect immediately after taking the drug. Compared with PPIs, it achieved faster acid inhibition and longer duration of action. Moreover, the healing rate of esophageal mucosa for patients with esophagitis is significantly higher than PPIs.

Vonoprazan was approved for treating GERD at 2019 in China. Its efficacy and safety have been verified, and it has become a routine drug in the management of GERD patients and has entered the health insurance catalog in China. A small sample size of clinical study has confirmed that the relief rate of symptoms of patients with GERD is 70–80%, and the mucosal healing rate is 75–85%. Thus, 10–15% of patients with GERD still do not respond to vonoprazan. Therefore, we need to increase the sample size to measure the effectiveness of vonoprazan at different doses and dosing times in the real-world clinical trail in China.

**Objectives**

This study aims to explore the effectiveness of vonoprazan in controlling symptoms and mucosal healing in patients with GERD at different doses and dosing times.

**Who can participate in this study?**

1. Entry criteria

1) ≥18 years old

2) Patients having completed upper gastrointestinal endoscopy in the past one year

3) Patients with typical symptoms (heartburn or regurgitation) for at least one month and GERD-Q≥8; or patients with untypical symptoms (retrosternal burning pain or discomfort) for at least one month and erosive esophagitis grade B/C/D;

4) Adherence to the allocated regimens and the follow-up procedure

2. Exclusions

1) Patients taking gastric acid inhibited drugs (PPIs, H2RA and other PCABs) and prokinetic drugs, in the last 1 week;

2) Patients with acute peptic ulcer, previous gastric or esophageal surgery history;

3) Patients with serious diseases such as hepatic failure, renal insufficiency and cancers;

4) Pregnant and lactating women;

5) Patients with communication and coordination disorders

**Introduction of the study**

This real-world study is a multi-centered, prospective, and pragmatic randomized controlled trial enrolling patients diagnosed with GERD. The medication used in this study, vonoprazan, is routinely used in the clinical practice and has been entered into Chinese health insurance catalog. This study will only collect the diagnosis and treatment information of patients with GERD during the treatment, and no other particular intervention will be given.

Grouping method:

This study was randomly divided into three groups according to cluster randomization: 1) vonoprazan pre/post-breakfast dosing group, 2) vonoprazan pre/post-dinner dosing group, and 3)vonoprazan double dose group. During the study, participants could choose any treatment according to the preference.

1. Vonoprazan pre/post-breakfast dosing group: Vonoprazan 20mg once daily, orally either before or after breakfast;
2. Vonoprazan pre/post-dinner dosing group: Vonoprazan 20mg once daily, orally either before or after dinner;
3. Vonoprazan double dose group: Vonoprazan 20mg twice daily, orally before/after breakfast and before/after dinner, respectively.

All of the treatment duration of the three groups will be up to 4 weeks.

Information collection:

The participants are required to record the symptoms daily and participate in online follow-up to assess the symptom relief in the fourth week of medication. Besides, the patients with esophagitis are required to complete endoscopy again to assess the mucosal healing. Patients should also participate the online follow-up to assess long-term symptom control in the fifth month after treatment.

**Obligations of participants**

You (Participant) should complete the drug treatment for four weeks and keep a symptom diary during the medication period. You should complete the first follow-up visit after four weeks of drug treatment, and the patient with esophagitis should also complete the upper gastrointestinal endoscopy after treatment. You should complete the second follow-up at the fifth month after your treatment.

**Possible risks of participating in this study**

1. Risks associated with research interventions

The treatment procedures in this study meets the clinical practice standards for diagnosis and treatment of GERD without additional risk.

The related drug vonoprazan has been approved in China, and its efficacy and safety have been verified. It is routinely used in the management of GERD. Possible risks include drug allergy and adverse effects, as indicated in the drug instructions.

The involved invasive operation will be an upper gastrointestinal endoscopy, which is the recommended reexamination item for patients with esophagitis. The involved risks were endoscopic-related.

2. Other risks:

Personal privacy disclosure risk. In this study, the collected personal data are stored by abbreviation or code, to ensure that your privacy will not be disclosed.

**Possible benefits of participating in this study**

Vonoprazan, in this study, was approved and marketed in China in 2019 as a drug for treating GERD, and its efficacy and safety have been verified. Compared with the PPIs for the routine treatment of GERD, the current treatment has a better acid inhibition effect, thus bringing benefits to your treatment.

If you agree to participate in this study, you may or may not have direct medical benefits. If you participate in this study, you can take medications more appropriately. We also hope that your information obtained from this study will benefit more patients with GERD in the future.

**What to do if a study-related injury occurs?**

This is a real-world study in which the drug involved, vonoprazan, is an effective therapeutic drug for GERD, but treatment-related injuries may still occur. If not enrolled in this study, you may still be vulnerable to injury from related treatment. The investigator will handle related injuries according to relevant medical procedures but is not responsible for the expenses and compensation for related injuries.

**Can you obtain compensation for participating in this study?**

As this study is designed to explore the efficacy of vonoprazan in treating patients with GERD in the real-world situation, only your clinical diagnosis and treatment data will be collected during the study. Your will be responsible to pay the involved drugs and daily medical expenses without additional compensation.

**How to deal with the new clinical research information?**

The investigator will identify and promptly inform you of any new information that may affect your continued participation in this study.

**Which conditions may terminate the clinical study?**

The investigator may terminate the study if you:

1) Poor compliance; 2) When adverse effects or other clinical conditions occurs, it is no longer in the best interest of the patient to continue to participate in the study; 3) Study intervention needs to be suspended due to the disease progresses; 4) Participants meet the exclusion criteria (new or confirmed); 5) Participants will not be eligible for study intervention for a certain period.

**How long will this study last?**

The duration of this study was six month.

**How many people will be enrolled in this study?**

2880.

**Privacy and confidentiality**

All your information (including visit records) in this study will be kept confidential, and available only to the investigators. When necessary, the government management department and the hospital ethics committee can access to your information according to the provisions. The results of this study will be published in future without any patient privacy implications.

**Right to voluntarily choose to participate in and withdraw from the study.**

Whether you participate in this study is entirely voluntary, and if you do not, you can refuse to participate without any negative impact on your health care. Even after participating, you can withdraw from the study without any reason. We encourage you to discuss this with your doctor when you decide not to participate in the study.

**How to Get Help in a Study**

When there are questions about the study information, study progress and interests of participants, and any discomfort and injuries related to the study, you can contact the researcher at phone number +86 15802734363, or contact the medical ethics committee: the Medical Ethics Committee of Union Hospital Affiliated to Tongji Medical College of Huazhong University of Science and Technology at 027-85726375.

**Participant Statement**

I have read this Informed Consent Form carefully. I have had the opportunity to ask questions, and all have been answered. I understand that participation in this study is voluntary. I can choose not to participate or withdraw from this study after informing the investigator without discrimination or retaliation. Any of my medical treatment and rights will not be affected thereby.

The investigator could terminate my participation in this clinical study if additional diagnosis/treatment is required, if I am not following the study plan, or for other reasonable reasons.

I volunteered to participate in this clinical trial and will receive a signed copy of my Informed Consent Form.

Participant Name (BLOCK LETTERS): Tel:

Participant Signature: Date:

If the participant cannot sign informed consent due to incapacity or the participant is a minor, his/her guardian shall sign the consent.

Name of guardian (BLOCK LETTERS): Tel:

Signature of guardian: Date：

Relationship to participant:

Reasons why a participant cannot sign an informed consent form:

Signed by a fair witness when the participant or their guardian cannot read.

Name of witness (BLOCK LETTERS): Tel:

Signature of witness: Date：

**Investigator Statement**

I accurately informed the participants of the informed consent form and answered their questions and the participants volunteered for this clinical trial.

Name of Investigator (BLOCK LETTERS): Tel:

Signature of Investigator: Date:
