# Supplementary material for: Different dosages of vonoprazan for gastroesophageal reflux disease: study protocol for a pragmatic, crossover-cluster, randomized controlled trial with patient preference arms
Source: Trials. 2023 Dec 1;24:778. doi: 10.1186/s13063-023-07760-9 (PMC10691065; doi:10.1186/s13063-023-07760-9)
Supplement: Supplementary file 3 — Additional file 3. [file 13063_2023_7760_MOESM3_ESM.pdf]

NDA 215152
NDA 215153

NDA APPROVAL

Phathom Pharmaceuticals, Inc.
Attention: Nancianne Knipfer, PhD, RAC
Senior Director, Regulatory Affairs
2150 East Lake Cook Road, Suite 800
Buffalo Grove, IL 60089

Dear Dr. Knipfer:

Please refer to your new drug applications (NDAs) dated and received September 3, 2021, and your amendments, submitted pursuant to section 505(b)(2) of the Federal Food, Drug, and Cosmetic Act (FDCA) for the following:

- NDA 215152: Voquezna Triple Pak (vonoprazan tablets; amoxicillin capsules; clarithromycin tablets) 20 mg, 500 mg, 500 mg, co-packaged

- NDA 215153: Voquezna Dual Pak (vonoprazan tablets; amoxicillin capsules) 20 mg, 500 mg, co-packaged

These NDAs provide for the use of Voquezna Triple Pak and Voquezna Dual Pak for the treatment of *Helicobacter pylori* (*H. pylori*) infection in adults.

We have completed our review of these applications, as amended. They are approved, effective on the date of this letter, for use as recommended in the enclosed agreed-upon labeling text.

WAIVER OF ½ PAGE LENGTH REQUIREMENT FOR HIGHLIGHTS

We are waiving the requirements of 21 CFR 201.57(d)(8) regarding the length of the Highlights of Prescribing Information. This waiver applies to all future supplements containing revised labeling unless we notify you otherwise.

CONTENT OF LABELING

As soon as possible, but no later than 14 days from the date of this letter, submit the content of labeling [21 CFR 314.50(l)] in structured product labeling (SPL) format using the FDA automated drug registration and listing system (eLIST), as described at FDA.gov.[1] Content of labeling must be identical to the enclosed labeling (text for the Prescribing Information) as well as annual reportable changes not included in the

enclosed labeling. Information on submitting SPL files using eLIST may be found in the guidance for industry *SPL Standard for Content of Labeling Technical Qs and As.*[2]

The SPL will be accessible via publicly available labeling repositories.

CARTON AND CONTAINER LABELING

Submit final printed carton and container labeling that are identical to the enclosed carton and container labeling as soon as they are available, but no more than 30 days after they are printed. Please submit the labeling electronically according to the guidance for industry *Providing Regulatory Submissions in Electronic Format — Certain Human Pharmaceutical Product Applications and Related Submissions Using the eCTD Specifications*. For administrative purposes, designate these submissions as "**Final Printed Carton and Container Labeling for approved NDA 215152"** and "**Final Printed Carton and Container Labeling for approved NDA 215153"**. Approval of these submissions by FDA are not required before the labeling is used.

DATING PERIOD

Based on the stability data submitted to date, the expiry dating period for Voquezna Triple Pak and Voquezna Dual Pak shall be 24 months from the date of manufacture when stored at 20°C to 25°C (68°F to 77°F). Brief excursion permitted to 15° C to 30° C (59° F to 86°F). [See USP Controlled Room Temperature.]

ADVISORY COMMITTEE

Your applications for Voquezna Triple Pak and Voquezna Dual Pak were not referred to an FDA advisory committee because the applications did not raise significant safety or efficacy issues that were unexpected for a drug of this class.

REQUIRED PEDIATRIC ASSESSMENTS

Under the Pediatric Research Equity Act (PREA) (21 U.S.C. 355c), all applications for new active ingredients (which includes new salts and new fixed combinations), new indications, new dosage forms, new dosing regimens, or new routes of administration are required to contain an assessment of the safety and effectiveness of the product for the claimed indication in pediatric patients unless this requirement is waived, deferred, or inapplicable.

We are waiving the pediatric study(ies) requirement for these applications because necessary studies are impossible or highly impracticable as the prevalence of *H. pylori* infection that requires treatment is low in the pediatric population.

POSTMARKETING REQUIREMENTS UNDER 505(o)

Section 505(o)(3) of the FDCA authorizes FDA to require holders of approved drug and biological product applications to conduct postmarketing studies and clinical trials for certain purposes, if FDA makes certain findings required by the statute.

We have determined that an analysis of spontaneous postmarketing adverse events reported under subsection 505(k)(1) of the FDCA will not be sufficient to identify an unexpected serious risk of congenital malformations, spontaneous abortions, stillbirths, elective terminations, small for gestational age, preterm birth, and any other adverse pregnancy outcomes and also to evaluate the long-term safety of vonoprazan-containing products in lactating women, including assessing risks of adverse effects on the developing neonate. We have also determined that the extent of various cytochrome P450 (CYP) enzymes' contributions to the metabolism of vonoprazan has not been adequately characterized to assess the risk of increased vonoprazan exposure and toxicity in patients with reduced CYP activity (e.g., poor metabolizers). In addition, the drug-drug interaction (DDI) potential of vonoprazan metabolite M-I-G has not been adequately characterized to assess overall DDI related risks with Voquezna Triple Pak and Voquezna Dual Pak

Furthermore, the active postmarket risk identification and analysis system as available under section 505(k)(3) of the FDCA will not be sufficient to assess these serious risks.

Therefore, based on appropriate scientific data, FDA has determined that you are required to conduct the following studies:

4270-1: Conduct a prospective, registry based, observational exposure cohort study that compares the maternal, fetal, and infant outcomes of women exposed to vonoprazan-containing products during pregnancy to an unexposed control population. The registry should be designed to detect and record major and minor congenital malformations, spontaneous abortions, stillbirths, elective terminations, small for gestational age, preterm birth, and any other adverse pregnancy outcomes. These outcomes will be assessed throughout pregnancy. Infant outcomes, including effects on postnatal growth and development, will be assessed through at least the first year of life.

The timetable you submitted on April 26, 2022, states that you will conduct this study according to the following schedule:

Draft protocol submission:	11/ 2022
Final protocol submission:	07/ 2023
Interim report:	07/ 2024
	07/ 2025
	07/ 2026
	07/ 2027

	07/ 2028
	07/ 2029
	07/ 2030
	07/ 2031
	07/ 2032
	07/ 2033
	07/ 2034
	07/ 2035
Study completion date:	07/ 2035
Final report submission date:	04/ 2036

4270-2: An additional pregnancy study that uses a different design from the Pregnancy Registry (for example, a retrospective cohort study using claims or electronic medical record data or a case control study) to assess major congenital malformations, spontaneous abortions, stillbirths, and small for gestational age and preterm birth in women exposed to vonoprazan-containing products during pregnancy compared to an unexposed control population.

The timetable you submitted on April 26, 2022, states that you will conduct this study according to the following schedule:

Draft protocol submission:	11/ 2022
Final protocol submission:	07/ 2023
Interim report:	07/ 2024
	07/ 2025
	07/ 2026
	07/ 2027
	07/ 2028
	07/ 2029
	07/ 2030
Study completion date:	07/ 2030
Final report submission date:	04/ 2031

4270-3: Conduct a lactation study (milk only) in lactating women who have received vonoprazan-containing products to assess concentrations of vonoprazan in breast milk using a validated assay. A mother-infant pair study may be required in the future depending on the results of this milk-only study.

The timetable you submitted on April 26, 2022, states that you will conduct this study according to the following schedule:

Draft protocol submission:	11/ 2022
Final protocol submission:	06/ 2023
Study completion date:	08/ 2024

Final report submission date: 05/ 2025

4270-4: Conduct a post-marketing in vitro reaction phenotyping study with selective chemical inhibitors to determine the role of cytochrome P450 (CYP) enzymes (e.g., CYP3A4/5, CYP2B6, CYP2C19, CYP2C9, and CYP2D6) in vonoprazan metabolism at clinically relevant concentrations under the linear condition. This study should be designed and conducted in accordance with the FDA Guidance for Industry entitled "In Vitro Drug Interaction Studies - Cytochrome P450 Enzyme- and Transporter-Mediated Drug Interactions". Depending on the results of this in vitro study, additional studies may be needed.

The timetable you submitted on April 26, 2022, states that you will conduct this study according to the following schedule:

Draft protocol submission:	07/ 2022
Final protocol submission:	09/ 2022
Study completion date:	12/ 2022
Final report submission date:	03/ 2023

4270-5: Conduct post-marketing in vitro DDI studies to evaluate the inhibition potential of vonoprazan metabolite M-I-G on CYP enzymes (except CYP3A) and transporters.

The timetable you submitted on April 29, 2022, states that you will conduct this study according to the following schedule:

Draft protocol submission:	01/ 2023
Final protocol submission:	03/ 2023
Study completion date:	07/ 2023
Final report submission date:	12/ 2023

FDA considers the term *final* to mean that the applicant has submitted a protocol, the FDA review team has sent comments to the applicant, and the protocol has been revised as needed to meet the goal of the study or clinical trial.

Submit clinical protocol(s) to your IND 143190 (vonoprazan, amoxicillin, clarithromycin) and IND 144399 (vonoprazan, amoxicillin) with a cross-reference letter to these NDAs. Submit nonclinical and chemistry, manufacturing, and controls protocols and all final report(s) to your NDAs. Prominently identify the submission with the following wording in bold capital letters at the top of the first page of the submission, as appropriate: **Required Postmarketing Protocol Under 505(o), Required Postmarketing Final Report Under 505(o), Required Postmarketing Correspondence Under 505(o).**

Submission of the protocol(s) for required postmarketing observational studies to your INDs is for the purposes of administrative tracking only. These studies do not constitute clinical investigations pursuant to 21 CFR 312.3(b) and therefore are not subject to the IND requirements under 21 CFR part 312.

Section 505(o)(3)(E)(ii) of the FDCA requires you to report periodically on the status of any study or clinical trial required under this section. This section also requires you to periodically report to FDA on the status of any study or clinical trial otherwise undertaken to investigate a safety issue. Section 506B of the FDCA, as well as 21 CFR 314.81(b)(2)(vii) requires you to report annually on the status of any postmarketing commitments or required studies or clinical trials.

FDA will consider the submission of your annual report under section 506B and 21 CFR 314.81(b)(2)(vii) to satisfy the periodic reporting requirement under section 505(o)(3)(E)(ii) provided that you include the elements listed in 505(o) and 21 CFR 314.81(b)(2)(vii). We remind you that to comply with 505(o), your annual report must also include a report on the status of any study or clinical trial otherwise undertaken to investigate a safety issue. Failure to submit an annual report for studies or clinical trials required under 505(o) on the date required will be considered a violation of FDCA section 505(o)(3)(E)(ii) and could result in enforcement action.

PROMOTIONAL MATERIALS

You may request advisory comments on proposed introductory advertising and promotional labeling. For information about submitting promotional materials, see the final guidance for industry *Providing Regulatory Submissions in Electronic and Non-Electronic Format—Promotional Labeling and Advertising Materials for Human Prescription Drugs.*[3]

As required under 21 CFR 314.81(b)(3)(i), you must submit final promotional materials, and the Prescribing Information, at the time of initial dissemination or publication, accompanied by a Form FDA 2253. Form FDA 2253 is available at FDA.gov.[4] Information and Instructions for completing the form can be found at FDA.gov.[5]

REPORTING REQUIREMENTS

We remind you that you must comply with reporting requirements for your approved NDAs (21 CFR 314.80 and 314.81).

POST APPROVAL FEEDBACK MEETING

New molecular entities qualify for a post approval feedback meeting. Such meetings are used to discuss the quality of the application and to evaluate the communication process during drug development and marketing application review. The purpose is to learn from successful aspects of the review process and to identify areas that could

Reference ID: 4978225

benefit from improvement. If you would like to have such a meeting with us, call the Regulatory Project Manager for this application.

If you have any questions, call Eva Zuffova, PhD, Regulatory Project Manager, at (301) 796-0697.

Sincerely,

{See appended electronic signature page}

John Farley, MD, MPH
Director
Office of Infectious Diseases
Office of New Drugs
Center for Drug Evaluation and Research

ENCLOSURE(S):

- Content of Labeling
    - Prescribing Information
    - Carton and Container Labeling

HIGHLIGHTS OF PRESCRIBING INFORMATION
These highlights do not include all the information needed to use VOQUEZNA™ TRIPLE PAK™ and VOQUEZNA™ DUAL PAK™ safely and effectively. See full prescribing information for VOQUEZNA TRIPLE PAK and VOQUEZNA DUAL PAK.

VOQUEZNA TRIPLE PAK (vonoprazan tablets; amoxicillin capsules; clarithromycin tablets), co-packaged for oral use
VOQUEZNA DUAL PAK (vonoprazan tablets; amoxicillin capsules) co-packaged for oral use
Initial U.S. Approval: 2022

INDICATIONS AND USAGE

VOQUEZNA TRIPLE PAK, is a co-packaged product containing vonoprazan, a potassium-competitive acid blocker (PCAB), amoxicillin, a penicillin class antibacterial, and clarithromycin, a macrolide antimicrobial, indicated for the treatment of *Helicobacter pylori (H. pylori)* infection in adults. (1.1)

VOQUEZNA DUAL PAK, is a co-packaged product containing vonoprazan, a PCAB, and amoxicillin, a penicillin class antibacterial, indicated for the treatment of *H. pylori* infection in adults. (1.1)

To reduce the development of drug-resistant bacteria and maintain the effectiveness of VOQUEZNA TRIPLE PAK, VOQUEZNA DUAL PAK and other antibacterial drugs, VOQUEZNA TRIPLE PAK and VOQUEZNA DUAL PAK should be used only to treat or prevent infections that are proven or strongly suspected to be caused by bacteria. (1.2)

DOSAGE AND ADMINISTRATION

VOQUEZNA TRIPLE PAK: The recommended dosage regimen is vonoprazan 20 mg plus amoxicillin 1,000 mg plus clarithromycin 500 mg, each given twice daily (morning and evening, 12 hours apart), with or without food, for 14 days. (2.1)

VOQUEZNA DUAL PAK: The recommended dosage regimen is vonoprazan 20 mg twice daily (morning and evening) plus amoxicillin 1,000 mg, three times a day (morning, mid-day, and evening), with or without food, for 14 days. (2.2)

DOSAGE FORMS AND STRENGTHS

VOQUEZNA TRIPLE PAK: Carton of 14 daily administration packs for morning and evening dosing, each containing the following three drug products (3.1):

- Tablets: Vonoprazan 20 mg
- Tablets: Clarithromycin 500 mg
- Capsules: Amoxicillin 500 mg

VOQUEZNA DUAL PAK: Carton of 14 daily administration packs for morning, mid-day and evening dosing, each containing the following two drug products (3.2):

- Tablets: Vonoprazan 20 mg
- Capsules: Amoxicillin 500 mg

CONTRAINDICATIONS

VOQUEZNA TRIPLE PAK and VOQUEZNA DUAL PAK:
- Known hypersensitivity to vonoprazan, amoxicillin or any other beta-lactams, clarithromycin or any other macrolide antimicrobial or any component of VOQUEZNA TRIPLE PAK. (4.1)
- Known hypersensitivity to vonoprazan, amoxicillin or any other beta-lactams or any component of VOQUEZNA DUAL PAK. (4.1)
- Rilpivirine-containing products. (4.1)

VOQUEZNA TRIPLE PAK Due to the Clarithromycin Component:
- Pimozide. (4.2)
- Lomitapide, lovastatin, and simvastatin. (4.2)
- Ergot alkaloids (ergotamine or dihydroergotamine). (4.2)
- Colchicine in renal or hepatic impairment. (4.2)
- History of cholestatic jaundice/hepatic dysfunction with use of clarithromycin. (4.2)

WARNINGS AND PRECAUTIONS

VOQUEZNA TRIPLE PAK and VOQUEZNA DUAL PAK:
- Hypersensitivity Reactions: Serious and occasionally fatal reactions (e.g., anaphylaxis) have been reported with components of VOQUEZNA TRIPLE PAK and VOQUEZNA DUAL PAK. If hypersensitivity reactions occur, discontinue VOQUEZNA TRIPLE PAK or VOQUEZNA DUAL PAK and institute immediate therapy (e.g., anaphylaxis management). (5.1)
- Severe Cutaneous Adverse Reactions (SCAR): Discontinue VOQUEZNA TRIPLE PAK or VOQUEZNA DUAL PAK at the first signs or symptoms of SCAR or other signs of hypersensitivity and consider further evaluation (5.1).
- *Clostridioides difficile*-associated diarrhea (CDAD): Evaluate if diarrhea occurs with VOQUEZNA TRIPLE PAK and VOQUEZNA DUAL PAK. (5.1)

VOQUEZNA TRIPLE PAK Due to the Clarithromycin Component:
- QT Prolongation: Avoid VOQUEZNA TRIPLE PAK in patients with known QT prolongation or receiving drugs known to prolong the QT interval, ventricular arrhythmia (*torsades de pointes*), hypokalemia/hypomagnesemia, significant bradycardia, or taking Class IA or III antiarrhythmics. (5.2)
- Hepatotoxicity: Discontinue if signs and symptoms of hepatitis occur with VOQUEZNA TRIPLE PAK. (5.2)
- Serious adverse reactions due to concomitant use with other drugs: Serious adverse reactions can occur with VOQUEZNA TRIPLE PAK due to drug interactions of clarithromycin with colchicine, some lipid lowering agents, some calcium channel blockers, and other drugs. (5.2)
- Embryo-Fetal Toxicity: Based on the findings from animal studies and human observational studies in pregnant women treated with clarithromycin, VOQUEZNA TRIPLE PAK is not recommended for use in pregnant women except in clinical circumstances where no alternative therapy is appropriate. (5.2)
- Myasthenia Gravis: Exacerbation of myasthenia gravis can occur with VOQUEZNA TRIPLE PAK since it has been reported in patients receiving clarithromycin tablets. (5.2)

ADVERSE REACTIONS

VOQUEZNA TRIPLE PAK: Most common adverse reactions (≥ 2%) were dysgeusia, diarrhea, vulvovaginal candidiasis, headache, abdominal pain, and hypertension. (6.1)

VOQUEZNA DUAL PAK: Most common adverse reactions (≥ 2%) were diarrhea, abdominal pain, vulvovaginal candidiasis and nasopharyngitis. (6.1)

To report SUSPECTED ADVERSE REACTIONS, contact Phathom Pharmaceuticals, Inc. at toll-free phone 1-800-775-PHAT (7428) or FDA at 1-800-FDA-1088 or www.fda.gov/medwatch.

DRUG INTERACTIONS

Components of VOQUEZNA TRIPLE PAK and VOQUEZNA DUAL PAK have the potential for clinically important drug interactions. See Full Prescribing Information for important drug interactions with VOQUEZNA TRIPLE PAK and VOQUEZNA DUAL PAK. (4, 5.2, 7)

USE IN SPECIFIC POPULATIONS

- Lactation: Breastfeeding not recommended during treatment, but a lactating woman can pump and discard breast milk during treatment and for 2 days after VOQUEZNA TRIPLE PAK or VOQUEZNA DUAL PAK administration. (8.2)
- Geriatrics: VOQUEZNA TRIPLE PAK increased risk of *torsades de pointes* due to the clarithromycin component. (8.5)
- Renal Impairment: Avoid use in severe renal impairment. (8.6)
- Hepatic Impairment: Avoid use in moderate and severe hepatic impairment. (8.7)

See 17 for PATIENT COUNSELING INFORMATION

Revised: 5/2022

FULL PRESCRIBING INFORMATION: CONTENTS*

1 INDICATIONS AND USAGE
- 1.1 *Helicobacter pylori* Infection
- 1.2 Usage

2 DOSAGE AND ADMINISTRATION
- 2.1 Recommended Dosage for VOQUEZNA TRIPLE PAK
- 2.2 Recommended Dosage for VOQUEZNA DUAL PAK
- 2.3 Missed Doses

3 DOSAGE FORMS AND STRENGTHS
- 3.1 VOQUEZNA TRIPLE PAK
- 3.2 VOQUEZNA DUAL PAK

4 CONTRAINDICATIONS
- 4.1 Contraindications to VOQUEZNA TRIPLE PAK and VOQUEZNA DUAL PAK
- 4.2 Additional Contraindications to VOQUEZNA TRIPLE PAK Due to the Clarithromycin Component

5 WARNINGS AND PRECAUTIONS
- 5.1 Warnings and Precautions for VOQUEZNA TRIPLE PAK and VOQUEZNA DUAL PAK
- 5.2 Additional Warnings and Precautions for VOQUEZNA TRIPLE PAK Due to the Clarithromycin Component

6 ADVERSE REACTIONS
- 6.1 Clinical Trials Experience
- 6.2 Postmarketing Experience with Components of VOQUEZNA TRIPLE PAK and VOQUEZNA DUAL PAK

7 DRUG INTERACTIONS

8 USE IN SPECIFIC POPULATIONS
- 8.1 Pregnancy
- 8.2 Lactation
- 8.3 Females and Males of Reproductive Potential
- 8.4 Pediatric Use
- 8.5 Geriatric Use
- 8.6 Renal Impairment
- 8.7 Hepatic Impairment

10 OVERDOSAGE

11 DESCRIPTION

12 CLINICAL PHARMACOLOGY
- 12.1 Mechanism of Action
- 12.2 Pharmacodynamics
- 12.3 Pharmacokinetics
- 12.4 Microbiology

13 NONCLINICAL TOXICOLOGY
- 13.1 Carcinogenesis, Mutagenesis, Impairment of Fertility
- 13.2 Animal Toxicology and/or Pharmacology

14 CLINICAL STUDIES

16 HOW SUPPLIED/STORAGE AND HANDLING

17 PATIENT COUNSELING INFORMATION

* Sections or subsections omitted from the full prescribing information are not listed.

FULL PRESCRIBING INFORMATION

1. INDICATIONS AND USAGE

1.1. *Helicobacter pylori* Infection

VOQUEZNA TRIPLE PAK or VOQUEZNA DUAL PAK are indicated for the treatment of *Helicobacter pylori* (*H. pylori*) infection in adults *[see Clinical Studies (14)].*

1.2. Usage

To reduce the development of drug-resistant bacteria and maintain the effectiveness of VOQUEZNA TRIPLE PAK, VOQUEZNA DUAL PAK and other antibacterial drugs, VOQUEZNA TRIPLE PAK and VOQUEZNA DUAL PAK should be used only to treat or prevent infections that are proven or strongly suspected to be caused by susceptible bacteria. When culture and susceptibility information are available, they should be considered in selecting or modifying antibacterial therapy. In the absence of such data, local epidemiology and susceptibility patterns may contribute to the empiric selection of therapy.

2. DOSAGE AND ADMINISTRATION

2.1. Recommended Dosage for VOQUEZNA TRIPLE PAK

The recommended adult oral dosage of VOQUEZNA TRIPLE PAK is vonoprazan 20 mg plus amoxicillin 1,000 mg plus clarithromycin 500 mg, each given twice daily (in the morning and evening, 12 hours apart), with or without food, for 14 days.

2.2. Recommended Dosage for VOQUEZNA DUAL PAK

The recommended adult oral dose of VOQUEZNA DUAL PAK is vonoprazan 20 mg given twice daily (in the morning and evening) plus amoxicillin 1,000 mg three times daily (in the morning, mid-day, and evening), with or without food, for 14 days.

2.3. Missed Doses

If a dose is missed, administer VOQUEZNA TRIPLE PAK or VOQUEZNA DUAL PAK as soon as possible, within 4 hours after the missed dose. If more than 4 hours have passed, skip the missed dose and administer the next dose on the regularly scheduled time. Patients should continue the normal dosing schedule until the medication is completed.

3. DOSAGE FORMS AND STRENGTHS

3.1. VOQUEZNA TRIPLE PAK

VOQUEZNA TRIPLE PAK is a co-package consisting of 14 administration packs for morning and evening dosing. Each administration pack contains the following three drug products:

- Vonoprazan Tablets, 20 mg: pale red, oval, film-coated tablets debossed V20 on one side and plain on the other side.
- Amoxicillin Capsules, 500 mg: yellow, opaque, hard gelatin capsules imprinted with AMOX 500 on one side and GG 849 on the other side.
- Clarithromycin Tablets, 500 mg: white, oval, film-coated debossed GG C9 on one side and plain on the other side.

3.2. VOQUEZNA DUAL PAK

VOQUEZNA DUAL PAK is a co-package consisting of 14 administration packs for morning, mid-day and evening dosing. Each administration pack contains the following two drug products:

- Vonoprazan Tablets, 20 mg: pale red, oval, film-coated tablets debossed V20 on one side and plain on the other side.
- Amoxicillin Capsules, 500 mg: yellow, opaque, hard gelatin capsules imprinted with AMOX 500 on one side and GG 849 on the other side.

4. CONTRAINDICATIONS

4.1. Contraindications to VOQUEZNA TRIPLE PAK and VOQUEZNA DUAL PAK

Hypersensitivity Reactions

VOQUEZNA TRIPLE PAK and VOQUEZNA DUAL PAK are contraindicated in patients with a known hypersensitivity to any component of VOQUEZNA TRIPLE PAK: vonoprazan, amoxicillin (or other β-lactam antibacterials, e.g., penicillins and cephalosporins) or clarithromycin (or other macrolide antibacterial drugs, e.g., erythromycin) or VOQUEZNA DUAL PAK: vonoprazan or amoxicillin (or other β-lactam antibacterials, e.g., penicillins and cephalosporins) *[see Warnings and Precautions (5.1)].*

Rilpivirine-containing Products

VOQUEZNA TRIPLE PAK and VOQUEZNA DUAL PAK are contraindicated with rilpivirine-containing products *[see Drug Interactions (7)].*

4.2. Additional Contraindications to VOQUEZNA TRIPLE PAK Due to the Clarithromycin Component

Serious Adverse Reactions/Risks Due to Drug Interactions

Because of the clarithromycin component, VOQUEZNA TRIPLE PAK is contraindicated with concomitant use of:

- Pimozide: There have been postmarketing reports of drug interactions when clarithromycin is co-administered with pimozide, resulting in cardiac arrhythmias (QT prolongation, ventricular tachycardia, ventricular fibrillation, and *torsades de pointes*) most likely due to inhibition of metabolism of these drugs by clarithromycin. Fatalities have been reported *[see Warnings and Precautions (5.2) and Drug Interactions (7)]*
- Lipid-lowering Agents: Lomitapide, simvastatin, and lovastatin *[see Warnings and Precautions (5.2) and Drug Interactions (7)].*
- Ergot Alkaloids: Ergotamine or dihydroergotamine *[see Drug Interactions (7)]*
- Colchicine in patients with renal or hepatic impairment *[see Warnings and Precautions (5.2) and Drug Interactions (7)]*

Cholestatic Jaundice/Hepatic Dysfunction

VOQUEZNA TRIPLE PAK is contraindicated in patients with a history of cholestatic jaundice or hepatic dysfunction associated with prior use of clarithromycin.

5. WARNINGS AND PRECAUTIONS

5.1. Warnings and Precautions for VOQUEZNA TRIPLE PAK and VOQUEZNA DUAL PAK

Hypersensitivity Reactions

Serious and occasionally fatal hypersensitivity reactions (e.g., anaphylaxis, anaphylactic shock, rash, erythema multiforme, and Henoch-Schonlein purpura) have been reported with components of VOQUEZNA TRIPLE PAK and VOQUEZNA DUAL PAK *[see Contraindications (4.1)].*

Before initiating therapy with VOQUEZNA TRIPLE PAK or VOQUEZNA DUAL PAK careful inquiry should be made regarding previous hypersensitivity reactions to penicillins, cephalosporins, macrolide antibacterial drugs or other allergens. Discontinue VOQUEZNA TRIPLE PAK or VOQUEZNA DUAL PAK immediately and institute appropriate treatment if hypersensitivity occurs.

Severe Cutaneous Adverse Reactions

Severe cutaneous adverse reactions (SCAR), including Stevens-Johnson syndrome (SJS) and toxic epidermal necrolysis (TEN) have been reported with the components of VOQUEZNA TRIPLE PAK: vonoprazan, amoxicillin and clarithromycin and VOQUEZNA DUAL PAK: vonoprazan and amoxicillin *[see Adverse Reactions (6.2)]*. In addition, drug reaction with eosinophilia and systemic symptoms (DRESS), and acute generalized exanthematous pustulosis (AGEP) have been reported with amoxicillin and clarithromycin.

Discontinue VOQUEZNA TRIPLE PAK or VOQUEZNA DUAL PAK at the first signs or symptoms of SCAR or other signs of hypersensitivity and consider further evaluation.

Clostridioides difficile-Associated Diarrhea

Clostridioides difficile-associated diarrhea (CDAD) has been reported with use of acid suppressing therapies and nearly all antibacterial agents, including amoxicillin (component of VOQUEZNA DUAL PAK and TRIPLE PAK) and clarithromycin (component of VOQUEZNA TRIPLE PAK), and may range in severity from mild diarrhea to fatal colitis. Treatment with antibacterial agents alters the normal flora of the colon leading to overgrowth of *Clostridioides difficile (C. difficile)*.

C. difficile produces toxins A and B which contribute to the development of CDAD. Hypertoxin-producing strains of *C. difficile* cause increased morbidity and mortality, as these infections can be refractory to antimicrobial therapy and may require colectomy. CDAD must be considered in all patients who present with diarrhea following antibacterial use. Careful medical history is necessary since CDAD has been reported to occur over two months after the administration of antibacterial agents.

If CDAD is confirmed, VOQUEZNA TRIPLE PAK and VOQUEZNA DUAL PAK should be discontinued. Appropriate fluid and electrolyte management, protein supplementation, antibacterial treatment of *C. difficile*, and surgical evaluation should be instituted as clinically indicated.

Rash in Patients with Mononucleosis

A high percentage of patients with mononucleosis who receive amoxicillin (a component of VOQUEZNA TRIPLE PAK and VOQUEZNA DUAL PAK) develop an erythematous skin rash. Avoid use of VOQUEZNA TRIPLE PAK and VOQUEZNA DUAL PAK in patients with mononucleosis.

Interactions with Diagnostic Investigations for Neuroendocrine Tumors

Serum chromogranin A (CgA) levels increase secondary to drug-induced decreases in gastric acidity. The increased CgA level may cause false positive results in diagnostic investigations for neuroendocrine tumors. Assess CgA levels at least 14 days after VOQUEZNA TRIPLE PAK and VOQUEZNA DUAL PAK treatment and consider repeating the test if initial CgA levels are high *[see Drug Interactions (7)].*

Development of Drug-Resistant Bacteria

Prescribing VOQUEZNA TRIPLE PAK or VOQUEZNA DUAL PAK in the absence of a proven or strongly suspected bacterial infection or prophylactic indication is unlikely to provide benefit to the patient, and increases the risk of the development of drug-resistant bacteria.

5.2. Additional Warnings and Precautions for VOQUEZNA TRIPLE PAK Due to the Clarithromycin Component

QT Prolongation

Clarithromycin (a component of VOQUEZNA TRIPLE PAK) has been associated with prolongation of the QT interval and infrequent cases of arrhythmia. Cases of *torsades de pointes* have been spontaneously reported during postmarketing surveillance in patients receiving clarithromycin. Fatalities have been reported.

Avoid VOQUEZNA TRIPLE PAK in the following patients:

- Patients with known prolongation of QT interval, ventricular cardiac arrhythmia, including *torsades de pointes.*
- Patients receiving drugs known to prolong the QT interval (e.g., pimozide).
- Patients with ongoing proarrhythmic conditions such as uncorrected hypokalemia or hypomagnesemia, clinically significant bradycardia and in patients receiving Class IA (e.g., quinidine, procainamide, disopyramide) or Class III (dofetilide, amiodarone, sotalol) antiarrhythmic agents.

Elderly patients may be more susceptible to drug-associated effects on the QT interval *[see Use in Specific Populations (8.5)].*

Hepatotoxicity

Hepatic dysfunction, including increased liver enzymes, and hepatocellular and/or cholestatic hepatitis, with or without jaundice, has been reported with clarithromycin (a component of VOQUEZNA TRIPLE PAK). This hepatic dysfunction may be severe and is usually reversible. In some instances, hepatic failure with fatal outcome has been reported and generally has been associated with serious underlying diseases and/or concomitant medications. Symptoms of hepatitis can include anorexia, jaundice, dark urine, pruritus, or tender abdomen.

Discontinue VOQUEZNA TRIPLE PAK immediately if signs and symptoms of hepatitis occur.

Serious Adverse Reactions Due to Concomitant Use of Clarithromycin with Other Drugs

Drugs metabolized by CYP3A4

Serious adverse reactions have been reported in patients taking clarithromycin (a component of VOQUEZNA TRIPLE PAK) concomitantly with CYP3A4 substrates. These include colchicine toxicity with colchicine; markedly increased transaminases with lomitapide; rhabdomyolysis with simvastatin, lovastatin, and atorvastatin; hypoglycemia and cardiac arrhythmias (e.g., *torsades de pointes*) with disopyramide; and hypotension and acute kidney injury with calcium channel blockers metabolized by CYP3A4 (e.g., verapamil, amlodipine, diltiazem, nifedipine). Most reports of acute kidney injury with calcium channel blockers metabolized by CYP3A4 involved elderly patients 65 years of age or older *[see Contraindications (4.2) and Drug Interactions (7)].*

Colchicine

Life-threatening and fatal drug interactions have been reported in patients treated with clarithromycin (a component of VOQUEZNA TRIPLE PAK) and colchicine. If co-administration of VOQUEZNA TRIPLE PAK and colchicine is necessary in patients with normal renal and hepatic function, reduce the dose of colchicine. Monitor patients for clinical symptoms of colchicine toxicity. Concomitant

administration of VOQUEZNA TRIPLE PAK and colchicine is contraindicated in patients with renal or hepatic impairment *[see Contraindications (4.2) and Drug Interactions (7)].*

Lomitapide

Concomitant use of VOQUEZNA TRIPLE PAK with lomitapide may increase the risk of elevation in transaminases due to the clarithromycin component. Concomitant use of VOQUEZNA TRIPLE PAK with lomitapide is contraindicated *[see Contraindications (4.2) and Drug Interactions (7)].* If treatment with VOQUEZNA TRIPLE PAK cannot be avoided, therapy with lomitapide must be suspended during the course of treatment.

HMG-CoA Reductase Inhibitors (statins)

Concomitant use of VOQUEZNA TRIPLE PAK with lovastatin or simvastatin may increase these drug's plasma concentrations due to the clarithromycin component, which may increase the risk of myopathy, including rhabdomyolysis. Cases of rhabdomyolysis have been reported in patients treated concomitantly with clarithromycin (a component of VOQUEZNA TRIPLE PAK) and lovastatin or simvastatin. Concomitant use of VOQUEZNA TRIPLE PAK with lovastatin or simvastatin is contraindicated *[see Contraindications (4.2)]*. If treatment with VOQUEZNA TRIPLE PAK cannot be avoided, therapy with lovastatin or simvastatin must be suspended during the course of treatment. Exercise caution when prescribing VOQUEZNA TRIPLE PAK with atorvastatin or pravastatin *[see Drug Interactions (7)]*.

Hypoglycemic Agents/Insulin

Concomitant use of VOQUEZNA TRIPLE PAK, and hypoglycemic agents (such as nateglinide, pioglitazone, repaglinide and rosiglitazone) and/or insulin can result in significant hypoglycemia due to the clarithromycin component. Carefully monitor glucose levels when these drugs are used concomitantly with VOQUEZNA TRIPLE PAK *[see Drug Interactions (7)].*

Quetiapine

Concomitant use of VOQUEZNA TRIPLE PAK with quetiapine could result in somnolence, orthostatic hypotension, altered state of consciousness, neuroleptic malignant syndrome, and QT prolongation due to the clarithromycin component. Refer to quetiapine prescribing information for recommended dosage reduction if co-administered with VOQUEZNA TRIPLE PAK *[see Drug Interactions (7)].*

Warfarin

There is a risk of serious hemorrhage and significant elevations in the international normalized ratio (INR) and prothrombin time when clarithromycin (a component of VOQUEZNA TRIPLE PAK) is used concomitantly with warfarin. Monitor INR and prothrombin times frequently when warfarin is used concomitantly with VOQUEZNA TRIPLE PAK.

Benzodiazepines

Increased sedation and prolongation of sedation have been reported with concomitant administration when clarithromycin (a component of VOQUEZNA TRIPLE PAK), and triazolobenzodiazepines, such as triazolam and midazolam. Closely monitor patients for signs or symptoms of increased or prolonged central nervous system effects when benzodiazepines such astriazolam or midazolam are used concomitantly with VOQUEZNA TRIPLE PAK *[see Drug Interactions (7)].*

Embryo-Fetal Toxicity with Use of VOQUEZNA TRIPLE PAK

Based on findings from animal studies and human observational studies in pregnant women with use of clarithromycin, VOQUEZNA TRIPLE PAK is not recommended for use in pregnant women except

in clinical circumstances where no alternative therapy is appropriate. If VOQUEZNA TRIPLE PAK is used during pregnancy, or if pregnancy occurs while the patient is taking this drug, advise the patient of the potential risk to the fetus. Clarithromycin demonstrated adverse effects on pregnancy outcome and/or embryo fetal development, in pregnant animals administered oral clarithromycin. Observational studies in pregnant women also demonstrated adverse effects on pregnancy outcomes, including an increased risk of miscarriage and in some studies an increased incidence of fetal malformations *[see Use in Specific Populations (8.1)].*

Exacerbation of Myasthenia Gravis

Exacerbation of symptoms of myasthenia gravis and new onset of symptoms of myasthenic syndrome has been reported in patients receiving clarithromycin therapy (a component of VOQUEZNA TRIPLE PAK). Monitor patients for symptoms.

6. ADVERSE REACTIONS

The following serious adverse reactions are described below and elsewhere in labeling:

- Hypersensitivity Reactions *[see Warnings and Precautions (5.1)]*
- *Clostridioides difficile*-Associated Diarrhea *[see Warnings and Precautions (5.1)]*
- QT Prolongation *[see Warnings and Precautions (5.2)]*
- Hepatotoxicity *[see Warnings and Precautions (5.2)]*
- Serious Adverse Reactions Due to Concomitant Use with Other Drugs *[see Warnings and Precautions (5.2)]*
- Exacerbation of Myasthenia Gravis *[see Warnings and Precautions (5.2)]*

6.1. Clinical Trials Experience

Because clinical trials are conducted under widely varying conditions, adverse reaction rates observed in the clinical trials of a drug cannot be directly compared to rates in the clinical trials of another drug and may not reflect the rates observed in practice.

Adverse Reactions with VOQUEZNA TRIPLE PAK and VOQUEZNA DUAL PAK

The safety of VOQUEZNA TRIPLE PAK was evaluated in 675 adult patients (aged 20 to 82 years) in clinical trials in the United States, Europe and Japan and VOQUEZNA DUAL PAK was evaluated in 348 adult patients (aged 20 to 80 years) in a clinical trial in the United States and Europe. All the patients were screened and found to be positive for *H. pylori* infection.

The safety of VOQUEZNA TRIPLE PAK and VOQUEZNA DUAL PAK was evaluated in a randomized, controlled, double-blind triple therapy/open-label dual therapy study conducted in the United States and Europe in treatment-naïve *H. pylori*-positive adult patients. Patients were randomized 1:1:1 to vonoprazan 20 mg twice daily plus amoxicillin 1,000 mg twice daily plus clarithromycin 500 mg twice daily (VOQUEZNA TRIPLE PAK) or vonoprazan 20 mg twice daily plus amoxicillin 1,000 mg three times daily (VOQUEZNA DUAL PAK) or lansoprazole 30 mg twice daily plus amoxicillin 1,000 mg twice daily plus clarithromycin 500 mg twice daily (LAC) administered for 14 consecutive days. A total of 346 patients received VOQUEZNA TRIPLE PAK in the study, 348 received VOQUEZNA DUAL PAK and 345 received LAC. These patients had a mean age of 51 years (range 20 to 87 years); 62.2% were female, 89.3% were White, 7.4% Black or African American, 1.5% were Asian and 1.8% were others with 72.5% non-Hispanic or Latino.

Adverse Reactions Leading to Discontinuation

Treatment discontinuation due to an adverse reaction occurred in 2.3% (8/346) of the VOQUEZNA TRIPLE PAK-treated patients, 0.9% (3/348) of the VOQUEZNA DUAL PAK-treated patients and 1.2% (4/345) of the LAC-treated patients. The most common adverse reactions leading to discontinuation of VOQUEZNA TRIPLE PAK were diarrhea (0.6%) and hypertension (0.6%) and the most common adverse reaction leading to discontinuation of VOQUEZNA DUAL PAK was rash (0.6%).

Most Common Adverse Reactions

The adverse reactions occurring in ≥2% of patients are described in Table 1.

Table 1: Adverse Reactions Occurring in ≥2% of Adult Patients Receiving VOQUEZNA DUAL PAK or VOQUEZNA TRIPLE PAK

Adverse Reactions	VOQUEZNA DUAL PAK (N=348) n (%)	VOQUEZNA TRIPLE PAK (N=346) n (%)	LAC (N=345) n (%)
Diarrhea	18 (5.2)	14 (4.0)	33 (9.6)
Dysgeusia[a]	2 (0.6)	16 (4.6)	21 (6.1)
Vulvovaginal candidiasis[b]	7 (2.0)	11 (3.2)	5 (1.4)
Abdominal pain[c]	9 (2.6)	8 (2.3)	10 (2.9)
Headache	5 (1.4)	9 (2.6)	5 (1.4)
Hypertension[d]	4 (1.1)	7 (2.0)	3 (0.9)
Nasopharyngitis	7 (2.0)	1 (0.3)	3 (0.9)

[a] Dysgeusia also includes taste disorder.

[b] Vulvovaginal candidiasis includes: urogenital infection fungal, vulvovaginal candidiasis, vulvovaginal mycotic infection, vulvovaginal pruritus, pruritus genital, genital infection fungal.

[c] Abdominal pain includes: abdominal discomfort, abdominal pain, abdominal pain lower, abdominal pain upper.

[d] Hypertension also includes blood pressure increased.

This study was not designed to evaluate meaningful comparisons of the incidence of adverse reactions in the VOQUEZNA DUAL PAK, VOQUEZNA TRIPLE PAK, and LAC treatment groups.

Other Adverse Reactions

Other adverse reactions occurring in <2% of patients with VOQUEZNA TRIPLE PAK or VOQUEZNA DUAL PAK are listed below by body system:

Blood and lymphatic system disorders: anemia, leukocytosis, leukopenia, neutropenia.

Cardiac disorders: QT prolongation, tachycardia.

Eye disorders: orbital edema.

Gastrointestinal disorders: abdominal distension, constipation, dry mouth, duodenal polyp, duodenal ulcer, dyspepsia, flatulence, gastric ulcer, gastroesophageal reflux disease, hematochezia, large intestine polyp, nausea, rectal polyp, stomatitis, tongue discomfort, vomiting.

General disorders and administration site conditions: fatigue, pyrexia.

Immune system disorders: drug hypersensitivity.

Infections and infestations: anal fungal infection, gastrointestinal viral infection, oral fungal infection, pneumonia, tongue fungal infection, upper respiratory tract infection, urinary tract infection, viral infection.

Investigations: liver function test abnormal.

Metabolism and nutrition disorders: decreased appetite.

Musculoskeletal system: bone fracture.

Nervous system disorders: ageusia, dizziness, tension headache.

Psychiatric disorders: anxiety, depression, insomnia.

Renal and urinary disorders: renal hypertrophy, tubulointerstitial nephritis.

Reproductive system and breast disorders: vaginal discharge.

Respiratory, thoracic and mediastinal disorders: cough, nasal polyps, oropharyngeal pain.

Skin and subcutaneous tissue disorders: dermatitis, dry skin, rash.

6.2. Postmarketing Experience with Components of VOQUEZNA TRIPLE PAK and VOQUEZNA DUAL PAK

The following adverse reactions have been identified during post-approval use of vonoprazan (outside of the United States), amoxicillin or clarithromycin (all used separately). Because these reactions are reported voluntarily from a population of uncertain size, it is not always possible to reliably estimate their frequency or establish a causal relationship to drug exposure.

<u>Vonoprazan</u>

Immune system disorders: anaphylactic shock, urticaria, drug eruption *[see Contraindications (4.1)].*

Hepatobiliary disorders: hepatic injury, hepatic failure, jaundice.

Skin and subcutaneous tissue disorders: erythema multiforme, SJS, TEN *[see Warnings and Precautions (5.1)]*.

<u>Amoxicillin</u>

Infections and infestations: mucocutaneous candidiasis.

Gastrointestinal: black hairy tongue, and hemorrhagic/pseudomembranous colitis. Onset of pseudomembranous colitis symptoms may occur during or after antibacterial treatment *[see Warnings and Precautions (5.1)].*

Hypersensitivity reactions: anaphylaxis *[see Contraindications (4.1), Warnings and Precautions (5.1)].* Serum sickness–like reactions, erythematous maculopapular rashes, erythema multiforme, exfoliative dermatitis, hypersensitivity vasculitis, and urticaria have been reported.

Renal: crystalluria has been reported *[see Overdosage (10)].*

Hemic and lymphatic systems: hemolytic anemia, thrombocytopenia, thrombocytopenic purpura, eosinophilia, and agranulocytosis have been reported during therapy with penicillins. These reactions are usually reversible on discontinuation of therapy and are believed to be hypersensitivity phenomena.

Central nervous system: reversible hyperactivity, agitation, confusion, convulsions, aseptic meningitis, and behavioral changes have been rarely reported.

Miscellaneous: tooth discoloration (brown, yellow, or gray staining) has been reported. Most reports occurred in pediatric patients. Discoloration was reduced or eliminated with brushing or dental cleaning in most cases.

Skin and subcutaneous tissue disorders: TEN, SJS, DRESS, and AGEP *[see Warnings and Precautions (5.1)]*.

Clarithromycin

Blood and lymphatic system: thrombocytopenia, agranulocytosis.

Cardiac: ventricular arrhythmia, *torsades de pointes.*

Ear and labyrinth: deafness was reported chiefly in elderly women and was usually reversible.

Gastrointestinal: pancreatitis acute, tongue discoloration, tooth discoloration was reported and was usually reversible with professional cleaning upon discontinuation of the drug.

Hepatobiliary: hepatic failure, jaundice hepatocellular. Adverse reactions related to hepatic dysfunction have been reported with clarithromycin *[see Warnings and Precautions (5.2)].*

Infections and infestations: pseudomembranous colitis *[see Warnings and Precautions (5.2)].*

Immune system: anaphylactic reactions, angioedema.

Investigations: prothrombin time prolonged, white blood cell count decreased, INR increased. Abnormal urine color has been reported, associated with hepatic failure.

Metabolism and nutrition: hypoglycemia has been reported in patients taking oral hypoglycemic agents or insulin.

Musculoskeletal and connective tissue: myopathy rhabdomyolysis was reported and in some of the reports, clarithromycin was administered concomitantly with statins, fibrates, colchicine or allopurinol *[see Contraindications (4.2) and Warnings and Precautions (5.2)].*

Nervous system: parosmia, anosmia, paresthesia and convulsions.

Psychiatric: abnormal behavior, confusional state, depersonalization, disorientation, hallucination, manic behavior, abnormal dream, psychotic disorder. These disorders usually resolve upon discontinuation of the drug.

Renal and urinary: renal failure.

Skin and subcutaneous tissue disorders: TEN, SJS, DRESS, AGEP, Henoch-Schonlein purpura *[see Warnings and Precautions (5.1)]*, acne.

Vascular: hemorrhage.

7. DRUG INTERACTIONS

Collated drug interaction information for the individual components in VOQUEZNA TRIPLE PAK and VOQUEZNA DUAL PAK is summarized below. Drug interaction studies with VOQUEZNA TRIPLE PAK or VOQUEZNA DUAL PAK have not been conducted.

These recommendations are based on either drug interaction trials or predicted interactions due to the expected magnitude of interaction and potential for serious adverse reactions or loss of efficacy *[see Clinical Pharmacology (12.3)]*.

Clarithromycin (a component of VOQUEZNA TRIPLE PAK) is a strong CYP3A inhibitor. Concomitant use of VOQUEZNA TRIPLE PAK with a drug(s) primarily metabolized by CYP3A may cause

elevations in CYP3A substrate drug's concentrations that could increase or prolong both therapeutic and adverse effects of the concomitant drug.

Table 2: Effects of Other Drugs on VOQUEZNA TRIPLE PAK

**Strong or Moderate CYP3A Inducers**	
Clinical Effect	Vonoprazan and clarithromycin are CYP3A substrates. Strong or moderate CYP3A inducers may decrease exposure of vonoprazan and clarithromycin *[see Clinical Pharmacology (12.3)]*, which may reduce the effectiveness of VOQUEZNA TRIPLE PAK.
Prevention or Management	Avoid concomitant use with VOQUEZNA TRIPLE PAK.
**Probenecid**	
Clinical Effect	Amoxicillin undergoes tubular secretion. Probenecid may increase amoxicillin exposure by blocking its renal tubular secretion, which may increase the risk of VOQUEZNA TRIPLE PAK adverse reactions.
Prevention or Management	Closely monitor for signs or symptoms of increased or prolonged adverse reactions associated with amoxicillin when used with VOQUEZNA TRIPLE PAK.
**Allopurinol**	
Clinical Effect	Increase in the incidence of rashes is reported in patients receiving both allopurinol and amoxicillin together compared to patients receiving amoxicillin alone. It is not known whether this potentiation of amoxicillin rashes is due to allopurinol or the hyperuricemia present in these patients.
Prevention or Management	Discontinue allopurinol at the first appearance of skin rash when used concomitantly with VOQUEZNA TRIPLE PAK.
**Omeprazole**	
Clinical Effect	Clarithromycin concentrations in the gastric tissue and mucus were increased by concomitant administration of omeprazole *[see Clinical Pharmacology (12.3)]*.
Prevention or Management	Avoid concomitant use of VOQUEZNA TRIPLE PAK with omeprazole.
**Itraconazole**	
Clinical Effect	Both clarithromycin and itraconazole are substrates and inhibitors of CYP3A, potentially leading to a bi-directional drug interaction when administered concomitantly. VOQUEZNA TRIPLE PAK's use with strong CYP3A4 inhibitors may lead to increases in clarithromycin exposure, which may increase the risk of VOQUEZNA TRIPLE PAK adverse reactions.
Prevention or Management	Patients taking itraconazole with VOQUEZNA TRIPLE PAK should be monitored closely for signs or symptoms of increased or prolonged adverse reactions associated with itraconazole and clarithromycin.

**Antivirals**		
Clinical Effect	Clarithromycin is a CYP3A4 substrate and inhibitor. Use of VOQUEZNA TRIPLE PAK with antivirals that are CYP3A substrates, inducers, or CYP3A inhibitors may potentially lead to bi-directional drug interactions leading to alterations in exposure of clarithromycin and/or CYP3A substrates, which may increase the risk of adverse reactions or loss of effectiveness. *[see Clinical Pharmacology (12.3)]*.	
Prevention or Management	Saquinavir (CYP3A substrate and inhibitor)	Use VOQUEZNA TRIPLE PAK with caution. See saquinavir prescribing information for instructions when saquinavir (with or without ritonavir) is co-administered with clarithromycin.
	Ritonavir (CYP3A inhibitor)	Use of VOQUEZNA TRIPLE PAK with ritonavir is not recommended in patients with decreased renal function.
	Etravirine (CYP3A inducer)	Avoid concomitant use with VOQUEZNA TRIPLE PAK.

Table 3: Effects of Other Drugs on VOQUEZNA DUAL PAK

**Strong or Moderate CYP3A Inducers**	
Clinical Effect	Vonoprazan is a CYP3A substrate. Strong or moderate CYP3A inducers may decrease vonoprazan exposure *[see Clinical Pharmacology (12.3)]*, which may reduce the effectiveness of VOQUEZNA DUAL PAK.
Prevention or Management	Avoid concomitant use with VOQUEZNA DUAL PAK.
**Probenecid**	
Clinical Effect	Amoxicillin undergoes tubular secretion. Probenecid may increase amoxicillin exposure by blocking its renal tubular secretion, which may increase the risk of VOQUEZNA DUAL PAK adverse reactions.
Prevention or Management	Closely monitor for signs or symptoms of increased or prolonged adverse reactions associated with amoxicillin when used with VOQUEZNA DUAL PAK.
**Allopurinol**	
Clinical Effect	Increase in the incidence of rashes is reported in patients receiving both allopurinol and amoxicillin together compared to patients receiving amoxicillin alone. It is not known whether this potentiation of amoxicillin rashes is due to allopurinol or the hyperuricemia present in these patients.
Prevention or Management	Discontinue allopurinol at the first appearance of skin rash when used concomitantly with VOQUEZNA DUAL PAK.

Table 4: Effects of VOQUEZNA TRIPLE PAK on Other Drugs

**Drugs Dependent on Gastric pH for Absorption**		
**Antiretrovirals**		
Clinical Effect	Vonoprazan reduces intragastric acidity *[see Clinical Pharmacology (12.2)]*, which may alter the absorption of antiretroviral drugs leading to changes in their safety and/or effectiveness.	
Prevention or Management	Rilpivirine-containing Products	Concomitant use with VOQUEZNA TRIPLE PAK is contraindicated.
	Atazanavir	Avoid concomitant use with VOQUEZNA TRIPLE PAK.
	Nelfinavir	
	Other Antiretroviral Drugs	See the prescribing information of other antiretroviral drugs dependent on gastric pH for absorption prior to concomitant use with VOQUEZNA TRIPLE PAK.
**Other Drugs (e.g., iron salts, erlotinib, dasatinib, nilotinib, mycophenolate mofetil, ketoconazole/itraconazole)**		
Clinical Effect	Vonoprazan reduces intragastric acidity *[see Clinical Pharmacology (12.2)]*, which may decrease the absorption of drugs reducing their effectiveness.	
Prevention or Management	See the prescribing information for other drugs dependent on gastric pH for absorption.	
**Certain CYP3A Substrates where minimal concentration changes may lead to serious toxicities**		
Clinical Effect	Clarithromycin is a strong CYP3A inhibitor.<br>Vonoprazan is a weak CYP3A inhibitor *[see Clinical Pharmacology (12.3)]*.<br>Clarithromycin and vonoprazan may increase exposure of CYP3A4 substrates, which may increase the risk of adverse reactions related to these substrates.<br>There have been spontaneous or published reports of CYP3A based interactions of clarithromycin with tacrolimus and cyclosporine.	
Prevention or Management	Immunosuppressants: Tacrolimus, cyclosporine.	Frequent monitoring for concentrations and/or adverse reactions related to the substrate drugs when used with VOQUEZNA TRIPLE PAK. Dosage reduction of substrate drugs may be needed.<br>See prescribing information for the relevant substrate drugs.
**CYP2C19 Substrates (e.g., clopidogrel, citalopram, cilostazol)**		
Clinical Effect	Vonoprazan is a CYP2C19 inhibitor *[see Clinical Pharmacology (12.3)]*. Vonoprazan may reduce plasma concentrations of the active metabolite of	

	clopidogrel and may cause reduction in platelet inhibition. Vonoprazan may increase exposure of CYP2C19 substrate drugs (e.g., citalopram, cilostazol).	
Prevention or Management	Clopidogrel	Carefully monitor the efficacy of clopidogrel and may consider alternative anti-platelet therapy.
	Citaprolam and Cilostazol	Carefully monitor patients for adverse reactions associated with citaprolam and cilostazol. See the prescribing information for dosage adjustments.
**Oral Anticoagulants**		
Clinical Effect	Abnormal prolongation of prothrombin time (increased INR) has been reported in patients receiving amoxicillin and oral anticoagulants.	
Prevention or Management	Appropriate monitoring should be undertaken when anticoagulants are prescribed concurrently. Adjustments in the dose of oral anticoagulants may be necessary to maintain the desired level of anticoagulation.	
**Chromogranin A (CgA) Test for Neuroendocrine Tumors**		
Clinical Effect	Vonoprazan reduces intragastric acidity *[see Clinical Pharmacology (12.2)]*, which increases chromogranin A (CgA) levels and may cause false positive results in diagnostic investigations for neuroendocrine tumors.	
Prevention or Management	Assess CgA levels at least 14 days after VOQUEZNA TRIPLE PAK treatment and repeat the test if initial CgA levels are high. If serial tests are performed (e.g., for monitoring), use the same commercial laboratory for testing, as reference ranges between tests may vary.	
**Glucose Tests**		
Clinical Effect	Amoxicillin is primarily excreted in the urine *[see Clinical Pharmacology (12.3)]*. High urine concentrations of ampicillin or amoxicillin may cause false-positive results when using glucose tests based on the Benedict's copper reduction reaction that determines the amount of reducing substances like glucose in the urine.	
Prevention or Management	Use a test based on enzymatic glucose oxidase reactions when testing for glucose in the urine of patients treated with VOQUEZNA TRIPLE PAK.	
**Itraconazole**		
Clinical Effect	Both clarithromycin and itraconazole are substrates and inhibitors of CYP3A, potentially leading to a bi-directional drug interaction when administered concomitantly. VOQUEZNA TRIPLE PAK's use with strong CYP3A4 inhibitors may lead to increases in clarithromycin exposure, which may increase the risk of VOQUEZNA TRIPLE PAK adverse reactions.	
Prevention or Management	Patients taking itraconazole with VOQUEZNA TRIPLE PAK should be monitored closely for signs or symptoms of increased or prolonged adverse reactions associated with itraconazole and clarithromycin.	

**Antiarrhythmics**		
Clinical Effect	Clarithromycin is a strong CYP3A inhibitor. Clarithromycin may increase exposure of antiarrhythmic drugs that are CYP3A substrates, which may increase the risk of adverse reactions related to these substrates including cardiac arrhythmias (e.g., *torsades de pointes*).<br>There have been spontaneous or published reports of CYP3A based interactions of clarithromycin with disopyramide and quinidine.<br>There have been postmarketing reports of hypoglycemia with the concomitant administration of clarithromycin and disopyramide	
Prevention or Management	Disopyramide	Avoid concomitant use with VOQUEZNA TRIPLE PAK. If concomitant use is unavoidable, monitor patients for QTc prolongation and changes in blood glucose levels.
	Amiodarone	Avoid concomitant use with VOQUEZNA TRIPLE PAK. If concomitant use is unavoidable, monitor patients for QTc prolongation.
	Dofetilide	
	Procainamide	
	Sotalol	
	Quinidine	
**Colchicine**		
Clinical Effect	Clarithromycin is an inhibitor of CYP3A and the efflux transporter, P-glycoprotein (P-gp). Colchicine is a substrate of CYP3A and P-gp. Clarithromycin increases exposure of colchicine *[see Clinical Pharmacology (12.3)]*, which may increase the risk of adverse reactions related to colchicine.	
Prevention or Management	Concomitant use of colchicine with VOQUEZNA TRIPLE PAK is contraindicated in patients with renal or hepatic impairment. If co-administration of VOQUEZNA TRIPLE PAK and colchicine is necessary in patients with normal renal or hepatic function, carefully monitor patients for clinical symptoms of colchicine toxicity and refer to the colchicine prescribing information for recommendations on dosage reduction.	
**Antipsychotics**		
Clinical Effect	Clarithromycin is a strong CYP3A inhibitor. Clarithromycin may increase exposure of antipsychotic drugs that are CYP3A substrates, which may increase the risk of adverse reactions related to these substrates including the risk of somnolence, orthostatic hypotension, altered state of consciousness, neuroleptic malignant syndrome, or cardiac arrhythmias (QT prolongation, ventricular tachycardia, ventricular fibrillation, and *torsades de pointes*).	
Prevention or Management	Pimozide	Concomitant use with VOQUEZNA TRIPLE PAK is contraindicated.

	Quetiapine	Refer to quetiapine prescribing information for recommendations on dosage reduction if co-administered with CYP3A4 inhibitors such as clarithromycin.
**Tolterodine (patients deficient in CYP2D6 activity)**		
Clinical Effect	Clarithromycin is a strong CYP3A inhibitor. The primary route of metabolism for tolterodine is via CYP2D6. Clarithromycin may increase tolterodine exposure and the risk of adverse reactions related to tolterodine in patients deficient in CYP2D6 activity because tolterodine is metabolized via CYP3A in this subset of population.	
Prevention or Management	Tolterodine 1 mg twice daily is recommended in patients deficient in CYP2D6 activity (poor metabolizers) when co-administered with strong CYP3A4 inhibitors such as clarithromycin.	
**Antivirals**		
Clinical Effect	Clarithromycin is a CYP3A4 substrate and inhibitor. Use of VOQUEZNA TRIPLE PAK with antivirals that are CYP3A substrates, inducers, or CYP3A inhibitors may potentially lead to bi-directional drug interactions leading to alterations in exposure of clarithromycin and/or CYP3A substrates, which may increase the risk of adverse reactions or loss of effectiveness *[see Clinical Pharmacology (12.3)]*.	
Prevention or Management	Saquinavir (CYP3A substrate and inhibitor)	Use VOQUEZNA TRIPLE PAK with caution. See saquinavir prescribing information for instructions when saquinavir (with or without ritonavir) is co-administered with clarithromycin.
	Maraviroc (CYP3A substrate)	Use VOQUEZNA TRIPLE PAK with caution. See the prescribing information of maraviroc for dosage recommendation when given with strong CYP3A inhibitors such as clarithromycin.
	Zidovudine	Administration of VOQUEZNA TRIPLE PAK and zidovudine should be separated by at least two hours.
**Benzodiazepines**		
Clinical Effect	Clarithromycin is a strong CYP3A inhibitor. Clarithromycin may increase exposure of benzodiazepines that are CYP3A substrates, which may increase the risk of adverse reactions related to these substrates *[see Warnings and Precautions (5.2) and Clinical Pharmacology (12.3)]*.	
Prevention or Management	Midazolam	Closely monitor patients for signs or symptoms of increased or prolonged central nervous system effects s (e.g., somnolence and confusion) and refer to the CYP3A substrate prescribing information for dosage
	Alprazolam	
	Triazolam	

		adjustments when used concomitantly with VOQUEZNA TRIPLE PAK.
**Calcium Channel Blockers**		
Clinical Effect	Clarithromycin is a strong CYP3A inhibitor. Clarithromycin may increase exposure of calcium channel blockers that are CYP3A substrates, which may increase the risk of adverse reactions related to these substrates including hypotension, acute kidney injury, bradyarrhythmias, lactic acidosis, or peripheral edema.	
Prevention or Management	Verapamil	Use VOQUEZNA TRIPLE PAK with caution.
	Amlodipine	
	Diltiazem	
	Nifedipine	
**Ergot Alkaloids**		
Clinical Effect	Clarithromycin is a strong CYP3A inhibitor. Clarithromycin may increase exposure of ergot alkaloids that are CYP3A substrates, which may increase the risk of vasospasm and ischemia of the extremities and other tissues including the central nervous system *[see Contraindications (4.2)]*.	
Prevention or Management	Ergotamine	Concomitant use with VOQUEZNA TRIPLE PAK is contraindicated.
	Dihydroergotamine	
**Hypoglycemic Agents**		
Clinical Effect	Clarithromycin is a strong CYP3A inhibitor. Clarithromycin may increase exposure of hypoglycemic agents that are CYP3A substrates, which may increase the risk of hypoglycemia *[see Warnings and Precautions (5.2)]*.	
Prevention or Management	Nateglinide	Closely monitor glucose levels when used concomitantly with VOQUEZNA TRIPLE PAK.
	Pioglitazone	
	Repaglinide	
	Rosiglitazone	
	Insulin	
**Lipid-lowering Agents**		
Clinical Effect	Clarithromycin is a strong CYP3A inhibitor. Clarithromycin may increase exposure of lipid-lowering drugs that are CYP3A substrates, thereby increasing the risk of toxicities from these drugs *[see Warnings and Precautions (5.2)]*.	
	Lomitapide	

Prevention or Management	Lovastatin	Concomitant use with VOQUEZNA TRIPLE PAK is contraindicated.
	Simvastatin	
	Atorvastatin	Use VOQUEZNA TRIPLE PAK with caution. In situations where the concomitant use of VOQUEZNA TRIPLE PAK with atorvastatin or pravastatin cannot be avoided, atorvastatin dose should not exceed 20 mg daily and pravastatin dose should not exceed 40 mg daily.
	Pravastatin	
	Fluvastatin	Use of a statin that is not dependent on CYP3A metabolism (e.g., fluvastatin) can be considered. It is recommended to prescribe the lowest registered dose if concomitant use cannot be avoided.
**Phosphodiesterase Inhibitors**		
Clinical Effect	Clarithromycin is a strong CYP3A inhibitor. Clarithromycin may increase exposure of phosphodiesterase inhibitors that are CYP3A substrates, which may increase the risk of adverse reactions related to these substrates.	
Prevention or Management	Sildenafil	Avoid concomitant use with VOQUEZNA TRIPLE PAK. If concomitant use is unavoidable, see the prescribing information of the respective phosphodiesterase inhibitors for dosage recommendation when given with strong CYP3A inhibitors such as clarithromycin.
	Tadalafil	
	Vardenafil	
**Other CYP3A Based Interactions**		
Clinical Effect	Clarithromycin is a substrate and strong inhibitor of CYP3A4. Clarithromycin increases exposure of CYP3A substrates *[see Clinical Pharmacology (12.3)]*, which may increase the risk of adverse reactions related to these substrates *[see Warnings and Precautions (5.2)]*. Strong or moderate CYP3A inducers may decrease exposure of clarithromycin. There have been spontaneous or published reports of CYP3A based interactions of clarithromycin with alfentanil, methylprednisolone, cilostazol, bromocriptine, vinblastine, phenobarbital, and St. John's Wort.	
Prevention or Management	Use VOQUEZNA TRIPLE PAK with caution.	
**P-glycoprotein (P-gp) Substrates: Digoxin**		
Clinical Effect	Clarithromycin is a P-gp inhibitor. Clarithromycin may increase exposure of P-gp substrates, which may increase the risk of adverse reactions related to these substrates, including potentially fatal arrhythmias. Elevated digoxin serum concentrations in patients receiving clarithromycin and digoxin concomitantly have been reported in postmarketing surveillance. Some patients have shown clinical signs consistent with digoxin toxicity, including potentially fatal arrhythmias.	

Prevention or Management	Digoxin	Carefully monitor serum concentrations and refer to the digoxin prescribing information for dosage adjustments when used concomitantly with VOQUEZNA TRIPLE PAK.
**Drugs Metabolized by CYP450 Isoforms Other than CYP3A**		
Clinical Effect	Clarithromycin may increase exposure of drugs that are metabolized by CYP450 isoforms other than CYP3A by inhibiting their metabolism. There have been post-marketing reports of interactions of clarithromycin with drugs not thought to be metabolized by CYP3A.	
Prevention or Management	Hexobarbital	Use VOQUEZNA TRIPLE PAK with caution.
	Phenytoin	
	Valproate	
**Theophylline**		
Clinical Effect	Clarithromycin may increase exposure of theophylline (a xanthine derivative drug) *[see Clinical Pharmacology (12.3)]*, which may increase the risk of adverse reactions related to theophylline.	
Prevention or Management	Closely monitor serum theophylline concentrations in patients receiving high dosages of theophylline or with baseline concentrations in the upper therapeutic range when used concomitantly with VOQUEZNA TRIPLE PAK.	

Table 5: Effects of VOQUEZNA DUAL PAK on Other Drugs

**Drugs Dependent on Gastric pH for Absorption**		
**Antiretrovirals**		
Clinical Effect	Vonoprazan reduces intragastric acidity *[see Clinical Pharmacology (12.2)]*, which may alter the absorption of antiretroviral drugs leading to changes in their safety and/or effectiveness.	
Prevention or Management	Rilpivirine-containing Products	Concomitant use with VOQUEZNA DUAL PAK is contraindicated.
	Atazanavir	Avoid concomitant use with VOQUEZNA DUAL PAK
	Nelfinavir	
	Other Antiretroviral Drugs	See the prescribing information of other antiretroviral drugs dependent on gastric pH for absorption prior to concomitant use with VOQUEZNA DUAL PAK.
**Other Drugs (e.g., iron salts, erlotinib, dasatinib, nilotinib, mycophenolate mofetil, ketoconazole/itraconazole)**		

<table>
<tr><td>Clinical Effect</td><td colspan="2">Vonoprazan reduces intragastric acidity [see Clinical Pharmacology (12.2)], which may decrease the absorption of drugs reducing their effectiveness.</td></tr>
<tr><td>Prevention or Management</td><td colspan="2">See the prescribing information for other drugs dependent on gastric pH for absorption.</td></tr>
<tr><td colspan="3">Certain CYP3A Substrates where minimal concentration changes may lead to serious toxicities</td></tr>
<tr><td>Clinical Effect</td><td colspan="2">Vonoprazan is a weak CYP3A inhibitor [see Clinical Pharmacology (12.3)].
Vonoprazan may increase exposure of CYP3A4 substrates, which may increase the risk of adverse reactions related to these substrates.</td></tr>
<tr><td>Prevention or Management</td><td>Immunosuppressants: Tacrolimus, cyclosporine.</td><td>Frequent monitoring for concentrations and/or adverse reactions related to the substrate drugs when used with VOQUEZNA DUAL PAK. Dosage reduction of substrate drugs may be needed.
See prescribing information for the relevant substrate drugs.</td></tr>
<tr><td colspan="3">Oral Anticoagulants</td></tr>
<tr><td>Clinical Effect</td><td colspan="2">Abnormal prolongation of prothrombin time (increased INR) has been reported in patients receiving amoxicillin and oral anticoagulants.</td></tr>
<tr><td>Prevention or Management</td><td colspan="2">Appropriate monitoring should be undertaken when anticoagulants are prescribed concurrently. Adjustments in the dose of oral anticoagulants may be necessary to maintain the desired level of anticoagulation.</td></tr>
<tr><td colspan="3">CYP2C19 Substrates (e.g., clopidogrel, citalopram, cilostazol)</td></tr>
<tr><td>Clinical Effect</td><td colspan="2">Vonoprazan is a CYP2C19 inhibitor [see Clinical Pharmacology (12.3)]. Vonoprazan may reduce plasma concentrations of the active metabolite of clopidogrel and may cause reduction in platelet inhibition. Vonoprazan may increase exposure of CYP2C19 substrate drugs (e.g., citalopram, cilostazol).</td></tr>
<tr><td rowspan="2">Prevention or Management</td><td>Clopidogrel</td><td>Carefully monitor the efficacy of clopidogrel and may consider alternative anti-platelet therapy</td></tr>
<tr><td>Citaprolam and Cilostazol</td><td>Carefully monitor patients for adverse reactions associated with citaprolam and cilostazol. See the prescribing information for dosage adjustments.</td></tr>
<tr><td colspan="3">CgA Test for Neuroendocrine Tumors</td></tr>
<tr><td>Clinical Effect</td><td colspan="2">Vonoprazan reduces intragastric acidity [see Clinical Pharmacology (12.2)], which increases CgA levels and may cause false positive results in diagnostic investigations for neuroendocrine tumors.</td></tr>
<tr><td>Prevention or Management</td><td colspan="2">Assess CgA levels at least 14 days after VOQUEZNA DUAL PAK treatment and repeat the test if initial CgA levels are high. If serial tests are performed (e.g., for</td></tr>
</table>

	monitoring), use the same commercial laboratory for testing, as reference ranges between tests may vary.
**Glucose Tests**	
Clinical Effect	Amoxicillin is primarily excreted in the urine *[see Clinical Pharmacology (12.3)]*. High urine concentrations of ampicillin or amoxicillin may cause false-positive results when using glucose tests based on the Benedict's copper reduction reaction that determines the amount of reducing substances like glucose in the urine.
Prevention or Management	Use a test based on enzymatic glucose oxidase reactions when testing for glucose in the urine of patients treated with VOQUEZNA DUAL PAK.

8. USE IN SPECIFIC POPULATIONS

8.1. Pregnancy

Risk Summary

VOQUEZNA TRIPLE PAK

Based on findings from animal studies and observational studies in pregnant women with use of clarithromycin, use of VOQUEZNA TRIPLE PAK is not recommended in pregnant women except in clinical circumstances where no alternative therapy is appropriate. There are no adequate and well-controlled studies of VOQUEZNA TRIPLE PAK in pregnant women to evaluate for drug-associated risks of major birth defects, miscarriage, or other adverse maternal or fetal outcomes. If VOQUEZNA TRIPLE PAK is used during pregnancy, advise pregnant women of the potential risk to a fetus.

No reproductive and developmental toxicity studies with the combination of vonoprazan, amoxicillin, and/or clarithromycin were conducted.

VOQUEZNA DUAL PAK

There are no adequate and well-controlled studies of VOQUEZNA DUAL PAK in pregnant women to evaluate for drug-associated risks of major birth defects, miscarriage, or other adverse maternal or fetal outcomes.

Individual Components of VOQUEZNA TRIPLE PAK and VOQUEZNA DUAL PAK

Clarithromycin:

Published observational studies in pregnant women have demonstrated adverse effects on pregnancy outcomes, including an increased risk of miscarriage and in some studies an increased incidence of fetal malformations *(see Data)*. In animal reproduction studies, administration of oral clarithromycin to pregnant mice, rats, rabbits, and monkeys during the period of organogenesis produced malformations in rats (cardiovascular anomalies) and mice (cleft palate) at clinically relevant doses. Fetal effects in mice, rats, and monkeys (e.g., reduced fetal survival, body weight, body weight gain) and implantation losses in rabbits were generally considered to be secondary to maternal toxicity *(see Data)*.

Vonoprazan:

Available data from pharmacovigilance reports with vonoprazan use in pregnant women are not sufficient to evaluate for a drug-associated risk for major birth defects, miscarriage or other adverse maternal or fetal outcomes.

In pregnant rats, no adverse effects were noted after oral administration of vonoprazan during organogenesis at approximately 27 times the maximum recommended human dose (MRHD) based on AUC exposure comparisons.

In a pre- and postnatal development (PPND) study, pups from dams orally administered vonoprazan during organogenesis and through lactation, exhibited liver discoloration, which in follow-up mechanistic animal studies was associated with necrosis, fibrosis and hemorrhage at a dose approximately 22 times the MRHD based on AUC comparisons which were likely attributable to exposure during lactation *[see Use in Specific Populations (8.2)]*. These effects were not observed at the next lower dose in this study, which was approximately equal to the MRHD based on AUC comparison, however they were seen at clinically relevant exposures in dose range finding studies in rats *(see Data)*.

Amoxicillin:

Available data from published epidemiologic studies and pharmacovigilance case reports over several decades with amoxicillin use have not established drug-associated risks of major birth defects, miscarriage, or adverse maternal or fetal outcomes. Reproduction studies with amoxicillin have been performed in mice and rats (5 and 10 times the human dose 2 g human dose for mice and rats, respectively, 3 and 6 times the 3 g human dose for mice and rats, respectively). There was no evidence of harm to the fetus due to amoxicillin.

The estimated background risks of major birth defects and miscarriage for the indicated population are unknown. All pregnancies have a background risk of birth defect, loss, or other adverse outcomes. In the US general population, the estimated background risk of major birth defects and miscarriage in clinically recognized pregnancies is 2% to 4% and 15% to 20%, respectively.

Report pregnancies to the Phathom Pharmaceuticals, Inc. Adverse Event reporting line at 1-800-775-PHAT (7428).

<u>*Data*</u>

Human Data

Clarithromycin:

Available data from prospective and retrospective observational studies with clarithromycin use in pregnant women demonstrate an increased risk of miscarriage. Data from these same studies regarding major congenital malformations are inconsistent, with some studies reporting an increased risk (atrioventricular septal defects, genital malformations, orofacial clefts) and others finding no difference between those exposed to clarithromycin and those exposed to nonteratogenic controls. Available studies have methodologic limitations, including small sample size, under-capture of non-live births, exposure misclassification and inconsistent comparator groups.

Animal Data

Clarithromycin:

Animal reproduction studies were conducted in mice, rats, rabbits, and monkeys with oral and intravenously administered clarithromycin. In pregnant mice, clarithromycin was administered during organogenesis (gestation day [GD] 6 to 15) at oral doses of 15, 60, 250, 500, or 1000 mg/kg/day. Reduced body weight observed in dams at 1000 mg/kg/day (3 times the MRHD based on BSA comparison) resulted in reduced survival and body weight of the fetuses. At ≥ 500 mg/kg/day, increases in the incidence of post-implantation loss and cleft palate in the fetuses were observed. No

adverse developmental effects were observed in mice at ≤ 250 mg/kg/day (≤ 1 times MRHD based on BSA comparison).

In pregnant Sprague Dawley rats, clarithromycin was administered during organogenesis (GD 6 to 15) at oral doses of 15, 50, or 150 mg/kg/day. Reductions in body weight and food consumption was observed in dams at 150 mg/kg/day. Increased resorptions and reduced body weight of the fetuses at this dose were considered secondary to maternal toxicity. Additionally, at 150 mg/kg/day (1 times MRHD based on BSA comparison), a low incidence of cardiovascular anomalies (complete situs inversus, undivided truncus, IV septal defect) was observed in the fetuses. Clarithromycin did not cause adverse developmental effects in rats at 50 mg/kg/day (0.3 times MRHD based on BSA comparison). Intravenous dosing of clarithromycin during organogenesis in rats (GD 6 to 15) at 15, 50, or 160 mg/kg/day was associated with maternal toxicity (reduced body weight, body-weight gain, and food consumption) at 160 mg/kg/day but no evidence of adverse developmental effects at any dose (≤ 1 times MRHD based on BSA comparison).

In pregnant Wistar rats, clarithromycin was administered during organogenesis (GD 7 to 17) at oral doses of 10, 40, or 160 mg/kg/day. Reduced body weight and food consumption were observed in dams at 160 mg/kg/day but there was no evidence of adverse developmental effects at any dose (≤ 1 times MRHD based on surface BSA comparison).

In pregnant rabbits, clarithromycin administered during organogenesis (GD 6 to 18) at oral doses of 10, 35, or 125 mg/kg/day resulted in reduced maternal food consumption and decreased body weight at the highest dose, with no evidence of any adverse developmental effects at any dose (≤ 2 times MRHD based on BSA comparison). Intravenously administered clarithromycin to pregnant rabbits during organogenesis (GD 6 to 18) in rabbits at 20, 40, 80, or 160 mg/kg/day (≥ 0.3 times MRHD based on BSA comparison) resulted in maternal toxicity and implantation losses at all doses.

In pregnant monkeys, clarithromycin was administered (GD 20 to 50) at oral doses of 35 or 70 mg/kg/day. Dose-dependent emesis, poor appetite, fecal changes, and reduced body weight were observed in dams at all doses (≥ 0.5 times MRHD based on BSA comparison). Growth retardation in 1 fetus at 70 mg/kg/day was considered secondary to maternal toxicity. There was no evidence of primary drug related adverse developmental effects at any dose tested.

In a reproductive toxicology study in rats administered oral clarithromycin late in gestation through lactation (GD 17 to post-natal day 21) at doses of 10, 40, or 160 mg/kg/day (≤ 1 times MRHD based on BSA comparison), reductions in maternal body weight and food consumption were observed at 160 mg/kg/day. Reduced body-weight gain observed in offspring at 160 mg/kg/day was considered secondary to maternal toxicity. No adverse developmental effects were observed with clarithromycin at any dose tested.

Vonoprazan:

Pregnant rats were orally administered vonoprazan at doses of 30, 100 or 300 mg/kg/day (7, 27, 130 times the MRHD based on AUC comparison at the same doses from unmated female rats from separate studies) during the period of organogenesis from gestation Day 6 to 17. During maternal dosing, one high-dose female died and decreased body weight and food consumption occurred at the middle and highest doses. No embryo-fetal lethality was observed but decreased fetal body weight was observed in the highest dose group. Fetal abnormalities were limited to the 300 mg/kg/day dose group and included ventricular septal defect and mal-positioned subclavian artery in fetuses in a majority (15/19) of litters, as well as tail abnormalities, and small anal opening. No adverse embryo-fetal effects were observed at the 100 mg/kg/day.

Pregnant rabbits were orally administered vonoprazan at doses of 3, 10, or 30 mg/kg/day (0.04, 1.5, 10 times the MRHD based on AUC comparison) during the period of organogenesis from gestation Day 6 to 18. Two animals aborted at the highest dose and decreased body weight and food consumption occurred at the mid and high doses. No embryo-fetal mortality or toxicity occurred. There were no external, visceral or skeletal abnormalities.

In a PPND study, pregnant female rats were orally administered vonoprazan at doses of 1, 3, 10, or 100 mg/kg/day (0.01, 0.18, 1.1, 22 times the MRHD based on AUC comparison) from GD 6 to lactation day (LD) 21. Decreased body weight gain and food consumption were present in dams at the highest dose during lactation. Decreased body weight gain compared to controls was observed in offspring from dams in the high dose group. Liver discoloration occurred in offspring from the high dose group at LD 4 but was not present in animals examined after weaning. Similarly, in dose range finding studies in rats and follow-up mechanistic animal studies, the liver discoloration was observed and characterized as necrosis, fibrosis and hemorrhage at equal to or greater than clinically relevant exposures based on AUC comparisons. The mechanistic studies further demonstrated the effect was likely attributable to vonoprazan exposure during lactation *[see Use in Specific Populations (8.2)]*. The clinical relevance of the liver findings is uncertain.

Exposure margins from vonoprazan between the animal and clinical studies for vonoprazan, amoxicillin and clarithromycin used in combination may be lower due to increased vonoprazan exposure from concomitant use with clarithromycin in patients *[see Clinical Pharmacology (12.3)].*

Amoxicillin:

Available data from published epidemiologic studies and pharmacovigilance case reports over several decades with amoxicillin use have not established drug-associated risks of major birth defects, miscarriage, or other adverse maternal or fetal outcomes. Animal reproduction studies with amoxicillin have been performed in mice and rats, at doses up 2,000 mg/kg (5 and 10 times the 2 g human dose for mice and rats, respectively, 3 and 6 times the 3 g human dose for mice and rats, respectively, based on BSA comparison). There was no evidence of harm to the fetus due to amoxicillin.

8.2. Lactation

Risk Summary

There are no data regarding the presence of vonoprazan in human milk, the effects on the breastfed infant or the effects on milk production. Vonoprazan and its metabolites are present in rat milk. Liver injury occurred in offspring from pregnant and lactating rats administered oral vonoprazan at AUC exposures approximately equal to and greater than the MRHD *(see Data).* When a drug is present in animal milk, it is likely that the drug will be present in human milk. Because of the potential risk of adverse liver effects shown in animal studies with vonoprazan, a woman should pump and discard human milk for the duration of VOQUEZNA TRIPLE PAK or VOQUEZNA DUAL PAK therapy, and for 2 days after therapy ends, and feed her infant stored human milk (collected prior to therapy) or formula.

Based on data from a published lactation study, clarithromycin and its active metabolite 14-OH clarithromycin are present in human milk at less than 2% of the maternal weight-adjusted dose *(see Data)*. In a separate observational study of lactating women exposed to clarithromycin, reported adverse effects on breast-fed children (rash, diarrhea, loss of appetite, somnolence) were comparable to amoxicillin. No data are available to assess the effects of clarithromycin or 14-OH clarithromycin on milk production.

Data from published clinical lactation study reports that amoxicillin is present in human milk. There are no data on the effects of amoxicillin on milk production.

Data

Human Data

Clarithromycin:

Serum and milk samples were obtained after 3 days of treatment, at steady state, from one published study of 12 lactating women who were taking clarithromycin 250 mg orally twice daily. Based on data from this study, and assuming milk consumption of 150 mL/kg/day, an exclusively human milk fed infant would receive an estimated average of 136 mcg/kg/day of clarithromycin and its active metabolite, with this maternal dosage regimen. This is less than 2% of the maternal weight-adjusted dose (7.8 mg/kg/day, based on the average maternal weight of 64 kg), and less than 1% of the pediatric dose (15 mg/kg/day) for children greater than 6 months of age.

A prospective observational study of 55 breastfed infants of mothers taking a macrolide antibacterial (6 were exposed to clarithromycin) were compared to 36 breastfed infants of mothers taking amoxicillin. Adverse reactions were comparable in both groups. Adverse reactions occurred in 12.7% of infants exposed to macrolides and included rash, diarrhea, loss of appetite, and somnolence.

Animal Data

No studies with the combination of vonoprazan and amoxicillin and/or clarithromycin were conducted to examine the effect of lactational exposure on animal offspring.

Vonoprazan:

In a PPND study in rats, in which the dams were administered oral vonoprazan during gestation and through lactation at up to 22-times the MRHD (based on a comparison of AUC), liver discoloration occurred in offspring from the high dose group *[see Use in Specific Populations (8.1)]*.

Liver discoloration associated with necrosis, fibrosis and hemorrhage in the offspring of dosed rats was also seen in dose-range finding studies and limited, non-standard, follow-up, mechanistic studies, including offspring in lactation only studies. These effects were reported in pups on LD 4 at doses from 3 to 100 mg/kg/day (approximately 0.2 - to 22-fold the MRHD based on an AUC values extrapolated from the PPND study) and on LD 14 at doses from 10 to 100 mg/kg/day (approximately 1- to 22-fold the MRHD based on an extrapolated AUC comparisons). In mechanistic studies, liver effects were observed in offspring treated only during lactation but not in offspring from animals only treated during gestation. In some of these studies, this finding was associated with increased offspring stomach weights that was reversed along with liver discoloration by concomitant treatment with a gastrointestinal prokinetic agent.

8.3. Females and Males of Reproductive Potential

Infertility

Males

Clarithromycin:

Based on animal fertility study findings for clarithromycin, VOQUEZNA TRIPLE PAK may impair fertility in males of reproductive potential *[see Nonclinical Toxicology (13.1)].*

8.4. Pediatric Use

Safety and effectiveness of VOQUEZNA TRIPLE PAK or VOQUEZNA DUAL PAK in pediatric patients have not been established.

8.5. Geriatric Use

Geriatric Use for the Individual Components of VOQUEZNA TRIPLE PAK and VOQUEZNA DUAL PAK

Amoxicillin and Clarithromycin

Amoxicillin and clarithromycin are known to be substantially excreted by the kidney, and the risk of adverse reactions to these drugs may be greater in patients with impaired renal function and it may be useful to monitor renal function *[see Use in Specific Populations (8.6)].*

Vonoprazan

There were 218 patients aged 65 years and older in the clinical study of VOQUEZNA TRIPLE PAK and VOQUEZNA DUAL PAK for the treatment of *H. pylori* infection *[see Clinical Studies (14)].* Of the total number of vonoprazan-treated subjects (N=694), there were 153 (22.0%) patients aged 65 years and older and 18 (2.6%) patients were aged 75 years and older. No overall differences in safety or effectiveness were observed between these patients and younger adult patients.

Amoxicillin

An analysis of clinical studies of amoxicillin was conducted to determine whether subjects aged 65 and over respond differently from younger subjects. These analyses have not identified differences in responses between the elderly and younger patients, but a greater sensitivity of some older individuals cannot be ruled out.

This drug is known to be substantially excreted by the kidney, and the risk of toxic reactions to this drug may be greater in patients with impaired renal function. Because elderly patients are more likely to have decreased renal function, and it may be useful to monitor renal function.

Clarithromycin

In a steady-state study in which healthy elderly subjects (65 years to 81 years of age) were given 500 mg of clarithromycin every 12 hours, the maximum serum concentrations and area under the curves of clarithromycin and 14-OH clarithromycin were increased compared to those achieved in healthy young adults. These changes in pharmacokinetics parallel known age-related decreases in renal function. In clinical trials, elderly patients did not have an increased incidence of adverse reactions when compared to younger patients. Elderly patients may be more susceptible to development of *torsades de pointes* arrhythmias than younger patients *[see Warnings and Precautions (5.2)].*

Most reports of acute kidney injury with calcium channel blockers metabolized by CYP3A4 (e.g., verapamil, amlodipine, diltiazem, nifedipine) involved elderly patients 65 years of age or older *[see Warnings and Precautions (5.2)].*

Especially in elderly patients, there have been reports of colchicine toxicity with concomitant use of clarithromycin and colchicine, some of which occurred in patients with renal insufficiency. Deaths have been reported in some patients *[see Contraindications (4.2) and Warnings and Precautions (5.2)].*

8.6. Renal Impairment

No dosage adjustment of VOQUEZNA TRIPLE PAK or VOQUEZNA DUAL PAK is recommended in patients with mild to moderate renal impairment (eGFR 30 to 89 mL/min). Avoid the use of VOQUEZNA TRIPLE PAK or VOQUEZNA DUAL PAK in patients with severe renal impairment (eGFR < 30 mL/min) *[see Clinical Pharmacology (12.3)].*

8.7. Hepatic Impairment

No dosage adjustment of VOQUEZNA TRIPLE PAK or VOQUEZNA DUAL PAK is recommended in patients with mild hepatic impairment (Child-Pugh A). Avoid the use of VOQUEZNA TRIPLE PAK or VOQUEZNA DUAL PAK in patients with moderate to severe hepatic impairment (Child-Pugh B or C) *[see Clinical Pharmacology (12.3)].*

10. OVERDOSAGE

No information is available on accidental overdosage of VOQUEZNA TRIPLE PAK or VOQUEZNA DUAL PAK in humans.

In case of an overdose, patients should contact a physician, poison control center, or emergency room. The available overdosage information for each of the individual components in VOQUEZNA TRIPLE PAK and VOQUEZNA DUAL PAK are summarized below:

<u>Vonoprazan</u>

There have been no reports of overdose with vonoprazan. In clinical studies, a single dose of 120 mg resulted in no serious adverse reactions. Vonoprazan is not removed from the circulation by hemodialysis. If overdose occurs, treatment should be symptomatic and supportive.

<u>Amoxicillin</u>

In case of amoxicillin overdosage, discontinue medication, treat symptomatically and institute supportive measures as needed. A prospective study of 51 pediatric patients at a poison-control center suggested that overdosages of less than 250 mg/kg of amoxicillin are not associated with significant clinical symptoms.

Interstitial nephritis resulting in oliguric renal failure has been reported in a small number of patients after overdosage with amoxicillin.

Crystalluria, in some cases leading to renal failure, has also been reported after amoxicillin overdosage in adult and pediatric patients. In case of overdosage, adequate fluid intake and diuresis should be maintained to reduce the risk of amoxicillin crystalluria.

Renal impairment appears to be reversible with cessation of drug administration. High blood levels may occur more readily in patients with impaired renal function because of decreased renal clearance of amoxicillin. Amoxicillin can be removed from circulation by hemodialysis.

<u>Clarithromycin</u>

Overdosage of clarithromycin can cause gastrointestinal symptoms such as abdominal pain, vomiting, nausea, and diarrhea.

Treat adverse reactions accompanying overdosage by the prompt elimination of unabsorbed drug and supportive measures. As with other macrolides, clarithromycin serum concentrations are not expected to be appreciably affected by hemodialysis or peritoneal dialysis.

11. DESCRIPTION

VOQUEZNA TRIPLE PAK contains vonoprazan tablets, 20 mg, amoxicillin capsules, 500 mg and clarithromycin tablets, 500 mg for oral administration. VOQUEZNA DUAL PAK contains vonoprazan tablets, 20 mg and amoxicillin capsules, 500 mg for oral administration.

Vonoprazan Tablets

Vonoprazan (as the fumarate), is a potassium-competitive acid blocker (PCAB). Chemically, it is 1*H*-Pyrrole-3-methanamine, 5-(2-fluorophenyl)-*N*-methyl-1-(3-pyridinylsulfonyl)-,2-butenedioate (1:1). Its empirical formula is $C_{17}H_{16}FN_3O_2S \bullet C_4H_4O_4$ with a molecular weight of 461.5. Vonoprazan has the following structure:

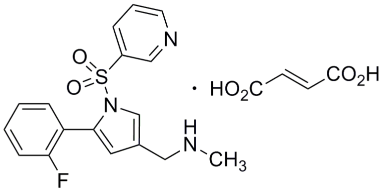

Vonoprazan fumarate is white to nearly white crystals or crystalline powder which melts at 194.8°C . Vonoprazan fumarate is soluble in dimethyl sulfoxide; sparingly soluble in *N,N*–dimethylacetamide, slightly soluble in *N,N*-dimethylformamide, methanol and water; very slightly soluble in ethanol (99.5); and practically insoluble in 2-propanol, acetone, 1-octanol and acetonitrile.

Each film-coated tablet contains 20 mg of vonoprazan, present as 26.72 mg of vonoprazan fumarate and the following inactive ingredients: croscarmellose sodium, ferric oxide red, fumaric acid, hydroxypropyl cellulose, hypromellose, magnesium stearate, mannitol, microcrystalline cellulose, polyethylene glycol 8000, and titanium dioxide.

Amoxicillin Capsules

Amoxicillin is a penicillin class antibacterial, with a broad spectrum of bactericidal activity against many gram-positive and gram-negative microorganisms. Chemically it is (2*S*, 5*R*, 6*R*)-6-[(*R*)-(-)-2-amino-2-(*p*-hydroxyphenyl) acetamido]-3,3-dimethyl-7-oxo-4-thia-1-azabicyclo [3.2.0] heptane-2-carboxylic acid trihydrate. The molecular formula is $C_{16}H_{19}N_3O_5S \bullet 3H_2O$ and the molecular weight is 419.45. Amoxicillin has the following structure:

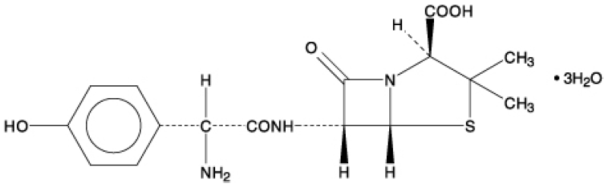

Each amoxicillin capsule, with yellow opaque cap and body, contains 500 mg amoxicillin as the trihydrate. Inactive ingredients: Capsule shells – ammonium hydroxide, black ferric oxide, gelatin, potassium hydroxide, propylene glycol, shellac, titanium dioxide and yellow ferric oxide; Capsule contents – cellulose microcrystalline and magnesium stearate.

Meets USP Dissolution Test 2.

Clarithromycin Tablets

Clarithromycin is a semi-synthetic macrolide antimicrobial for oral use. Chemically, it is 6-*O*-methylerythromycin. The molecular formula is $C_{38}H_{69}NO_{13}$, and the molecular weight is 747.96. Clarithromycin has the following structure:

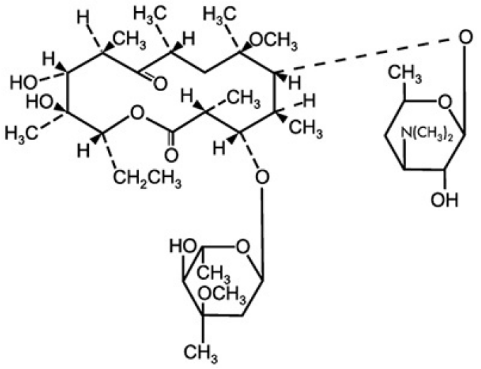

Clarithromycin is a white to off-white crystalline powder. It is soluble in acetone, slightly soluble in methanol, ethanol, and acetonitrile, and practically insoluble in water.

Each clarithromycin tablet contains 500 mg of clarithromycin and the following inactive ingredients: croscarmellose sodium, hypromellose, magnesium stearate, microcrystalline cellulose, polyethylene glycol, polysorbate 80, povidone, talc and titanium dioxide.

12. CLINICAL PHARMACOLOGY

12.1. Mechanism of Action

Vonoprazan suppresses basal and stimulated gastric acid secretion at the secretory surface of the gastric parietal cell through inhibition of the H^+, K^+-ATPase enzyme system in a potassium competitive manner. Because this enzyme is regarded as the acid (proton) pump within the parietal cell, vonoprazan has been characterized as a type of gastric proton-pump inhibitor, in that it blocks the final step of acid production. Vonoprazan does not require activation by acid. Vonoprazan may selectively concentrate in the parietal cells in both the resting and stimulated states. Vonoprazan binds to the active proton pumps in a noncovalent and reversible manner. Amoxicillin is an antibacterial drug. Clarithromycin is a macrolide antimicrobial drug *[see Microbiology (12.4)].*

Acid suppression enhances the replication of *H. pylori* bacteria and the stability and effectiveness of antimicrobials in the treatment of *H. pylori* infection.

12.2. Pharmacodynamics

<u>Vonoprazan</u>

Antisecretory Activity

Following a single 20 mg dose of vonoprazan, the onset of the antisecretory effect as measured by intragastric pH occurs within 2 to 3 hours. The elevated intragastric pH compared to placebo is maintained for over 24-hours after dosing. The inhibitory effect of vonoprazan on acid secretion increases with repeated daily dosing and antisecretory effect reached steady state by Day 4 with a mean (SD) 24-hour intragastric pH of 6.0 (1.5) following 20 mg once daily dose (not an approved recommended dosage). The antisecretory effect of vonoprazan decreases following drug discontinuation although intragastric pH remained elevated compared to placebo for 24 to 48 hours following the dose on Day 7.

Cardiac Electrophysiology

At a dose 6 times the maximum recommended dose, vonoprazan does not prolong the QT interval to any clinically relevant extent.

12.3. Pharmacokinetics

Pharmacokinetic (PK) parameters for vonoprazan 20 mg after a single dose (not an approved recommended dosage) and at steady state following twice daily administration are summarized in Table 6.

Table 6: Mean (%CV) Pharmacokinetic Parameters for Vonoprazan Following a Single Dose or at Steady State Following Twice Daily Dosing

PK Parameter	Single Dose (N=10)	Steady State (N=32)
T_{max} (h), median (range)	2.5 (1.0-4.0)	3.0 (1.0-6.0)
C_{max} (ng/mL)	25.2 (39.7)	37.8 (36.1)
AUC_{0-12h} (ng*hr/mL)	154.8 (25.2)	272.5 (30.5)
$t_{1/2}$ (h)	7.1 (10.1)	6.8 (22.7)
CL/F (L/h)	97.3 (36.3)	81.3 (35.7)
V_z/F (L)	1001 (39.6)	782.7 (34.4)

C_{max} = Maximum plasma concentration; AUC_{0-12h} =Area under the plasma concentration-time curve from time 0 to the end of the 12-hour dosing interval; T_{max} = Time to reach C_{max}; $t_{1/2}$ = Elimination half-life, CL/F = Apparent oral clearance, V_z/F = Apparent oral volume of distribution.

<u>Vonoprazan</u>

Absorption

Vonoprazan exhibits time independent pharmacokinetics and steady state concentrations are achieved by Day 3 to 4. After multiple doses of vonoprazan ranging from 10 mg (0.5 times the lowest approved recommended single dosage) to 40 mg (2 times the highest approved recommended single dosage) once daily for 7 days in healthy subjects, C_{max} and AUC values for vonoprazan increased in an approximately dose-proportional manner.

Steady state mean plasma exposure of vonoprazan following 20 mg twice daily dosing (AUC_{0-12h} = 273 hr*ng/mL, N=10) was approximately 1.8-fold higher compared to Day 1 (AUC_{0-12h} = 155 hr*ng/mL, N=10).

Effect of Food:
In a food effect study in healthy subjects (N=24) receiving vonoprazan 20 mg, a high-fat meal resulted in a 5% increase in C_{max}, a 15% increase in AUC, and a delay in median T_{max} of 2 hours. These changes are not considered to be clinically significant.

Distribution
Plasma protein binding of vonoprazan ranged from 85 to 88% in healthy subjects and was independent of concentration from 0.1 to 10 mcg/mL.

Elimination

Metabolism:

Vonoprazan is metabolized to inactive metabolites via multiple pathways by a combination of cytochrome P450 (CYP) isoforms (CYP3A4/5, CYP2B6, CYP2C19, CYP2C9 and CYP2D6) along with sulfo- and glucuronosyl-transferases. CYP2C19 polymorphisms have been evaluated in clinical studies and there were no considerable differences in the pharmacokinetics of vonoprazan based on CYP2C19 metabolizer status.

Excretion:

Following oral administration of radiolabeled vonoprazan, approximately 67% of the radiolabeled dose (8% as unchanged vonoprazan) was recovered in urine and 31% (1.4% as unchanged vonoprazan) was recovered in feces.

Specific Populations

Sex, Race or Ethnicity:

There were no clinically significant differences in the pharmacokinetics of vonoprazan based on sex or race/ethnicity.

Patients with Renal Impairment

The pharmacokinetics of vonoprazan administered as a single 20 mg dose in patients with mild (N=8), moderate (N=8) or severe (N=8) renal impairment were compared to those with normal renal function (N=13). Compared to subjects with normal renal function, systemic exposure (AUC_{∞}) was 1.7-, 1.3- and 2.4- times greater in patients with mild, moderate, and severe renal impairment, respectively. In subjects requiring dialysis (N=8), AUC_{∞} estimates were 1.3-fold greater compared to estimates from subjects with normal renal function. Protein binding of vonoprazan is not affected by impaired renal function. In patients requiring dialysis, vonoprazan was present in the dialysate and represented 0.94% of the dose administered.

Patients with Hepatic Impairment

The pharmacokinetics of vonoprazan administered as a single 20 mg dose in patients with mild [Child-Pugh Class A (N=8)], moderate [Child-Pugh Class B (N=8)] or severe [Child-Pugh Class C (N=6)] hepatic impairment were compared to those with normal hepatic function (N=12). Compared to subjects with normal hepatic function, systemic exposure (AUC_{∞}) of vonoprazan was 1.2-, 2.4- and 2.6-times greater in patients with mild, moderate, and severe hepatic impairment, respectively. Protein binding of vonoprazan is not affected by impaired hepatic function.

Drug Interaction Studies

In vitro studies:

<u>Cytochrome P450 (CYP450) Enzymes:</u>

In vitro studies have shown that vonoprazan directly and time-dependently inhibits CYP2B6, CYP2C19, and CYP3A4/5.

<u>Transporter Systems:</u>

Vonoprazan inhibits multidrug and toxin extrusion protein 1 (MATE1) and organic cation transporter 1 (OCT1), but only at concentrations higher than clinically relevant.

Clinical Studies:

<u>Combination Therapy with Vonoprazan, Amoxicillin and Clarithromycin:</u>

When vonoprazan 20 mg, amoxicillin 750 mg, and clarithromycin 400 mg were co-administered twice daily for 7 days (N=11), there was no effect on pharmacokinetics of amoxicillin compared to administration of amoxicillin alone. However, vonoprazan C_{max} and AUC_{0-12h} increased by 87% and 85%, respectively, and clarithromycin, C_{max} and AUC_{0-12h} increased by 64% and 45%, respectively, compared to administration of each component alone.

Effect of Vonoprazan on CYP3A4 Substrates:

When a single oral dose of midazolam 2 mg was administered following vonoprazan 20 mg twice daily for 7 days (N=20), midazolam AUC_{∞} increased 93% compared to administration of midazolam alone.

Effect of CYP3A Inhibitors on Vonoprazan:

When a single 40 mg (2 times the highest approved recommended single dosage) dose of vonoprazan was administered with clarithromycin 500 mg twice daily for 7 days (N=16), vonoprazan AUC_{∞} increased 58% compared to administration of vonoprazan alone.

Model-Informed Approaches:

Effect of CYP3A Inducers on Vonoprazan:

Vonoprazan exposures are predicted to be 80% lower when co-administered with a strong CYP3A4 inducer such as rifampicin and 50% lower when co-administered with a moderate CYP3A4 inducer such as efavirenz.

Amoxicillin

Absorption

Amoxicillin is stable in the presence of gastric acid and is rapidly absorbed after oral administration. Orally administered doses of 500-mg amoxicillin capsules result in average peak blood levels 1 to 2 hours after administration in the range of 5.5 mcg/mL to 7.5 mcg/mL, respectively.

Distribution

Amoxicillin diffuses readily into most body tissues and fluids, with the exception of brain and spinal fluid, except when meninges are inflamed. In blood serum, amoxicillin is approximately 20% protein-bound. Following a 1-gram dose and utilizing a special skin window technique to determine levels of the antibacterial, it was noted that therapeutic levels were found in the interstitial fluid.

Metabolism and Excretion

The half-life of amoxicillin is 61.3 minutes. Approximately 60% of an orally administered dose of amoxicillin is excreted in the urine within 6 to 8 hours. Detectable serum levels are observed up to 8 hours after an orally administered dose of amoxicillin. Since most of the amoxicillin is excreted unchanged in the urine, its excretion can be delayed by concurrent administration of probenecid.

Clarithromycin

Absorption

For a single 500 mg dose of clarithromycin, food slightly delays the onset of clarithromycin absorption, increasing the peak time from approximately 2 to 2.5 hours. Food also increases the clarithromycin peak plasma concentration by about 24%, but does not affect the extent of clarithromycin bioavailability. Food does not affect the onset of formation of the active metabolite, 14-OH clarithromycin or its peak plasma concentration but does slightly decrease the extent of metabolite formation, indicated by an 11% decrease in AUC. Therefore, clarithromycin may be given

without regard to food. In non-fasting healthy human subjects (males and females), peak plasma concentrations were attained within 2 to 3 hours after oral dosing.

Distribution

Clarithromycin and the 14-OH clarithromycin metabolite distribute readily into body tissues and fluids. There are no data available on cerebrospinal fluid penetration. Because of high intracellular concentrations, tissue concentrations are higher than serum concentrations.

Metabolism and Elimination

Steady-state peak plasma clarithromycin concentrations were attained within 3 days and were 3 mcg/mL to 4 mcg/mL with a 500 mg dose administered every 8 hours to 12 hours. The elimination half-life of clarithromycin was 5 hours to 7 hours with 500 mg administered every 8 hours to 12 hours. The nonlinearity of clarithromycin pharmacokinetics is slight at the recommended doses of 500 mg administered every 8 hours to 12 hours. With a 500 mg every 8 hours to 12 hours dosing, the peak steady-state concentration of 14-OH clarithromycin is slightly higher (up to 1 mcg/mL), and its elimination half-life is about 7 hours to 9 hours. With any of these dosing regimens, the steady-state concentration of this metabolite is generally attained within 3 days to 4 days.

After a 500 mg tablet every 12 hours, the urinary excretion of clarithromycin is approximately 30%. The renal clearance of clarithromycin is, however, relatively independent of the dose size and approximates the normal glomerular filtration rate. The major metabolite found in urine is 14-OH clarithromycin, which accounts for an additional 10% to 15% of the dose with a 500 mg tablet administered every 12 hours.

Patients with Hepatic Impairment

The steady-state concentrations of clarithromycin in subjects with impaired hepatic function did not differ from those in normal subjects; however, the 14-OH clarithromycin concentrations were lower in the hepatically impaired subjects. The decreased formation of 14-OH clarithromycin was at least partially offset by an increase in renal clearance of clarithromycin in the subjects with impaired hepatic function when compared to healthy subjects.

Patients with Renal Impairment

The pharmacokinetics of clarithromycin were also altered in subjects with impaired renal function.

Drug Interaction Studies

Fluconazole:

Following administration of fluconazole 200 mg daily and clarithromycin 500 mg twice daily to 21 healthy volunteers, the steady-state clarithromycin C_{min} and AUC increased 33% and 18%, respectively. Clarithromycin exposures were increased and steady-state concentrations of 14-OH clarithromycin were not significantly affected by concomitant administration of fluconazole.

Colchicine:

When a single dose of colchicine 0.6 mg was administered with clarithromycin 250 mg twice daily for 7 days, the colchicine C_{max} increased 197% and the $AUC_{0-\infty}$ increased 239% compared to administration of colchicine alone.

Atazanavir:

Following administration of clarithromycin (500 mg twice daily) with atazanavir (400 mg once daily), the clarithromycin AUC increased 94%, the 14-OH clarithromycin AUC decreased 70% and the atazanavir AUC increased 28%.

Ritonavir:

Concomitant administration of clarithromycin and ritonavir (N=22) resulted in a 77% increase in clarithromycin AUC and a 100% decrease in the AUC of 14-OH clarithromycin.

Saquinavir:

Following administration of clarithromycin (500 mg twice daily) and saquinavir (soft gelatin capsules, 1200 mg tid) to 12 healthy volunteers, the steady-state saquinavir AUC and C_{max} increased 177% and 187% respectively compared to administration of saquinavir alone. Clarithromycin AUC and C_{max} increased 45% and 39% respectively, whereas the 14-OH clarithromycin AUC and C_{max} decreased 24% and 34% respectively, compared to administration with clarithromycin alone.

Didanosine:

Simultaneous administration of clarithromycin tablets and didanosine to 12 HIV-infected adult patients resulted in no statistically significant change in didanosine pharmacokinetics.

Zidovudine:

Following administration of clarithromycin 500 mg tablets twice daily with zidovudine 100 mg every 4 hours, the steady-state zidovudine AUC decreased 12% compared to administration of zidovudine alone (N=4). Individual values ranged from a decrease of 34% to an increase of 14%. When clarithromycin tablets were administered two to four hours prior to zidovudine, the steady-state zidovudine C_{max} increased 100% whereas the AUC was unaffected (N=24).

Omeprazole:

Clarithromycin 500 mg every 8 hours was given in combination with omeprazole 40 mg daily to healthy adult subjects. The steady-state plasma concentrations of omeprazole were increased (C_{max}, AUC_{0-24}, and $t_{½}$ increases of 30%, 89%, and 34%, respectively), by the concomitant administration of clarithromycin.

The plasma levels of clarithromycin and 14-OH clarithromycin were increased by the concomitant administration of omeprazole. For clarithromycin, the mean C_{max} was 10% greater, the mean C_{min} was 27% greater, and the mean AUC_{0-8} was 15% greater when clarithromycin was administered with omeprazole than when clarithromycin was administered alone. Similar results were seen for 14-OH clarithromycin, the mean C_{max} was 45% greater, the mean C_{min} was 57% greater, and the mean AUC_{0-8} was 45% greater. Clarithromycin concentrations in the gastric tissue and mucus were also increased by concomitant administration of omeprazole.

Table 7: Clarithromycin Tissue Concentrations 2 hours after Dose (mcg/mL)/(mcg/g)

Treatment	N	Antrum	Fundus	N	Mucus
Clarithromycin	5	10.48 ± 2.01	20.81 ± 7.64	4	4.15 ± 7.74
Clarithromycin + Omeprazole	5	19.96 ± 4.71	24.25 ± 6.37	4	39.29 ± 32.79

Theophylline:

In two studies in which theophylline was administered with clarithromycin (a theophylline sustained-release formulation was dosed at either 6.5 mg/kg or 12 mg/kg together with 250 or 500 mg q12h

clarithromycin), the steady-state levels of C_{max}, C_{min}, and the AUC of theophylline increased about 20%.

Midazolam:

When a single dose of midazolam was co-administered with clarithromycin tablets (500 mg twice daily for 7 days), midazolam AUC increased 174% after intravenous administration of midazolam and 600% after oral administration.

12.4. Microbiology

Mechanism of Action

Amoxicillin is similar to penicillin in its bactericidal action against susceptible bacteria during the stage of active multiplication. It acts through the inhibition of cell wall biosynthesis that leads to the death of the bacteria.

Clarithromycin exerts its antibacterial activity by binding to the 50S ribosomal subunit of susceptible bacteria resulting in inhibition of protein synthesis.

Resistance

Resistance to amoxicillin is mediated primarily through enzymes called beta-lactamases that cleave the beta-lactam ring of amoxicillin, rendering it inactive.

The major routes of clarithromycin resistance are modification of the 23S rRNA in the 50S ribosomal subunit to insensitivity or drug efflux pumps. Beta-lactamase production should have no effect on clarithromycin activity.

If *H. pylori* is not eradicated after treatment with clarithromycin-containing combination regimens, patients may develop clarithromycin resistance in *H. pylori* isolates. Therefore, for patients who fail therapy, clarithromycin susceptibility testing should be done, if possible.

Antimicrobial Activity

Culture and sensitivity testing of bacteria are not routinely performed to establish the diagnosis of *H. pylori* infection *[see Clinical Studies (14)]*. The following in vitro data are available, but their clinical significance is unknown. Clarithromycin and amoxicillin are active in vitro against most isolates of *H. pylori*.

Susceptibility Testing

For specific information regarding susceptibility test interpretive criteria and associated test methods and quality control standards recognized by FDA for this drug, please see: https://www.fda.gov/STIC.

Effects on Gastrointestinal Microbial Ecology

Decreased gastric acidity due to any means, increases gastric counts of bacteria normally present in the gastrointestinal tract. Vonoprazan decreases gastric acidity, VOQUEZNA TRIPLE PAK or VOQUEZNA DUAL PAK may lead to a slightly increased risk of gastrointestinal infections due to pathogens such as *Salmonella* and *Campylobacter* and, in hospitalized patients, possibly also due to *C. difficile*.

13. NONCLINICAL TOXICOLOGY

13.1. Carcinogenesis, Mutagenesis, Impairment of Fertility

No adequate or well-controlled long-term studies have been performed to evaluate the effect of VOQUEZNA TRIPLE PAK and VOQUEZNA DUAL PAK on carcinogenesis, mutagenesis, or impairment of fertility.

Vonoprazan

Carcinogenicity

In a 24-month carcinogenicity study in mice, vonoprazan at daily oral doses of 6, 20, 60 and 200 mg/kg/day (approximately 0.4, 4, 19, and 93 times the MRHD based on AUC) produced hyperplasia of neuroendocrine cells, gastropathy and benign and/or malignant neuroendocrine cell tumors (carcinoids) in the stomach at all doses in males and at 60 mg/kg/day and greater in females. In liver, increased incidences of hepatocellular adenoma and carcinomas were observed at doses of 20 mg/kg/day and greater in males and 60 mg/kg/day and greater in females.

In a 24-month carcinogenicity study in Sprague-Dawley rats, vonoprazan at daily oral doses of 5, 15, 50 and 150 mg/kg/day (approximately 0.6, 4, 19, and 65 times the MRHD based on AUC) produced benign and/or malignant neuroendocrine cell tumors in the stomach in both male and female rats at doses of 5 mg/kg/day or more. Increased incidence of hepatocellular adenoma and carcinomas and hepatocholangiocellular adenomas and carcinomas were observed at doses of 50 and 150 mg/kg/day.

In both mice and rats, neuroendocrine tumors in the stomach occurred in association with neuroendocrine hyperplasia and gastropathy in the stomach and increased plasma gastrin concentrations that are consistent with inhibition of gastric acid secretion. Carcinoid tumors have also been observed in rats subjected to fundectomy or long-term treatment with proton pump inhibitors or high doses of H_2-receptor antagonists.

Mutagenesis

Vonoprazan was negative for mutagenicity in the *in vitro* Ames test, in an *in vitro* clastogenicity assay in Chinese Hamster cells and *in vivo* in a rat bone marrow micronucleus study.

Impairment of Fertility

Vonoprazan at oral doses up to 300 mg/kg/day in rats (approximately 133 times the MRHD based on AUC from a separate study in nonpregnant animals administered the same dose) was found to have no effect on fertility and reproductive performance. Elongation of the estrous cycle was observed in rat at doses equivalent to 133 times the MRHD based on AUC.

Amoxicillin

Carcinogenicity

Long-term studies in animals have not been performed to evaluate carcinogenic potential.

Mutagenesis

Studies to detect mutagenic potential of amoxicillin alone have not been conducted; however, the following information is available from tests on a 4:1 mixture of amoxicillin and potassium clavulanate. Amoxicillin and potassium clavulanate were non-mutagenic in the Ames bacterial mutation assay, and the yeast gene conversion assay. Amoxicillin and potassium clavulanate were weakly positive in the mouse lymphoma assay, but the trend toward increased mutation frequencies in this assay

occurred at doses that were also associated with decreased cell survival. Amoxicillin and potassium clavulanate were negative in the mouse micronucleus test and in the dominant lethal assay in mice. Potassium clavulanate alone was tested in the Ames bacterial mutation assay and in the mouse micronucleus test and was negative in each of these assays.

Impairment of Fertility

In a multi-generation reproduction study in rats, no impairment of fertility or other adverse reproductive effects were seen at doses up to 500 mg/kg (approximately 1.6 times the human dose in mg/m^2).

<u>Clarithromycin</u>

Mutagenesis

The following *in vitro* mutagenicity tests have been conducted with clarithromycin:

- *Salmonella*/Mammalian Microsomes Test
- Bacterial Induced Mutation Frequency Test
- *In Vitro* Chromosome Aberration Test
- Rat Hepatocyte DNA Synthesis Assay
- Mouse Lymphoma Assay
- Mouse Dominant Lethal Study
- Mouse Micronucleus Test

All tests had negative results except the *in vitro* chromosome aberration test which was positive in one test and negative in another. In addition, a bacterial reverse-mutation test (Ames test) has been performed on clarithromycin metabolites with negative results.

Impairment of Fertility
Fertility and reproduction studies have shown that daily doses of up to 160 mg/kg/day to male and female rats caused no adverse effects on the estrous cycle, fertility, parturition, or number and viability of offspring. Plasma levels in rats after 150 mg/kg/day were twice the human serum levels.

Testicular atrophy occurred in rats at doses 7 times, in dogs at doses 3 times, and in monkeys at doses 8 times greater than the maximum human daily dose (on a BSA comparisons).

13.2. Animal Toxicology and/or Pharmacology

<u>Clarithromycin</u>

Corneal opacity occurred in dogs at doses 12 times and in monkeys at doses 8 times greater than the maximum human daily dose (on BSA comparisons). Lymphoid depletion occurred in dogs at doses 3 times greater than and in monkeys at doses 2 times greater than the maximum human daily dose (on BSA comparisons).

14. CLINICAL STUDIES

The effectiveness and safety of VOQUEZNA TRIPLE PAK and VOQUEZNA DUAL PAK were evaluated in a randomized, controlled, double-blind triple therapy/open-label dual therapy study conducted in the United States and Europe in treatment-naïve *H. pylori*-positive adult patients with at least one clinical condition: dyspepsia lasting at least 2 weeks, functional dyspepsia, recent/new diagnosis of peptic ulcer, peptic ulcer not treated for *H. pylori* infection, or a stable dose of long-term

NSAID treatment (NCT 04167670). Patients were randomized 1:1:1 to vonoprazan 20 mg twice daily plus amoxicillin 1,000 mg twice daily plus clarithromycin 500 mg twice daily (VOQUEZNA TRIPLE PAK) or vonoprazan 20 mg twice daily plus amoxicillin 1,000 mg three times daily (VOQUEZNA DUAL PAK) or lansoprazole 30 mg twice daily plus amoxicillin 1,000 mg twice daily plus clarithromycin 500 mg twice daily (LAC) administered for 14 consecutive days.

H. pylori infection at baseline was defined as positive by ^{13}C urea breath test (UBT) and follow-up upper endoscopy (culture or histology). *H. pylori* eradication was confirmed with a negative ^{13}C UBT test-of-cure at ≥ 27 days post-therapy. Patients with negative test results were considered treatment successes. Patients who tested positive for *H. pylori* infection and patients with missing results from the test-of-cure visit were considered treatment failures.

VOQUEZNA TRIPLE PAK and VOQUEZNA DUAL PAK were shown to be noninferior to LAC in patients who did not have a clarithromycin or amoxicillin resistant strain of *H. pylori* at baseline. VOQUEZNA TRIPLE PAK and VOQUEZNA DUAL PAK were shown to be superior to LAC in patients who had a clarithromycin resistant strain of *H. pylori* at baseline and in the overall population.

H. pylori eradication rates are shown in Table 8 for VOQUEZNA TRIPLE PAK and VOQUEZNA DUAL PAK compared to LAC.

Table 8: Eradication Rates of H. pylori in Patients Receiving VOQUEZNA TRIPLE PAK, VOQUEZNA DUAL PAK, or LAC at ≥27 Days Post-therapy - mITT

	**VOQUEZNA TRIPLE PAK<br>%<br>(n)**	**VOQUEZNA DUAL PAK<br>%<br>(n)**	**LAC<br>%<br>(n)**
**Patients with *H. pylori* infection who did not have a clarithromycin or amoxicillin resistant strain at baseline[a]**	84.7<br>(222)	78.5<br>(208)	78.8<br>(201)
Treatment Difference from LAC<br>(95% CI)	5.9[b]<br>(-0.8, 12.6)	-0.3[c]<br>(-7.4, 6.8)	
**All randomized patients with *H. pylori* infection at baseline**	80.8<br>(273)	77.2<br>(250)	68.5<br>(226)
Treatment Difference from LAC<br>(95% CI)	12.3[d]<br>(5.7, 18.8)	8.7[e]<br>(1.9, 15.4)	
**Patients with *H. pylori* infection who had a clarithromycin resistant strain of *H. pylori* at baseline**	65.8<br>(48)	69.6<br>(39)	31.9<br>(23)
Treatment Difference from LAC<br>(95% CI)	33.8[f]<br>(17.7, 48.1)	37.7[f]<br>(20.5, 52.6)	

LAC = lansoprazole, amoxicillin, clarithromycin triple therapy regimen; CI = confidence interval calculated via the Miettinen and Nurminen method

Modified intent to treat (mITT) population: Patients were included in the MITT analysis if they had documented *H. pylori* infection at baseline.

[a] Clarithromycin resistant strains of *H. pylori* were considered those with an MIC ≥ 1 µg/mL; amoxicillin resistant strains were considered those with an MIC > 0.125 µg/mL.

[b] p<0.0001 for test of non-inferiority versus LAC.

[c] p<0.01 for test of non-inferiority versus LAC.

[d] p=0.0003 for test of superiority versus LAC

[e] p=0.01 for test of superiority versus LAC.

[f] p<0.0001 for test of superiority versus LAC.

16. HOW SUPPLIED/STORAGE AND HANDLING

VOQUEZNA TRIPLE PAK is a co-package containing:

- Vonoprazan Tablets, 20 mg: pale red, oval, film-coated tablets debossed V20 on one side and plain on the other side.
- Amoxicillin Capsules, 500 mg: yellow, opaque, hard gelatin capsules imprinted with AMOX 500 on one side and GG 849 on the other side.
- Clarithromycin Tablets, 500 mg: white, oval, film-coated tablets debossed GG C9 on one side and plain on the other side.

Vonoprazan tablets, amoxicillin capsules and clarithromycin tablets are supplied in separate blister cavities within the same blister card.

Each unit of use carton (NDC 81520-255-14) contains 56 tablets and 56 capsules divided into 14 daily dose blister cards.

Each daily blister card contains two vonoprazan tablets (20 mg each), four amoxicillin capsules (500 mg each) and two clarithromycin tablets (500 mg each), and indicates which tablets and capsules need to be taken in the morning and evening.

Store between 20°C and 25°C (68°F and 77°F). Brief exposure to 15°C to 30°C (59°F to 86°F) permitted (see USP Controlled Room Temperature). Protect from light.

VOQUEZNA DUAL PAK is a co-package containing:

- Vonoprazan Tablets, 20 mg: pale red, oval, film-coated tablets debossed V20 on one side and plain on the reverse side.
- Amoxicillin Capsules, 500 mg: yellow, opaque, hard gelatin capsules imprinted with AMOX 500 on one side and GG 849 on the other side.

Vonoprazan tablets and amoxicillin capsules are supplied in separate blister cavities within the same blister card.

Each unit of use carton (NDC 81520-250-14) contains 28 tablets and 84 capsules divided into 14 daily dose blister cards.

Each daily blister card contains two vonoprazan tablets (20 mg each) and six amoxicillin capsules (500 mg each) and indicates which tablets and capsules need to be taken in the morning, mid-day and evening.

Store between 20°C and 25°C (68°F and 77°F). Brief exposure to 15°C to 30°C (59°F to 86°F) permitted (see USP Controlled Room Temperature).

17. PATIENT COUNSELING INFORMATION

Hypersensitivity Reactions

Patients should be aware that VOQUEZNA TRIPLE PAK and VOQUEZNA DUAL PAK can cause allergic reactions in some individuals. Advise the patient to call their healthcare provider immediately if they develop a new rash, urticaria, drug eruptions, swelling of the face, difficulty in breathing or

other symptoms of allergic reactions *[see Warnings and Precautions (5.1) and Adverse Reactions (6.2)].*

Severe Cutaneous Adverse Reactions

Advise patients about the signs and symptoms of serious skin manifestations. Instruct patients to stop taking VOQUEZNA TRIPLE PAK or VOQUEZNA DUAL PAK immediately and promptly report the first signs or symptoms of skin rash, mucosal lesions, or any other sign of hypersensitivity *[see Warnings and Precautions (5.1) and Adverse Reactions (6.2)].*

Drug Interactions

Advise patients that VOQUEZNA TRIPLE PAK or the individual components of VOQUEZNA TRIPLE PAK may interact with some drugs; therefore, advise patients to report to their healthcare provider the use of any other medications including natural substitutes and nutritional supplements.

Diarrhea

Advise patients that diarrhea is a common problem caused by antibacterials including amoxicillin and clarithromycin, and it usually ends when the drugs are stopped. However, rarely after receiving treatment with VOQUEZNA TRIPLE PAK or VOQUEZNA DUAL PAK, patients can develop watery and bloody stools (with or without stomach cramps and fever) even as late as 2 or more months after having taken their last dose. If this occurs, instruct patients to contact their healthcare provider as soon as possible *[see Warnings and Precautions (5.1)].*

Embryo-Fetal Toxicity

Advise pregnant patients and females of reproductive potential that if pregnancy occurs while taking VOQUEZNA TRIPLE PAK, there is a potential risk to the fetus due to the clarithromycin component *[see Warnings and Precautions (5.2) and Use in Specific Populations (8.1)].*

Advise patients who are exposed to VOQUEZNA TRIPLE PAK or VOQUEZNA DUAL PAK during pregnancy to contact Phathom Pharmaceuticals, Inc. at 1-800-775-PHAT (7428).

Lactation

Advise the lactating women to pump and discard their milk during treatment with VOQUEZNA TRIPLE PAK or VOQUEZNA DUAL PAK and for 2 days after the therapy ends *[see Use in Specific Populations (8.2)].*

Infertility

Advise males of reproductive potential that VOQUEZNA TRIPLE PAK may impair fertility *[see Use in Specific Populations (8.3)].*

Potential for Dizziness, Vertigo and Confusion

There are no data on the effect of VOQUEZNA TRIPLE PAK on the ability to drive or use machines. However, counsel patients regarding the potential for dizziness, vertigo, confusion and disorientation, which may occur with clarithromycin, a component of VOQUEZNA TRIPLE PAK. The potential for these adverse reactions should be taken into account before patients drive or use machines.

Important Administration Instructions for VOQUEZNA TRIPLE PAK and VOQUEZNA DUAL PAK

- Take with or without food *[see Dosage and Administration (2)].*
- *Missed doses*: Advise patients that if a dose of VOQUEZNA TRIPLE PAK or VOQUEZNA DUAL PAK is missed, administer as soon as possible within 4 hours after the missed dose. If more than

4 hours have passed, skip the missed dose and administer the next dose on the regularly scheduled time. It is important for patients to complete the entire course of therapy *[see Dosage and Administration (2)]*.

- Counsel patients to continue the full course of VOQUEZNA TRIPLE PAK or VOQUEZNA DUAL PAK regardless of whether or not their symptoms improve. Counsel patients that treatment of *H. pylori* infection is important due to its association with stomach ulcers, atrophic gastritis and increased risk of gastric cancer.

Antibacterial Resistance

Patients should be counseled that antibacterial drugs including VOQUEZNA TRIPLE PAK or VOQUEZNA DUAL PAK should only be used to treat bacterial infections. They do not treat viral infections (e.g., the common cold). When VOQUEZNA TRIPLE PAK or VOQUEZNA DUAL PAK is prescribed to treat a bacterial infection, patients should be told that although it is common to feel better early in the course of therapy, the medication should be taken exactly as directed. Skipping doses or not completing the full course of therapy may (1) decrease the effectiveness of the immediate treatment and (2) increase the likelihood that bacteria will develop resistance and will not be treatable by VOQUEZNA TRIPLE PAK, VOQUEZNA DUAL PAK or other antibacterial drugs in the future *[see Warnings and Precautions (5.1)]*.

VOQUEZNA TRIPLE PAK and VOQUEZNA DUAL PAK are distributed by Phathom Pharmaceuticals, Inc., Buffalo Grove, IL, 60089, U.S.A.

Vonoprazan Tablets are manufactured for Phathom Pharmaceuticals, Inc., Buffalo Grove, IL 60089, U.S.A.

Amoxicillin Capsules and Clarithromycin Tablets are manufactured for Sandoz Inc., Princeton, NJ 08540, U.S.A.

VOQUEZNA, VOQUEZNA TRIPLE PAK, VOQUEZNA DUAL PAK and the VOQUEZNA logo are trademarks, and Phathom Pharmaceuticals is a registered trademark, of Phathom Pharmaceuticals, Inc.

© 2022 Phathom Pharmaceuticals, Inc. All rights reserved.

VOQ221 V1

46295608

NDC 81520-255-00
Rx only

VOQUEZNA TriplePak™
vonoprazan tablets, 20 mg
amoxicillin capsules, 500 mg
clarithromycin tablets, 500 mg

14 DAILY TREATMENT PACKS | TRIPLE THERAPY

PROFESSIONAL SAMPLE – NOT FOR RESALE

KEEP OUT OF REACH OF CHILDREN

VOQUEZNA TRIPLE PAK is distributed by Phathom Pharmaceuticals, Inc. Buffalo Grove, IL 60089, U.S.A.

VOQUEZNA, VOQUEZNA TRIPLE PAK, and the VOQUEZNA logo are trademarks of Phathom Pharmaceuticals, Inc.

NDC 81520-255-00
Rx only

VOQUEZNA TriplePak™
vonoprazan tablets, 20 mg
amoxicillin capsules, 500 mg
clarithromycin tablets, 500 mg

Co-packaged for oral use
Carton contains 56 tablets and 56 capsules

14 DAILY TREATMENT PACKS
TRIPLE THERAPY

PROFESSIONAL SAMPLE – NOT FOR RESALE

Phathom.
PHARMACEUTICALS

NDC 81520-255-00
Rx only

VOQUEZNA TriplePak™
vonoprazan tablets, 20 mg
amoxicillin capsules, 500 mg
clarithromycin tablets, 500 mg

14 DAILY TREATMENT PACKS | TRIPLE THERAPY

PROFESSIONAL SAMPLE – NOT FOR RESALE

Store between 20°C and 25°C (68°F and 77°F). Brief exposure to 15°C to 30°C (59°F to 86°F) permitted [see USP Controlled Room Temperature]. Protect from light.

See package insert for complete Prescribing Information.

NDC 81520-255-00
Rx only

EACH DAILY TREATMENT PACK CONTAINS:

2 vonoprazan tablets, 20 mg per tablet*
4 amoxicillin capsules, 500 mg per capsule**
2 clarithromycin tablets, 500 mg per tablet

*Each tablet contains 20 mg of vonoprazan (equivalent to 26.72 mg of vonoprazan fumarate).
**Each capsule contains amoxicillin trihydrate (equivalent to 500 mg amoxicillin).

PROFESSIONAL SAMPLE – NOT FOR RESALE

Vonoprazan tablets manufactured for Phathom Pharmaceuticals, Inc., Buffalo Grove, IL 60089, U.S.A. Product of Ireland.

Amoxicillin capsules, manufactured for Sandoz Inc., Princeton, NJ 08540, U.S.A. Product of Spain.

Clarithromycin tablets, manufactured for Sandoz Inc., Princeton, NJ 08540, U.S.A. Product of China.

Phathom.
PHARMACEUTICALS

0 381520 255003

EXP: YYYY MMM
LOT: 12345678AB

Phathom.
PHARMACEUTICALS

PROFESSIONAL SAMPLE – NOT FOR RESALE

14 DAILY TREATMENT PACKS
TRIPLE THERAPY

VOQUEZNA TriplePak™
vonoprazan tablets, 20 mg
amoxicillin capsules, 500 mg
clarithromycin tablets, 500 mg

NDC 81520-255-00
Rx only

46285516

NDC 81520-255-14
Rx only

VOQUEZNA *TriplePak*™
vonoprazan tablets, 20 mg
amoxicillin capsules, 500 mg
clarithromycin tablets, 500 mg

14 DAILY TREATMENT PACKS | TRIPLE THERAPY

KEEP OUT OF REACH OF CHILDREN

VOQUEZNA TRIPLE PAK is distributed by Phathom Pharmaceuticals, Inc. Buffalo Grove, IL 60089, U.S.A.

VOQUEZNA, VOQUEZNA TRIPLE PAK, and the VOQUEZNA logo are trademarks of Phathom Pharmaceuticals, Inc.

NDC 81520-255-14
Rx only

VOQUEZNA *TriplePak*™
vonoprazan tablets, 20 mg
amoxicillin capsules, 500 mg
clarithromycin tablets, 500 mg

Co-packaged for oral use
Carton contains 56 tablets and 56 capsules

14 DAILY TREATMENT PACKS
TRIPLE THERAPY

Phathom.
PHARMACEUTICALS

NDC 81520-255-14
Rx only

VOQUEZNA *TriplePak*™
vonoprazan tablets, 20 mg
amoxicillin capsules, 500 mg
clarithromycin tablets, 500 mg

14 DAILY TREATMENT PACKS | TRIPLE THERAPY

Store between 20°C and 25°C (68°F and 77°F). Brief exposure to 15°C to 30°C (59°F to 86°F) permitted [see USP Controlled Room Temperature]. Protect from light.

See package insert for complete Prescribing Information.

NDC 81520-255-14
Rx only

EACH DAILY TREATMENT PACK CONTAINS:
2 vonoprazan tablets, 20 mg per tablet*
4 amoxicillin capsules, 500 mg per capsule**
2 clarithromycin tablets, 500 mg per tablet

*Each tablet contains 20 mg of vonoprazan (equivalent to 26.72 mg of vonoprazan fumarate).
**Each capsule contains amoxicillin trihydrate (equivalent to 500 mg amoxicillin).

Vonoprazan tablets manufactured for Phathom Pharmaceuticals, Inc., Buffalo Grove, IL 60089, U.S.A. Product of Ireland.

Amoxicillin capsules, manufactured for Sandoz Inc., Princeton, NJ 08540, U.S.A. Product of Spain.

Clarithromycin tablets, manufactured for Sandoz Inc., Princeton, NJ 08540, U.S.A. Product of China.

Phathom.
PHARMACEUTICALS

0 381520 255140

NDC 81520-255-14
Rx only

VOQUEZNA *TriplePak*™
vonoprazan tablets, 20 mg
amoxicillin capsules, 500 mg
clarithromycin tablets, 500 mg

14 DAILY TREATMENT PACKS
TRIPLE THERAPY

Phathom.
PHARMACEUTICALS

GTIN: 01234567891234
S/N: 13854785162804
EXP: YYYY MMM
LOT: 12345678AB

NDC 81520-255-02

Rx only

VOQUEZNA *TriplePak*™

vonoprazan tablets, 20 mg
amoxicillin capsules, 500 mg
clarithromycin tablets, 500 mg

PROFESSIONAL SAMPLE – NOT FOR RESALE

TREATMENT PACK FOR ONE DAY OF THERAPY

2 vonoprazan tablets, 20 mg per tablet*
2 clarithromycin tablets, 500 mg per tablet
4 amoxicillin capsules, 500 mg per capsule**

*Each tablet contains 20 mg of vonoprazan (equivalent to 26.72 mg of vonoprazan fumarate).
**Each capsule contains amoxicillin trihydrate (equivalent to 500 mg amoxicillin).

KEEP OUT OF REACH OF CHILDREN.

For further information, please see complete Prescribing Information.

AM

PM

NDC 81520-255-02

Rx only

VOQUEZNA *TriplePak*™

vonoprazan tablets, 20 mg
amoxicillin capsules, 500 mg
clarithromycin tablets, 500 mg

PROFESSIONAL SAMPLE – NOT FOR RESALE

TREATMENT PACK FOR ONE DAY OF THERAPY

2 vonoprazan tablets, 20 mg per tablet*
2 clarithromycin tablets, 500 mg per tablet
4 amoxicillin capsules, 500 mg per capsule**

*Each tablet contains 20 mg of vonoprazan (equivalent to 26.72 mg of vonoprazan fumarate).
**Each capsule contains amoxicillin trihydrate (equivalent to 500 mg amoxicillin).

KEEP OUT OF REACH OF CHILDREN.

For further information, please see complete Prescribing Information.

AM

PM

VOQUEZNA *TriplePak*™

vonoprazan tablets, 20 mg
amoxicillin capsules, 500 mg
clarithromycin tablets, 500 mg

UPC FPO

PATIENT INSTRUCTIONS FOR ONE DAY OF THERAPY

Directions:

1. Cut off the AM doses along the dashed scissors line on the back of the card.
2. Looking at the front of the AM doses, carefully cut along the long side of one capsule/tablet creating an opening and peel back the foil. Take the medication with liquid, with or without food.
3. Repeat step 2 for each of the remaining AM doses.
4. In the evening (12 hours later), repeat the steps above using the PM section of this pack to take the remaining medications.

Store between 20°C and 25°C (68°F and 77°F).
Brief exposure to 15°C to 30°C (59°F to 86°F) permitted [see USP Controlled Room Temperature].
Protect from light.

46295926

LOT: 12345678AB EXP: YYYY MMM

PM

Vonoprazan Tablet, 20 mg
Manufactured for Phathom Pharmaceuticals, Inc. Buffalo Grove, IL 60089, U.S.A.
Product of Ireland

Clarithromycin Tablet, 500 mg
Manufactured for Sandoz Inc., Princeton, NJ 08540, U.S.A.
Product of China

Amoxicillin Capsule, 500 mg
Manufactured for Sandoz Inc, Princeton, NJ 08540, U.S.A.
Product of Spain

Amoxicillin Capsule, 500 mg
Manufactured for Sandoz Inc, Princeton, NJ 08540, U.S.A.
Product of Spain

AM

Vonoprazan Tablet, 20 mg
Manufactured for Phathom Pharmaceuticals, Inc. Buffalo Grove, IL 60089, U.S.A.
Product of Ireland

Clarithromycin Tablet, 500 mg
Manufactured for Sandoz Inc., Princeton, NJ 08540, U.S.A.
Product of China

Amoxicillin Capsule, 500 mg
Manufactured for Sandoz Inc, Princeton, NJ 08540, U.S.A.
Product of Spain

Amoxicillin Capsule, 500 mg
Manufactured for Sandoz Inc, Princeton, NJ 08540, U.S.A.
Product of Spain

LOT: 12345678AB EXP: YYYY MMM

VOQUEZNA *TriplePak*™

vonoprazan tablets, 20 mg
amoxicillin capsules, 500 mg
clarithromycin tablets, 500 mg

UPC FPO

PATIENT INSTRUCTIONS FOR ONE DAY OF THERAPY

Directions:

1. Cut off the AM doses along the dashed scissors line on the back of the card.
2. Looking at the front of the AM doses, carefully cut along the long side of one capsule/tablet creating an opening and peel back the foil. Take the medication with liquid, with or without food.
3. Repeat step 2 for each of the remaining AM doses.
4. In the evening (12 hours later), repeat the steps above using the PM section of this pack to take the remaining medications.

Store between 20°C and 25°C (68°F and 77°F).
Brief exposure to 15°C to 30°C (59°F to 86°F) permitted [see USP Controlled Room Temperature].
Protect from light.

46295926

LOT: 12345678AB EXP: YYYY MMM

PM

Vonoprazan Tablet, 20 mg
Manufactured for Phathom Pharmaceuticals, Inc. Buffalo Grove, IL 60089, U.S.A.
Product of Ireland

Clarithromycin Tablet, 500 mg
Manufactured for Sandoz Inc., Princeton, NJ 08540, U.S.A.
Product of China

Amoxicillin Capsule, 500 mg
Manufactured for Sandoz Inc, Princeton, NJ 08540, U.S.A.
Product of Spain

Amoxicillin Capsule, 500 mg
Manufactured for Sandoz Inc, Princeton, NJ 08540, U.S.A.
Product of Spain

AM

Vonoprazan Tablet, 20 mg
Manufactured for Phathom Pharmaceuticals, Inc. Buffalo Grove, IL 60089, U.S.A.
Product of Ireland

Clarithromycin Tablet, 500 mg
Manufactured for Sandoz Inc., Princeton, NJ 08540, U.S.A.
Product of China

Amoxicillin Capsule, 500 mg
Manufactured for Sandoz Inc, Princeton, NJ 08540, U.S.A.
Product of Spain

Amoxicillin Capsule, 500 mg
Manufactured for Sandoz Inc, Princeton, NJ 08540, U.S.A.
Product of Spain

LOT: 12345678AB EXP: YYYY MMM

NDC 81520-255-01

Rx only

VOQUEZNA *TriplePak*™

vonoprazan tablets, 20 mg
amoxicillin capsules, 500 mg
clarithromycin tablets, 500 mg

TREATMENT PACK FOR ONE DAY OF THERAPY

2 vonoprazan tablets, 20 mg per tablet*
2 clarithromycin tablets, 500 mg per tablet
4 amoxicillin capsules, 500 mg per capsule**

*Each tablet contains 20 mg of vonoprazan (equivalent to 26.72 mg of vonoprazan fumarate).
**Each capsule contains amoxicillin trihydrate (equivalent to 500 mg amoxicillin).

KEEP OUT OF REACH OF CHILDREN.

For further information, please see complete Prescribing Information.

AM

PM

NDC 81520-255-01

Rx only

VOQUEZNA *TriplePak*™

vonoprazan tablets, 20 mg
amoxicillin capsules, 500 mg
clarithromycin tablets, 500 mg

TREATMENT PACK FOR ONE DAY OF THERAPY

2 vonoprazan tablets, 20 mg per tablet*
2 clarithromycin tablets, 500 mg per tablet
4 amoxicillin capsules, 500 mg per capsule**

*Each tablet contains 20 mg of vonoprazan (equivalent to 26.72 mg of vonoprazan fumarate).
**Each capsule contains amoxicillin trihydrate (equivalent to 500 mg amoxicillin).

KEEP OUT OF REACH OF CHILDREN.

For further information, please see complete Prescribing Information.

AM

PM

VOQUEZNA *TriplePak*™

vonoprazan tablets, 20 mg
amoxicillin capsules, 500 mg
clarithromycin tablets, 500 mg

UPC FPO

PATIENT INSTRUCTIONS FOR ONE DAY OF THERAPY

Directions:

1. Cut off the AM doses along the dashed scissors line on the back of the card.
2. Looking at the front of the AM doses, carefully cut along the long side of one capsule/tablet creating an opening and peel back the foil. Take the medication with liquid, with or without food.
3. Repeat step 2 for each of the remaining AM doses.
4. In the evening (12 hours later), repeat the steps above using the PM section of this pack to take the remaining medications.

Store between 20°C and 25°C (68°F and 77°F).
Brief exposure to 15°C to 30°C (59°F to 86°F) permitted [see USP Controlled Room Temperature].
Protect from light.

46285513

LOT: 12345678AB EXP: YYYY MMM

PM

Vonoprazan Tablet, 20 mg
Manufactured for Phathom Pharmaceuticals, Inc. Buffalo Grove, IL 60089, U.S.A.
Product of Ireland

Clarithromycin Tablet, 500 mg
Manufactured for Sandoz Inc., Princeton, NJ 08540, U.S.A.
Product of China

Amoxicillin Capsule, 500 mg
Manufactured for Sandoz Inc, Princeton, NJ 08540, U.S.A.
Product of Spain

Amoxicillin Capsule, 500 mg
Manufactured for Sandoz Inc, Princeton, NJ 08540, U.S.A.
Product of Spain

AM

Vonoprazan Tablet, 20 mg
Manufactured for Phathom Pharmaceuticals, Inc. Buffalo Grove, IL 60089, U.S.A.
Product of Ireland

Clarithromycin Tablet, 500 mg
Manufactured for Sandoz Inc., Princeton, NJ 08540, U.S.A.
Product of China

Amoxicillin Capsule, 500 mg
Manufactured for Sandoz Inc, Princeton, NJ 08540, U.S.A.
Product of Spain

Amoxicillin Capsule, 500 mg
Manufactured for Sandoz Inc, Princeton, NJ 08540, U.S.A.
Product of Spain

LOT: 12345678AB EXP: YYYY MMM

VOQUEZNA *TriplePak*™

vonoprazan tablets, 20 mg
amoxicillin capsules, 500 mg
clarithromycin tablets, 500 mg

UPC FPO

PATIENT INSTRUCTIONS FOR ONE DAY OF THERAPY

Directions:

1. Cut off the AM doses along the dashed scissors line on the back of the card.
2. Looking at the front of the AM doses, carefully cut along the long side of one capsule/tablet creating an opening and peel back the foil. Take the medication with liquid, with or without food.
3. Repeat step 2 for each of the remaining AM doses.
4. In the evening (12 hours later), repeat the steps above using the PM section of this pack to take the remaining medications.

Store between 20°C and 25°C (68°F and 77°F).
Brief exposure to 15°C to 30°C (59°F to 86°F) permitted [see USP Controlled Room Temperature].
Protect from light.

46285513

LOT: 12345678AB EXP: YYYY MMM

PM

Vonoprazan Tablet, 20 mg
Manufactured for Phathom Pharmaceuticals, Inc. Buffalo Grove, IL 60089, U.S.A.
Product of Ireland

Clarithromycin Tablet, 500 mg
Manufactured for Sandoz Inc., Princeton, NJ 08540, U.S.A.
Product of China

Amoxicillin Capsule, 500 mg
Manufactured for Sandoz Inc, Princeton, NJ 08540, U.S.A.
Product of Spain

Amoxicillin Capsule, 500 mg
Manufactured for Sandoz Inc, Princeton, NJ 08540, U.S.A.
Product of Spain

AM

Vonoprazan Tablet, 20 mg
Manufactured for Phathom Pharmaceuticals, Inc. Buffalo Grove, IL 60089, U.S.A.
Product of Ireland

Clarithromycin Tablet, 500 mg
Manufactured for Sandoz Inc., Princeton, NJ 08540, U.S.A.
Product of China

Amoxicillin Capsule, 500 mg
Manufactured for Sandoz Inc, Princeton, NJ 08540, U.S.A.
Product of Spain

Amoxicillin Capsule, 500 mg
Manufactured for Sandoz Inc, Princeton, NJ 08540, U.S.A.
Product of Spain

LOT: 12345678AB EXP: YYYY MMM

NDC 81520-250-00
Rx only

VOQUEZNA DualPak™
vonoprazan tablets, 20 mg
amoxicillin capsules, 500 mg

14 DAILY TREATMENT PACKS
DUAL THERAPY

PROFESSIONAL SAMPLE – NOT FOR RESALE

Phathom PHARMACEUTICALS

EXP: YYYY MMM
LOT: 12345678AB

46297838

NDC 81520-250-00
Rx only

VOQUEZNA DualPak™
vonoprazan tablets, 20 mg
amoxicillin capsules, 500 mg

14 DAILY TREATMENT PACKS | DUAL THERAPY

PROFESSIONAL SAMPLE – NOT FOR RESALE

KEEP OUT OF REACH OF CHILDREN

VOQUEZNA DUAL PAK is distributed by Phathom Pharmaceuticals, Inc. Buffalo Grove, IL 60089, U.S.A.

VOQUEZNA, VOQUEZNA DUAL PAK, and the VOQUEZNA logo are trademarks of Phathom Pharmaceuticals, Inc.

NDC 81520-250-00
Rx only

VOQUEZNA DualPak™
vonoprazan tablets, 20 mg
amoxicillin capsules, 500 mg

Co-packaged for oral use

Carton contains 28 tablets and 84 capsules

14 DAILY TREATMENT PACKS
DUAL THERAPY

PROFESSIONAL SAMPLE – NOT FOR RESALE

Phathom PHARMACEUTICALS

NDC 81520-250-00
Rx only

VOQUEZNA DualPak™
vonoprazan tablets, 20 mg
amoxicillin capsules, 500 mg

14 DAILY TREATMENT PACKS | DUAL THERAPY

PROFESSIONAL SAMPLE – NOT FOR RESALE

Store between 20°C and 25°C (68°F and 77°F).
Brief exposure to 15°C to 30°C (59°F to 86°F) permitted [see USP Controlled Room Temperature].

See package insert for complete Prescribing Information.

NDC 81520-250-00
Rx only

EACH DAILY TREATMENT PACK CONTAINS:
2 vonoprazan tablets, 20 mg per tablet*
6 amoxicillin capsules, 500 mg per capsule**

*Each tablet contains 20 mg of vonoprazan (equivalent to 26.72 mg of vonoprazan fumarate).
**Each capsule contains amoxicillin trihydrate (equivalent to 500 mg amoxicillin).

PROFESSIONAL SAMPLE – NOT FOR RESALE

Vonoprazan tablets manufactured for Phathom Pharmaceuticals, Inc. Buffalo Grove, IL 60089, U.S.A. Product of Ireland.

Amoxicillin capsules, manufactured for Sandoz Inc., Princeton, NJ 08540, U.S.A. Product of Spain.

Phathom PHARMACEUTICALS

0 381520 250008

GTIN: 01234567891234
S/N: 13854785162804
EXP: YYYY MMM
LOT: 12345678AB

Phathom PHARMACEUTICALS

14 DAILY TREATMENT PACKS
DUAL THERAPY

VOQUEZNA DualPak™
vonoprazan tablets, 20 mg
amoxicillin capsules, 500 mg

NDC 81520-250-14
Rx only

46297698

NDC 81520-250-14
Rx only

VOQUEZNA DualPak™
vonoprazan tablets, 20 mg
amoxicillin capsules, 500 mg

14 DAILY TREATMENT PACKS | DUAL THERAPY

KEEP OUT OF REACH OF CHILDREN

VOQUEZNA DUAL PAK is distributed by Phathom Pharmaceuticals, Inc. Buffalo Grove, IL 60089, U.S.A.

VOQUEZNA, VOQUEZNA DUAL PAK, and the VOQUEZNA logo are trademarks of Phathom Pharmaceuticals, Inc.

NDC 81520-250-14
Rx only

VOQUEZNA DualPak™
vonoprazan tablets, 20 mg
amoxicillin capsules, 500 mg

Co-packaged for oral use

Carton contains 28 tablets and 84 capsules

14 DAILY TREATMENT PACKS
DUAL THERAPY

Phathom PHARMACEUTICALS

NDC 81520-250-14
Rx only

VOQUEZNA DualPak™
vonoprazan tablets, 20 mg
amoxicillin capsules, 500 mg

14 DAILY TREATMENT PACKS | DUAL THERAPY

Store between 20°C and 25°C (68°F and 77°F).
Brief exposure to 15°C to 30°C (59°F to 86°F) permitted
[see USP Controlled Room Temperature].

See package insert for complete Prescribing Information.

NDC 81520-250-14
Rx only

EACH DAILY TREATMENT PACK CONTAINS:

2 vonoprazan tablets, 20 mg per tablet*
6 amoxicillin capsules, 500 mg per capsule**

*Each tablet contains 20 mg of vonoprazan (equivalent to 26.72 mg of vonoprazan fumarate).
**Each capsule contains amoxicillin trihydrate (equivalent to 500 mg amoxicillin).

Vonoprazan tablets manufactured for Phathom Pharmaceuticals, Inc. Buffalo Grove, IL 60089, U.S.A. Product of Ireland.

Amoxicillin capsules, manufactured for Sandoz Inc., Princeton, NJ 08540, U.S.A. Product of Spain.

Phathom PHARMACEUTICALS

0 381520 250145

NDC 81520-250-02
Rx only

VOQUEZNA DualPak™
vonoprazan tablets, 20 mg
amoxicillin capsules, 500 mg

PROFESSIONAL SAMPLE – NOT FOR RESALE

TREATMENT PACK FOR ONE DAY OF THERAPY

2 vonoprazan tablets, 20 mg per tablet*
6 amoxicillin capsules, 500 mg per capsule**

*Each tablet contains 20 mg of vonoprazan (equivalent to 26.72 mg of vonoprazan fumarate).
**Each capsule contains amoxicillin trihydrate (equivalent to 500 mg amoxicillin).

KEEP OUT OF REACH OF CHILDREN.

For further information, please see complete Prescribing Information.

PM

MID-DAY

AM

NDC 81520-250-02
Rx only

VOQUEZNA DualPak™
vonoprazan tablets, 20 mg
amoxicillin capsules, 500 mg

PROFESSIONAL SAMPLE – NOT FOR RESALE

TREATMENT PACK FOR ONE DAY OF THERAPY

2 vonoprazan tablets, 20 mg per tablet*
6 amoxicillin capsules, 500 mg per capsule**

*Each tablet contains 20 mg of vonoprazan (equivalent to 26.72 mg of vonoprazan fumarate).
**Each capsule contains amoxicillin trihydrate (equivalent to 500 mg amoxicillin).

KEEP OUT OF REACH OF CHILDREN.

For further information, please see complete Prescribing Information.

PM

MID-DAY

AM

VOQUEZNA DualPak™
vonoprazan tablets, 20 mg
™ amoxicillin capsules, 500 mg

UPC FPO

PATIENT INSTRUCTIONS FOR ONE DAY OF THERAPY

Directions:

1. Cut off the AM doses along the dashed scissors line on the back of the card.
2. Looking at the front of the AM doses, carefully cut along the long side of one capsule/tablet creating an opening and peel back the foil. Take the medication with liquid, with or without food.
3. Repeat step 2 for each of the remaining AM doses.
4. In the afternoon, repeat the steps above using the MID-DAY section of this pack to take the MID-DAY medications.
5. In the evening, repeat steps 1 – 3 above using the PM section of this pack to take the remaining medications.

Store between 20°C and 25°C (68°F and 77°F).
Brief exposure to 15°C to 30°C (59°F to 86°F) permitted [see USP Controlled Room Temperature].
46297837

LOT: 12345678AB EXP: YYYY MMM

PM

Amoxicillin Capsule, 500 mg
Manufactured for Sandoz Inc, Princeton, NJ 08540, U.S.A.
Product of Spain

Vonoprazan Tablet, 20 mg
Manufactured for Phathom Pharmaceuticals, Inc. Buffalo Grove, IL 60089, U.S.A.
Product of Ireland

Amoxicillin Capsule, 500 mg
Manufactured for Sandoz Inc, Princeton, NJ 08540, U.S.A.
Product of Spain

MID-DAY

Amoxicillin Capsule, 500 mg
Manufactured for Sandoz Inc, Princeton, NJ 08540, U.S.A.
Product of Spain

Amoxicillin Capsule, 500 mg
Manufactured for Sandoz Inc, Princeton, NJ 08540, U.S.A.
Product of Spain

AM

Amoxicillin Capsule, 500 mg
Manufactured for Sandoz Inc, Princeton, NJ 08540, U.S.A.
Product of Spain

Vonoprazan Tablet, 20 mg
Manufactured for Phathom Pharmaceuticals, Inc. Buffalo Grove, IL 60089, U.S.A.
Product of Ireland

Amoxicillin Capsule, 500 mg
Manufactured for Sandoz Inc, Princeton, NJ 08540, U.S.A.
Product of Spain

LOT: 12345678AB EXP: YYYY MMM

VOQUEZNA DualPak™
vonoprazan tablets, 20 mg
™ amoxicillin capsules, 500 mg

UPC FPO

PATIENT INSTRUCTIONS FOR ONE DAY OF THERAPY

Directions:

1. Cut off the AM doses along the dashed scissors line on the back of the card.
2. Looking at the front of the AM doses, carefully cut along the long side of one capsule/tablet creating an opening and peel back the foil. Take the medication with liquid, with or without food.
3. Repeat step 2 for each of the remaining AM doses.
4. In the afternoon, repeat the steps above using the MID-DAY section of this pack to take the MID-DAY medications.
5. In the evening, repeat steps 1 – 3 above using the PM section of this pack to take the remaining medications.

Store between 20°C and 25°C (68°F and 77°F).
Brief exposure to 15°C to 30°C (59°F to 86°F) permitted [see USP Controlled Room Temperature].
46297837

LOT: 12345678AB EXP: YYYY MMM

PM

Amoxicillin Capsule, 500 mg
Manufactured for Sandoz Inc, Princeton, NJ 08540, U.S.A.
Product of Spain

Vonoprazan Tablet, 20 mg
Manufactured for Phathom Pharmaceuticals, Inc. Buffalo Grove, IL 60089, U.S.A.
Product of Ireland

Amoxicillin Capsule, 500 mg
Manufactured for Sandoz Inc, Princeton, NJ 08540, U.S.A.
Product of Spain

MID-DAY

Amoxicillin Capsule, 500 mg
Manufactured for Sandoz Inc, Princeton, NJ 08540, U.S.A.
Product of Spain

Amoxicillin Capsule, 500 mg
Manufactured for Sandoz Inc, Princeton, NJ 08540, U.S.A.
Product of Spain

AM

Amoxicillin Capsule, 500 mg
Manufactured for Sandoz Inc, Princeton, NJ 08540, U.S.A.
Product of Spain

Vonoprazan Tablet, 20 mg
Manufactured for Phathom Pharmaceuticals, Inc. Buffalo Grove, IL 60089, U.S.A.
Product of Ireland

Amoxicillin Capsule, 500 mg
Manufactured for Sandoz Inc, Princeton, NJ 08540, U.S.A.
Product of Spain

LOT: 12345678AB EXP: YYYY MMM

NDC 81520-250-01

Rx only

VOQUEZNA *DualPak*™

vonoprazan tablets, 20 mg
amoxicillin capsules, 500 mg

TREATMENT PACK FOR ONE DAY OF THERAPY

2 vonoprazan tablets, 20 mg per tablet*
6 amoxicillin capsules, 500 mg per capsule**

*Each tablet contains 20 mg of vonoprazan (equivalent to 26.72 mg of vonoprazan fumarate).
**Each capsule contains amoxicillin trihydrate (equivalent to 500 mg amoxicillin).

KEEP OUT OF REACH OF CHILDREN.

For further information, please see complete Prescribing Information.

PM

MID-DAY

AM

NDC 81520-250-01

Rx only

VOQUEZNA *DualPak*™

vonoprazan tablets, 20 mg
amoxicillin capsules, 500 mg

TREATMENT PACK FOR ONE DAY OF THERAPY

2 vonoprazan tablets, 20 mg per tablet*
6 amoxicillin capsules, 500 mg per capsule**

*Each tablet contains 20 mg of vonoprazan (equivalent to 26.72 mg of vonoprazan fumarate).
**Each capsule contains amoxicillin trihydrate (equivalent to 500 mg amoxicillin).

KEEP OUT OF REACH OF CHILDREN.

For further information, please see complete Prescribing Information.

PM

MID-DAY

AM

VOQUEZNA *DualPak*™
vonoprazan tablets, 20 mg
amoxicillin capsules, 500 mg

UPC FPO

PATIENT INSTRUCTIONS FOR ONE DAY OF THERAPY

Directions:

1. Cut off the AM doses along the dashed scissors line on the back of the card.
2. Looking at the front of the AM doses, carefully cut along the long side of one capsule/tablet creating an opening and peel back the foil. Take the medication with liquid, with or without food.
3. Repeat step 2 for each of the remaining AM doses.
4. In the afternoon, repeat the steps above using the MID-DAY section of this pack to take the MID-DAY medications.
5. In the evening, repeat steps 1 – 3 above using the PM section of this pack to take the remaining medications.

Store between 20°C and 25°C (68°F and 77°F).
Brief exposure to 15°C to 30°C (59°F to 86°F) permitted
[see USP Controlled Room Temperature]. 46297699

LOT: 12345678AB EXP: YYYY MMM

PM

Amoxicillin Capsule, 500 mg
Manufactured for Sandoz Inc, Princeton, NJ 08540, U.S.A.
Product of Spain

Vonoprazan Tablet, 20 mg
Manufactured for Phathom Pharmaceuticals, Inc. Buffalo Grove, IL 60089, U.S.A.
Product of Ireland

Amoxicillin Capsule, 500 mg
Manufactured for Sandoz Inc, Princeton, NJ 08540, U.S.A.
Product of Spain

MID-DAY

Amoxicillin Capsule, 500 mg
Manufactured for Sandoz Inc, Princeton, NJ 08540, U.S.A.
Product of Spain

Amoxicillin Capsule, 500 mg
Manufactured for Sandoz Inc, Princeton, NJ 08540, U.S.A.
Product of Spain

AM

Amoxicillin Capsule, 500 mg
Manufactured for Sandoz Inc, Princeton, NJ 08540, U.S.A.
Product of Spain

Vonoprazan Tablet, 20 mg
Manufactured for Phathom Pharmaceuticals, Inc. Buffalo Grove, IL 60089, U.S.A.
Product of Ireland

Amoxicillin Capsule, 500 mg
Manufactured for Sandoz Inc, Princeton, NJ 08540, U.S.A.
Product of Spain

LOT: 12345678AB EXP: YYYY MMM

VOQUEZNA *DualPak*™
vonoprazan tablets, 20 mg
amoxicillin capsules, 500 mg

UPC FPO

PATIENT INSTRUCTIONS FOR ONE DAY OF THERAPY

Directions:

1. Cut off the AM doses along the dashed scissors line on the back of the card.
2. Looking at the front of the AM doses, carefully cut along the long side of one capsule/tablet creating an opening and peel back the foil. Take the medication with liquid, with or without food.
3. Repeat step 2 for each of the remaining AM doses.
4. In the afternoon, repeat the steps above using the MID-DAY section of this pack to take the MID-DAY medications.
5. In the evening, repeat steps 1 – 3 above using the PM section of this pack to take the remaining medications.

Store between 20°C and 25°C (68°F and 77°F).
Brief exposure to 15°C to 30°C (59°F to 86°F) permitted
[see USP Controlled Room Temperature]. 46297699

LOT: 12345678AB EXP: YYYY MMM

PM

Amoxicillin Capsule, 500 mg
Manufactured for Sandoz Inc, Princeton, NJ 08540, U.S.A.
Product of Spain

Vonoprazan Tablet, 20 mg
Manufactured for Phathom Pharmaceuticals, Inc. Buffalo Grove, IL 60089, U.S.A.
Product of Ireland

Amoxicillin Capsule, 500 mg
Manufactured for Sandoz Inc, Princeton, NJ 08540, U.S.A.
Product of Spain

MID-DAY

Amoxicillin Capsule, 500 mg
Manufactured for Sandoz Inc, Princeton, NJ 08540, U.S.A.
Product of Spain

Amoxicillin Capsule, 500 mg
Manufactured for Sandoz Inc, Princeton, NJ 08540, U.S.A.
Product of Spain

AM

Amoxicillin Capsule, 500 mg
Manufactured for Sandoz Inc, Princeton, NJ 08540, U.S.A.
Product of Spain

Vonoprazan Tablet, 20 mg
Manufactured for Phathom Pharmaceuticals, Inc. Buffalo Grove, IL 60089, U.S.A.
Product of Ireland

Amoxicillin Capsule, 500 mg
Manufactured for Sandoz Inc, Princeton, NJ 08540, U.S.A.
Product of Spain

LOT: 12345678AB EXP: YYYY MMM

This is a representation of an electronic record that was signed electronically. Following this are manifestations of any and all electronic signatures for this electronic record.

/s/

JOHN J FARLEY
05/03/2022 04:37:30 PM
